# Supplementary material for: Immunologic effects of locoregional therapies for unresectable hepatocellular carcinoma
Source: JHEP Rep. 2025 Aug 22;7(12):101555. doi: 10.1016/j.jhepr.2025.101555 (PMC12637058; doi:10.1016/j.jhepr.2025.101555)
Supplement: Multimedia component 1 [file mmc1.pdf]

# Immunologic effects of locoregional therapies for unresectable hepatocellular carcinoma

Robin Schmidt, Bernhard Gebauer, Nilufar Akbari, Christoph Roderburg, Giovanni Federico Torsello, Uli Fehrenbach, Timo Alexander Auer, Raphael Mohr, Gracia Lana Ardila Pardo, Winna Lim, Fabio Pivetta, Elif Can, Charlie Alexander Hamm, Frank Tacke, Bernd Hamm, Linda Hammerich, Lynn Jeanette Savic

Table of contents

Supplementary methods……………………………………………………………….…….....2

Supplementary tables…………………………………………………………..……..…..6

Supplementary figures…………………………………………………………..………25

Supplementary figures legends…………………………………………………..…….30

Supplementary references………………………………………………………..…….33

## Supplementary methods

*Immunostaining and fluorescence-activated cell sorting (FACS)*

Blood was collected in ethylenediaminetetraacetic acid-containing tubes, immediately stored at 4° Celsius, and processed for further analysis within a maximum of six hours. A total of 100 µl of whole blood were used for flow cytometry; the remaining sample volume was centrifuged at 3000 rpm for 10 min and the supernatant plasma was archived for further analyses. Viability stain (Fixable viability stain 780, BD Biosciences, Franklin Lake, NJ, USA) was added directly to the whole blood at a dilution of 1:1000 followed by a mix of fluorochrome-conjugated antibodies in FACS blocking buffer (made in house). The staining master mix included monoclonal antibodies against CD1c (L161), CD3 (OKT3), CD11b (M1/70), CD11c (Bu15), CD14 (M5E2), CD16 (3G8), CD19 (HIB19), CD38 (HIT2), CD56 (5.1H11), CD66b (G10F5), CD141 (M80), CTLA-4 (L3D10), HLA-DR (L243), PD1 (NAT105), PD-L1 (29E.2A3), TIM3 (F38-2E2), XCR1 (S15046E, all BioLegend, San Diego, CA, USA), CD4 (RPA-T4) and CD8 (RPA-T8, both BD BioSciences, Franklin Lake, NJ, USA), CD45 (HI30, Thermo Fisher, Waltham, MA, USA), and LAG3 (3DS223H, Thermo Fisher, Waltham, MA, USA). Fluorochrome-Minus-One controls were included for checkpoints, activation, and exhaustion markers. Samples were incubated for 20 minutes at room temperature followed by red blood cell lysis using lysis buffer (1X BD Pharm Lyse, BD Biosciences, Franklin Lake, NJ, USA) for 10 min and centrifugation (500 g, 5 min, 4° Celsius). The supernatant was discarded, and lysis was performed a second time. Samples were fixed with 2% formalin for 10 min at room temperature, washed with PBS and resuspended in 200 µl of PBS for analysis. Additionally, 10 µl of Precision Count Beads™ (BioLegend, San Diego, CA, USA) were added directly before measurement.

Immune cell gating strategy

Samples were analyzed using a Cytek® Aurora flow cytometer (Cytek Biosciences, Fremont, CA, USA). Unmixing and spill-over-correction was performed in SpectroFlo software (Cytek Biosciences, Fremont, CA, USA). Data was analyzed using FCS Express V7 (De Novo Software, Pasadena, CA, USA) with regards to $CD45^+$ lymphoid and myeloid immune cell populations. [1] Gating strategy is depicted in the Supplement Figure 1. Cells were grouped into three morphological subset profiles of 1) T cell response , 2) recruiting and antigen-presenting, and 3) inflammation and phagocytosis, consisting of the following phenotypes: 1) T cells ($CD3^+CD56^-/CD19^-$), and NK-T cells ($CD56^+$ T cells), 2) B cells ($CD19^+/CD3^-/CD56^-$), monocytes ($CD14^+/CD16^+/CD66b^-$), and conventional dendritic cells ($CD11c^+/HLA\text{-}DR^+/CD14^-/CD16^-$), and 3) NK cells ($CD56^+/CD3^-/CD19^-$), neutrophils ($CD66^+/CD11b^+/CD16b^+/CD14^-$) and eosinophils ($CD66^+/CD11b^+/CD16^{low}/CD14^-$). Further markers were used for phenotyping subpopulations such as T-helper cells ($CD4^+$ T cells) and cytotoxic T cells ($CD8^+$ T cells), $CD56^{bright}$ NK cells ($CD56^{high}/CD16^{low}$) and $CD56^{diminished}$ NK cells ($CD56^{low}/CD16^+$), or classical monocytes ($CD14^{high}/CD16^+$) and non-classical monocytes ($CD14^{low}/CD16^+$), as they are described in literature elsewhere. [2-4] In addition, checkpoints, and exhaustion marker profiles e.g., CTLA-4, PD1, PDL-1, TIM3, LAG3 and HLA-DR, CD38, CD161 were assessed on these subpopulations (Supplement Figure 2). Immune cell counts were quantified according to the gating strategy and reported for each time point in absolute and relative numbers. Relative numbers were calculated for each immune subpopulation and the proportion was reported as percentage of all cells in the mother gate (e.g. proportion of $CD4^+$ T cells of all T cells).

*Locoregional therapies (LRT)*

*Conventional transarterial chemoembolization*: Selective cTACE was performed using 5 cc of chemotherapy (50 mg doxorubicin in 2.5 cc and 10 mg of mitomycin-C in 2.5 cc) mixed 1:2 with Lipiodol® (Guerbet, Villepinte, France) in a homogenous emulsion [5]. The catheter was inserted through the femoral artery and placed in the tumor feeding artery branches to achieve selective embolization. The emulsion was administered under fluoroscopic guidance into the tumor-feeding arteries, followed by injection of sterile compressed sponge (Gelfoam, Pfizer, New York, NY, USA) until blood flow stasis was reached. In the case of a cTACE/iBT combination therapy, cTACE was performed 24 hours prior to iBT. During iBT, Lipiodol® deposition was visually assessed and aided the catheter placement.

*CT-guided interstitial high dose-rate brachytherapy:* iBT was performed as described elsewhere [6, 7]. Briefly, iBT was performed under conscious sedation with midazolam and fentanyl, and local anesthesia with lidocaine. Under CT-fluoroscopy, a 6F angiographic sheath was inserted into the lesion, and a closed-end 6F brachytherapy catheter was put through the sheath. For 3D radiation planning, a contrast-enhanced CT scan including at least an arterial and venous phase (15 s and 50 s after injection, respectively) was acquired to depict the catheter in relation to the tumor (slice thickness of 1 mm and 5 mm). The clinical target lesion volume was manually segmented. Radiation was performed using an iridium-192 source enclosing a target dose of 20 Gy for each lesion. Adjacent structures at risk, such as stomach or duodenum, were manually marked and dosages were calculated. Where necessary, overall dosage was modified as described elsewhere [8].

Image acquisition:

MRI scans were obtained on 1,5-T (Avanto, Aera), or 3-T scanners (Skyra; all from Siemens, Munich, Germany) using an eight-channel body phased-array coil. The standard imaging protocol included breath-hold un- and contrast-enhanced T1-weighted sequences (VIBE, in-phase/opposed-phase FLASH) with a hepatocyte-specific contrast agent being applied (0,1 ml/kg intravenous Primovist; Bayer, Germany), T2-weighted sequences (HASTE, TSE), and diffusion-weighted imaging (DWI) with apparent diffusion coefficient (ADC) maps. Dynamic contrast-enhanced images included arterial, portalvenous, venous and hepatobiliary phase (15, 50, 90 seconds and 20 minute after contrast administration, respectively) that were obtained in the axial plane covering the entire liver with 60-72 slices and an adjusted field of view of 255 – 300 mm x 340 – 400 mm (TR 4.26 ms, TE 1.87 ms, flip angle 10°, slice thickness 3 mm, matrix size 256 x 127).

CT scans after cTACE were obtained without contrast agent. Axial slices of 5 mm slice thickness were obtained with field of view of 39,5 mm using a standard kernel within a 512 x 512 matrix, followed by the reconstruction of 0.625-1.0 mm axial slices with multiplanar reconstructions.

## Supplementary tables

**Table S1:** Absolute immune cell counts. Cell concentrations are depicted as n/µl.

| | cTACE | | | iBT | | | cTACE/iBT | | |
|---|---|---|---|---|---|---|---|---|---|
| Time Points | Baseline | One day post | Two months post | Baseline | One day post | Two months post | Baseline | One day post | Two months post |
| n | 27 | 26 | 17 | 68 | 68 | 52 | 43 | 43 | 25 |
| **Leucocytes** | **2095.9** [1345.7, 245.4] | **3938.8** [2568.9, 460.5] | **3334.3** [1767.3,3683.8] | **2902.4** [1917.8, 4161.5] | **2826.4** [2241.9, 4668.8] | **2774.5** [1872.4, 4171.5] | **2743.2** [1772.6, 4339.2] | **3169.4** [2354.8, 5333.3] | **2265.4** [1395.7, 3218.2] |
| **p-values** | **p<0.01** | | **p<0.01** | **p=0.04** | | p=0.09 | p=0.69 | | **p=0.03** |
| **Lymphocytes** | **553.1** [410.3, 1177.9] | **418.2** [275.5, 685.6] | **917.4** [487.9, 1310.8] | **765.4** [549.3, 1200.4] | **629.9** [325.5, 843.0] | **805.5** [511.5, 1172.5] | **791.0** [593.9, 1014.0] | **533.6** [361.1, 759.7] | **754.3** [488.8, 898.2] |
| **p-values** | **p<0.01** | | **p<0.01** | **p<0.01** | | **p<0.01** | **p=0.01** | | p=0.65 |
| **Myeloid Cells** | **1530.9** [795.9, 2347.3] | **3369.4** [2232.9, 5909.8] | **2023.1** [921.4, 2677.8] | **2223.5** [1370.3, 3145.7] | **2188.4** [1789.4 3887.3] | **1949.5** [1174.4, 2885.7] | **2120.8** [1174.1, 3404.5] | **2506.6** [1847.3, 4380 | **1590.2** [1040.6, 2685.9] |

| | | | | | | | | | |
|---|---|---|---|---|---|---|---|---|---|
| | | | | | | | | .0] | |
| **p-values** | **p<0.01** | | **p<0.01** | **p<0.01** | | **p=0.01** | p=0.33 | | **p=0.01** |
| **Classical T cells** | **370.7** [220.8, 819.2] | **216.7** [136.9, 400.2] | **621.2** [264.7, 955.3] | **478.9** [333.9, 768.5] | **369.2** [168.9, 504.6] | **501.6** [282.8, 647.6] | **475.6** [343.9, 689.7] | **328.1** [215.4, 498.0] | **391.8** [289.3, 648.8] |
| **p-values** | **p<0.01** | | **p<0.01** | **p<0.01** | | **p<0.01** | **p=0.01** | | p=0.52 |
| **CD4$^+$ T cells** | **239.4** [169.9, 484.9] | **162.8** [83.1, 226.9] | **513.9** [182.6, 671.1] | **335.3** [192.9, 439.9] | **233.7** [105.2, 304.6] | **322.7** [176.9, 464.8] | **340.2** [256.5, 483.8] | **224.4** [147.4, 298.5] | **301.7** [223.8, 407.8] |
| **p-values** | **p<0.01** | | **p<0.01** | **p<0.01** | | **p<0.01** | **p=0.05** | | p=0.96 |
| **CD8$^+$ T cells** | **78.8** [45.3, 130.1] | **49.5** [27.9, 106.5] | **76.5** [59.0, 182.9] | **113.4** [47.2, 217.9] | **80.6** [31.4, 155.0] | **101.4** [41.1, 222.8] | **100.6** [44.5, 164.2] | **64.8** [37.2, 113.9] | **67.2** [35.7, 136.2] |
| **p-values** | **p<0.01** | | **p<0.01** | **p<0.01** | | **p<0.01** | **p<0.01** | | p=0.34 |
| **CD4$^+$ CD8$^+$ T cells** | **1.9** [0.9, 2.9] | **0.9** [0.5, 2.1] | **1.8** [0.9, 3.8] | **2.9** [1.5, 5.8] | **1.9** [0.9, 5.1] | **2.1** [1.2, 5.9] | **2.4** [1.6, 8.2] | **1.8** [0.9, 5.0] | **2.6** [1.0, 7.5] |

| | | | | | | | | | |
|---|---|---|---|---|---|---|---|---|---|
| **p-values** | **p<0.01** | | **p<0.01** | **p<0.01** | | **p<0.01** | **p=0.03** | | p=0.67 |
| **CD4$^-$ CD8$^-$ T cells** | **10.8** [8.5, 24.4] | **8.8** [4.0, 19.9] | **13.7** [6.7, 23.4] | **12.7** [6.3, 19.8] | **6.6** [4.0, 17.7] | **11.1** [6.8, 23.0] | **10.9** [6.7, 16.2] | **6.9** [3.9, 11.8] | **8.5** [4.9, 14.7] |
| **p-values** | **p<0.01** | | **p<0.01** | **p<0.01** | | **p<0.01** | **p<0.01** | | **p<0.01** |
| **NK T cells** | **22.9** [9.6, 52.5] | **13.7** [6.6, 35.3] | **26.8** [15.4, 56.9] | **18.6** [7.7, 42.7] | **12.3** [5.1, 35.5] | **13.5** [5.7, 44.5] | **11.1** [4.7, 22.3] | **7.1** [3.9, 17.0] | **9.5** [5.4, 27.5] |
| **p-values** | **p=0.01** | | **p=0.01** | **p=0.03** | | **p=0.05** | **p=0.01** | | **p=0.04** |
| **CD4$^+$ NK T cells** | **2.4** [1.2, 5.2] | **1.0** [0.2, 3.3] | **4.3** [2.5, 6.4] | **1.3** [0.7, 2.5] | **0.9** [0,3 2.0] | **1.4** [0.4, 3.6] | **1.6** [0.6, 2.5] | **0.8** [0.4, 2.2] | **1.1** [0.5, 2.56] |
| **p-values** | **p=0.03** | | **p=0.02** | **p=0.01** | | **p<0.01** | p=0.39 | | p=0.81 |
| **CD8$^+$ NK T cells** | **7.4** [3.5, 17.8] | **5.9** [2.0, 12.7] | **7.3** [3.3, 16.8] | **7.2** [3.2, 21.3] | **5.4** [2.2, 14.8] | **5.2** [1.6, 24.9] | **5.6** [2.2, 9.4] | **2.5** [1.6, 6.9] | **4.3** [1.7, 22.1] |
| **p-values** | **p<0.01** | | **p=0.02** | **p=0.03** | | p=0.57 | **p<0.01** | | p=0.16 |
| **NK cells** | **71.4** [43.5, 141.5] | **75.1** [34.6, 124.2] | **75.5** [51.3, 132.8] | **114.0** [72.4, 184.6] | **84.1** [49.0, 137.4] | **104.2** [71.9, 183.6] | **120.7** [61.5, 207.5] | **49.6** [31.6, 101.0] | **109.1** [57.5, 175.8] |
| **p-values** | p=0.52 | | p=0.66 | **p<0.01** | | **p<0.01** | **p<0.01** | | **p<0.01** |

| | | | | | | | | | |
|---|---|---|---|---|---|---|---|---|---|
| **CD56bright NK cells** | **6.6** [3.7, 10.8] | **3.2** [1.8, 5.2] | **10.4** [5.0, 20.2] | **7.7** [5.4, 10.8] | **4.7** [3.1, 6.3] | **9.6** [6.7, 13.3] | **9.0** [6.4, 12.3] | **3.2** [1.8, 6.5] | **8.7** [6.0, 14.4] |
| p-values | **p<0.01** | | **p<0.01** | **p<0.01** | | **p<0.01** | **p<0.01** | | **p<0.01** |
| **CD56diminished NK cells** | **62.2** [39.2, 134.3] | **66.8** [25.9, 118.3] | **60.3** [45.7, 116.2] | **106.6** [64.3, 174.2] | **80.2** [42.2, 129.6] | **92.9** [58.4, 169.4] | **112.5** [55.0, 186.7] | **46.5** [27.4, 91.9] | **103.1** [45.7, 165.3] |
| p-values | p=0.94 | | p=0.76 | **p<0.01** | | **p<0.01** | **p<0.01** | | **p<0.01** |
| **B cells** | **51.6** [40.8, 121.1] | **55.4** [33.9, 117.8] | **76.2** [40.2, 101.1] | **76.9** [43.6, 113.0] | **42.7** [23.9, 75.9] | **53.3** [32.8, 96.4] | **99.3** [68.0, 144.5] | **59.2** [27.8, 136.7] | **46.9** [28.4, 83.7] |
| p-values | p=0.41 | | p=0.09 | **p<0.01** | | **p=0.01** | p=0.37 | | **p<0.01** |
| **Dendritic Cells (cDC)** | **1.5** [0.8, 3.4] | **0.4** [0.2, 1.6] | **1.9** [1.6, 4.0] | **3.3** [1.8, 5.2] | **2.3** [1.1, 4.9] | **2.9** [2.0, 4.3] | **3.5** [2.1, 5.6] | **1.6** [0.9, 2.7] | **2.8** [1.4, 5.2] |
| p-values | **p<0.01** | | **p<0.01** | p=0.38 | | p=0.23 | **p=0.01** | | **p<0.01** |
| **Type 1 cDCs** | **0.7** [0.2, 1.1] | **0.2** [0.1, 0.5] | **0.8** [0.5, 1.4] | **1.1** [0.8, 1.9] | **0.8** [0.4, 1.7] | **0.9** [0.6, 1.7] | **0.9** [0.6, 2.3] | **0.5** [0.3, 0.8] | **1.3** [0.4, 1.6] |
| p-values | **p<0.01** | | **p<0.01** | p=0.51 | | p=0.21 | **p<0.01** | | **p=0.01** |

| | | | | | | | | | |
|---|---|---|---|---|---|---|---|---|---|
| **Type 2 cDCs** | **1.1** [0.4, 2.4] | **0.2** [0.1, 1.2] | **1.3** [0.6, 2.7] | **2.1** [0.9, 3.0] | **1.5** [06, 3.4] | **1.8** [1.2, 2.6] | **1.9** [1.0, 3.4] | **0.9** [0.5, 2.0] | **1.5** [1.0, 2.6] |
| **p-values** | **p<0.01** | | **p<0.01** | p=0.10 | | p=0.58 | **p=0.01** | | **p=0.01** |
| **Monocytes** | **193.5** [87.0, 275.8] | **218.5** [132.3, 407.1] | **261.8** [156.2, 287.6] | **229.7** [149.6, 325.6] | **274.7** [181.9, 377.4] | **222.1** [164.7, 329.4] | **223.1** [124.6, 322.2] | **279.2** [179.1, 396.9] | **222.0** [103.8, 394.4] |
| **p-values** | **p=0.02** | | **p=0.05** | p=0.08 | | **p=0.03** | p=0.24 | | **p=0.02** |
| **Classical Monocytes** | **177.7** [80.5, 250.5] | **209.0** [126.1, 412.4] | **257.1** [143.5, 278.7] | **213.9** [128.4, 307.9] | **257.8** [161.3, 344.0] | **210.4** [141.8, 316.6] | **202.6** [116.5, 278.7] | **272.6** [169.3, 360.9] | **203.7** [91.3, 296.4] |
| **p-values** | **p=0.01** | | **p=0.04** | p=0.07 | | p=0.06 | p=0.19 | | **p=0.02** |
| **Non-Classical Monocytes** | **9.6** [3.1, 14.1] | **4.8** [2.7, 7.3] | **9.9** [4.2, 10.7] | **15.3** [6.9, 24.4] | **14.3** [6.6, 23.2] | **11.9** [8.3, 23.2] | **10.9** [7.8, 24.3] | **5.4** [2.7, 13.0] | **13.9** [7.4, 19.1] |
| **p-values** | **p<0.01** | | p=0.06 | p=0.82 | | p=0.98 | **p<0.01** | | **p=0.04** |
| **Granu** | **1154.6** | **3183.9** | **1744.8** | **1908.9** | **1822.9** | **1635.6** | **1697.5** | **2256** | **1410.6** |

| | | | | | | | | | |
|---|---|---|---|---|---|---|---|---|---|
| **locytes** | [586.0, 2103.7] | [2012.1, 5487.0] | [700.8, 2318.7] | [1065.8, 2577.6] | [1436.7, 3429.1] | [966.9, 2544.9] | [929.8, 2969.1] | **.2** [1537.9, 3765.8] | [799.2, 2213.3] |
| **p-values** | **p<0.01** | | **p<0.01** | **p<0.01** | | **p<0.01** | p=0.30 | | **p=0.01** |
| **Neutrophils** | **954.7** [527.2, 1849.6] | **3126.4** [1934.4, 4336.9] | **1646.1** [588.2, 2221.1] | **1677.1** [883.2, 2421.5] | **1739.2** [1322.1, 3181.4] | **1455.2** [859.0, 2491.6] | **1540.6** [629.2, 2703.5] | **1996.8** [1469.1, 3824.7] | **1117.8** [639.8, 1866.1] |
| **p-values** | **p<0.01** | | **p<0.01** | **p=0.01** | | **p=0.03** | p=0.25 | | **p=0.02** |
| **Eosinophils** | **26.1** [18.2, 82.6] | **0.9** [0.2, 2.8] | **37.5** [24.5, 67.6] | **32.8** [17.9, 64.5] | **29.2** [14.9, 53.2] | **45.9** [18.6, 76.8] | **64.3** [21.8, 93.7] | **13.8** [3.1, 49.4] | **35.9** [16.3, 92.3] |
| **p-values** | **p<0.01** | | **p<0.01** | **p=0.04** | | **p<0.01** | **p<0.01** | | **p=0.03** |

Data is depicted as absolute cell concentrations (n/µl) per time point and respective p-values regarding the changes between 24 hours pre versus 24 hours post, and 24 hours post versus 8 weeks post treatment are depicted in the row below. P-values below p=0.05 are bold printed. cTACE, conventional transarterial chemoembolization. iBT, interstitial brachytherapy. cDCs, conventional dendritic cells.

**Table S2:** Alterations of immune cell populations among the time points.

| | **From baseline to one day post-LRT** | **From one day post-LRT to two months post-LRT** |
|---|---|---|

| Relative Change (%) | Lower CI | Cohens D | Upper CI | p-value | Lower CI | Cohens D | Upper CI | p-value |
|---|---|---|---|---|---|---|---|---|
| **Lymphocytes** | -1.22 | **-0.99** | -0.78 | **p<0.01** | 0.68 | **0.91** | 1.15 | **p<0.01** |
| **Myeloid Cells** | 0.73 | **0.95** | 1.16 | **p<0.01** | -1.10 | **-0.87** | -0.63 | **p<0.01** |
| **T cells** | -0.28 | -0.08 | 0.13 | p=0.14 | -0.16 | 0.07 | 0.29 | p=0.20 |
| **$CD4^+$ T cells** | -0.38 | **-0.18** | 0.03 | **p<0.01** | -0.04 | **0.18** | 0.40 | **p<0.01** |
| **$CD8^+$ T cells** | -0.06 | **0.15** | 0.35 | **p=0.01** | -0.42 | **-0.19** | 0.03 | **p<0.01** |
| **$CD4^+CD8^+$ T cells** | -0.28 | -0.02 | 0.19 | p=0.46 | -0.26 | -0.04 | 0.19 | p=0.31 |
| **$CD4^-CD8^-$ T cells** | -0.11 | **0.10** | 0.31 | **p=0.02** | -0.31 | -0.08 | 0.14 | p=0.38 |
| **NK T cells** | -0.13 | 0.08 | 0.29 | p=0.22 | -0.28 | -0.05 | 0.17 | p=0.58 |
| **$CD4^+$ NK T cells** | -0.24 | -0.03 | 0.18 | p=0.98 | -0.17 | 0.06 | 0.28 | p=0.33 |
| **$CD8^+$ NK T cells** | -0.22 | -0.01 | 0.20 | p=0.70 | -0.34 | **-0.12** | 0.11 | **p<0.01** |
| **B cells** | -0.04 | **0.16** | 0.37 | **p<0.01** | -0.68 | **-0.45** | -0.22 | **p<0.01** |
| **Dendritic Cells (cDC)** | -0.84 | **-0.62** | -0.40 | **p<0.01** | 0.47 | **0.71** | 0.94 | **p<0.01** |
| **Type 1 cDCs** | -0.11 | **0.11** | 0.33 | p=0.13 | -0.28 | -0.05 | 0.19 | p=0.36 |
| **Type 2 cDCs** | -0.41 | **-0.19** | 0.03 | **p=0.03** | -0.10 | **0.13** | 0.36 | p=0.11 |
| **Monocytes** | -0.59 | **-0.38** | -0.18 | **p<0.01** | 0.15 | **0.37** | 0.60 | **p<0.01** |
| **Classical Monocytes** | 0.37 | **0.58** | 0.80 | **p<0.01** | -0.79 | **-0.56** | -0.32 | **p<0.01** |
| **Non-Classical Monocytes** | -0.83 | **-0.62** | -0.40 | **p<0.01** | 0.39 | **0.62** | 0.85 | **p<0.01** |
| **NK cells** | -0.13 | 0.08 | 0.29 | p=0.09 | -0.25 | -0.03 | 0.19 | p=0.59 |
| **$CD56^{bright}$ NK cells** | -0.60 | **-0.39** | -0.18 | **p<0.01** | 0.32 | **0.55** | 0.78 | **p<0.01** |
| **$CD56^{diminished}$ NK cells** | 0.19 | **0.40** | 0.61 | **p<0.01** | -0.85 | **-0.62** | -0.39 | **p<0.01** |
| **Granulocytes** | 0.42 | **0.63** | 0.84 | **p<0.01** | -0.82 | **-0.59** | -0.36 | **p<0.01** |

| | | | | | | | | |
|---|---|---|---|---|---|---|---|---|
| **Neutrophils** | 0.52 | **0.74** | 0.96 | **p<0.01** | -1.01 | **-0.77** | -0.54 | **p<0.01** |
| **Eosinophils** | -0.57 | **-0.36** | -0.15 | **p<0.01** | -0.01 | **0.22** | 0.44 | **p=0.01** |
| | | | | | | | | |
| **Absolute Change (n/µl)** | Lower CI | Cohens D | Upper CI | p-value | Lower CI | Cohens D | Upper CI | p-value |
| **Leucocytes** | 0.37 | **0.59** | 0.80 | **p<0.01** | -0.79 | **-0.56** | -0.33 | **p<0.01** |
| **Lymphocytes** | -0.69 | **-0.48** | -0.26 | **p<0.01** | 0.19 | **0.43** | 0.66 | **p<0.01** |
| **Myeloid Cells** | 0.51 | **0.73** | 0.95 | **p<0.01** | -0.91 | **-0.67** | -0.44 | **p<0.01** |
| **T cells** | -0.74 | **-0.53** | -0.31 | **p<0.01** | 0.29 | **0.53** | 0.76 | **p<0.01** |
| **$CD4^+$ T cells** | -0.77 | **-0.56** | -0.34 | **p<0.01** | 0.29 | **0.53** | 0.76 | **p<0.01** |
| **$CD8^+$ T cells** | -0.51 | **-0.29** | -0.08 | **p<0.01** | -0.01 | **0.22** | 0.45 | **p<0.01** |
| **$CD4^+CD8^+$ T cells** | -0.51 | **-0.29** | -0.08 | **p<0.01** | -0.04 | **0.19** | 0.41 | **p=0.01** |
| **$CD4^-CD8^-$ T cells** | -0.49 | **-0.28** | -0.07 | **p<0.01** | 0.05 | **0.28** | 0.51 | **p<0.01** |
| **NK T cells** | -0.38 | **-0.16** | 0.06 | **p<0.01** | -0.06 | **0.17** | 0.40 | **p=0.01** |
| **$CD4^+$ NK T cells** | -0.31 | -0.09 | 0.12 | p=0.09 | -0.06 | **0.17** | 0.40 | **p=0.04** |
| **$CD8^+$ NK T cells** | -0.32 | -0.09 | 0.12 | **p=0.02** | -0.17 | 0.06 | 0.29 | p=0.09 |
| **B cells** | -0.39 | **-0.18** | 0.03 | p=0.09 | -0.32 | -0.09 | 0.14 | p=0.14 |
| **Dendritic Cells (cDC)** | -0.70 | **-0.45** | -0.25 | **p<0.01** | 0.17 | **0.41** | 0.64 | **p<0.01** |
| **Type 1 cDCs** | -0.64 | **-0.42** | -0.19 | **p<0.01** | 0.14 | **0.38** | 0.61 | **p<0.01** |
| **Type 2 cDCs** | -0.62 | **-0.40** | -0.17 | **p<0.01** | 0.11 | **0.34** | 0.58 | **p<0.01** |
| **Monocytes** | 0.14 | **0.36** | 0.57 | **p<0.01** | -0.57 | **-0.34** | -0.11 | **p<0.01** |
| **Classical Monocytes** | 0.17 | **0.39** | 0.61 | **p<0.01** | -0.59 | **-0.36** | -0.12 | **p<0.01** |
| **Non-Classical Monocytes** | -0.42 | **-0.20** | 0.02 | **p<0.01** | -0.06 | **0.18** | 0.41 | p=0.07 |

| | | | | | | | | |
|---|---|---|---|---|---|---|---|---|
| **NK cells** | -0.52 | **-0.30** | -0.08 | **p<0.01** | 0.09 | **0.32** | 0.55 | **p<0.01** |
| **CD56$^{bright}$ NK cells** | -0.99 | **-0.77** | -0.54 | **p<0.01** | 0.74 | **0.98** | 1.22 | **p<0.01** |
| **CD56$^{diminshed}$ NK cells** | -0.47 | **-0.26** | -0.04 | **p<0.01** | 0.04 | **0.27** | 0.50 | **p<0.01** |
| **Granulocytes** | 0.53 | **0.75** | 0.97 | **p<0.01** | -0.98 | **-0.74** | -0.49 | **p<0.01** |
| **Neutrophils** | 0.54 | **0.77** | 0.99 | **p<0.01** | -0.98 | **-0.74** | -0.49 | **p<0.01** |
| **Eosinophils** | -0.45 | **-0.23** | -0.01 | p=0.11 | 0.05 | **0.28** | 0.52 | **p=0.05** |
| *Data is depicted as Cohens D and lower/upper confidence interval (CI). A Cohens D above/below ± 0.1 is bold printed. cDCs, conventional dendritic cells.* | | | | | | | | |

**Table S3:** Alterations of activation, exhaustion, and checkpoint molecules on all immune cell populations among the time points.

| | **From baseline to one day post-LRT** | | | | **From one day to two months post- LRT** | | | |
|---|---|---|---|---|---|---|---|---|
| **Relative Change (%)** | Lower CI | Cohens D | Upper CI | p-value | Lower CI | Cohens D | Upper CI | p-value |
| **CD4$^+$ T cells$_{PD1}$** | -0.20 | 0.01 | 0.22 | p=0.69 | -0.19 | 0.04 | 0.26 | p=0.49 |
| **CD8$^+$ T cells$_{PD1}$** | -0.21 | 0.01 | 0.21 | p=0.52 | -0.28 | -0.06 | 0.17 | p=0.90 |
| **CD4$^+$ CD8$^+$ T cells$_{PD1}$** | -0.20 | 0.01 | 0.22 | p=0.90 | -0.27 | -0.04 | 0.18 | p=0.72 |
| **CD4$^-$ CD8$^-$ T cells$_{PD1}$** | -0.16 | 0.05 | 0.25 | p=0.64 | -0.26 | -0.03 | 0.19 | p=0.95 |
| **CD4$^+$ NK T cells$_{PD1}$** | -0.18 | 0.03 | 0.24 | p=0.50 | -0.31 | -0.08 | 0.15 | p=0.22 |
| **CD8$^+$ NK T cells$_{PD1}$** | -0.20 | 0.01 | 0.22 | p=0.30 | -0.26 | -0.03 | 0.20 | p=0.49 |
| **CD4$^+$ T cells$_{CD38}$** | -0.13 | 0.08 | 0.28 | **p=0.05** | -0.32 | -0.09 | 0.13 | p=0.06 |
| **CD8$^+$ T cells$_{CD38}$** | -0.38 | **-0.18** | 0.03 | **p<0.01** | 0.02 | **0.24** | 0.47 | **p=0.03** |
| **CD4$^+$ CD8$^+$ T cells$_{CD38}$** | -0.09 | **0.12** | 0.33 | p=0.08 | -0.14 | 0.08 | 0.31 | p=0.29 |
| **CD4$^-$ CD8$^-$ T cells$_{CD38}$** | -0.36 | **-0.15** | 0.05 | **p<0.01** | -0.01 | **0.21** | 0.44 | p=0.13 |

| | | | | | | | | |
|---|---|---|---|---|---|---|---|---|
| **CD4⁺ NK T cells$_{CD38}$** | -0.31 | **-0.10** | 0.11 | p=0.40 | -0.23 | -0.01 | 0.22 | p=0.86 |
| **CD8⁺ NK T cells$_{CD38}$** | -0.36 | **-0.15** | 0.06 | **p=0.05** | 0.06 | **0.29** | 0.51 | **p=0.01** |
| **CD4⁺ T cells$_{PD-L1}$** | -0.17 | 0.04 | 0.25 | p=0.87 | -0.08 | **0.14** | 0.37 | p=0.27 |
| **CD8⁺ T cells$_{PD-L1}$** | -0.24 | -0.03 | 0.17 | p=0.15 | -0.26 | -0.04 | 0.19 | p=0.41 |
| **CD4⁺ CD8⁺ T cells$_{PD-L1}$** | -0.08 | **0.13** | 0.34 | p=0.09 | -0.25 | -0.03 | 0.20 | p=0.64 |
| **CD4⁻ CD8⁻ T cells$_{PD-L1}$** | -0.30 | -0.09 | 0.12 | p=0.59 | -0.20 | 0.03 | 0.25 | p=0.77 |
| **CD4⁺ NK T cells$_{PD-L1}$** | -0.32 | **-0.11** | 0.10 | p=0.44 | -0.13 | **0.10** | 0.32 | p=0.83 |
| **CD8⁺ NK T cells$_{PD-L1}$** | -0.17 | 0.04 | 0.24 | p=0.34 | -0.20 | 0.02 | 0.24 | p=0.26 |
| **CD4⁺ T cells$_{CTLA-4}$** | 0.33 | **0.54** | 0.75 | **p<0.01** | -0.65 | **-0.42** | -0.20 | **p<0.01** |
| **CD8⁺ T cells$_{CTLA-4}$** | 0.36 | **0.57** | 0.78 | **p<0.01** | -0.35 | **-0.13** | 0.10 | p=0.26 |
| **CD4⁺ CD8⁺ T cells$_{CTLA-4}$** | -0.10 | **0.10** | 0.31 | p=0.34 | -0.59 | **-0.36** | -0.14 | **p<0.01** |
| **CD4⁻ CD8⁻ T cells$_{CTLA-4}$** | 0.14 | **0.35** | 0.56 | **p<0.01** | -0.56 | **-0.33** | -0.11 | **p<0.01** |
| **CD4⁺ NK T cells$_{CTLA-4}$** | -0.15 | 0.06 | 0.26 | p=0.55 | -0.11 | **0.11** | 0.34 | p=0.53 |
| **CD8⁺ NK T cells$_{CTLA-4}$** | 0.02 | **0.23** | 0.43 | p=0.16 | -0.20 | 0.03 | 0.25 | p=0.08 |
| **CD4⁺ T cells$_{HLA-DR}$** | -0.27 | -0.06 | 0.14 | p=0.73 | -0.05 | **0.18** | 0.40 | p=0.08 |
| **CD8⁺ T cells$_{HLA-DR}$** | -0.38 | **-0.17** | 0.03 | p=0.08 | 0.25 | **0.47** | 0.70 | **p<0.01** |
| **CD4⁺ CD8⁺ T cells$_{HLA-DR}$** | -0.28 | -0.07 | 0.14 | p=0.07 | -0.10 | **0.13** | 0.35 | **p<0.01** |
| **CD4⁻ CD8⁻ T cells$_{HLA-DR}$** | -0.39 | **-0.18** | 0.03 | **p=0.02** | 0.17 | **0.39** | 0.62 | **p<0.01** |
| **CD4⁺ NK T cells$_{HLA-DR}$** | -0.20 | 0.01 | 0.22 | p=0.86 | -0.03 | **0.19** | 0.42 | p=0.08 |
| **CD8⁺ NK T cells$_{HLA-DR}$** | -0.35 | **-0.14** | 0.07 | p=0.12 | 0.12 | **0.35** | 0.57 | **p=0.01** |
| **CD4⁺ T cells$_{CD161}$** | -0.37 | **-0.15** | 0.07 | p=0.07 | 0.14 | **0.38** | 0.61 | **p<0.01** |
| **CD8⁺ T cells$_{CD161}$** | -0.36 | **-0.15** | 0.07 | **p<0.01** | -0.13 | **0.11** | 0.34 | **p<0.01** |
| **CD4⁺ CD8⁺ T cells$_{CD161}$** | -0.21 | 0.01 | 0.23 | p=0.76 | -0.27 | -0.04 | 0.20 | p=0.16 |
| **CD4⁻ CD8⁻ T cells$_{CD161}$** | -0.32 | **-0.10** | 0.13 | p=0.31 | -0.19 | 0.05 | 0.29 | p=0.20 |
| **CD4⁺ NK T cells$_{CD161}$** | -0.40 | **-0.18** | 0.04 | p=0.14 | -0.13 | **0.11** | 0.35 | p=0.51 |
| **CD8⁺ NK T cells$_{CD161}$** | 0.07 | **0.29** | 0.50 | **p<0.01** | -0.70 | **-0.46** | -0.23 | **p=0.02** |

| | | | | | | | | |
|---|---|---|---|---|---|---|---|---|
| **CD4$^{+}$ T cells$_{TIM3}$** | 0.07 | **0.29** | 0.50 | **p=0.03** | -0.69 | **-0.46** | -0.23 | **p<0.01** |
| **CD8$^{+}$ T cells$_{TIM3}$** | -0.17 | 0.04 | 0.26 | p=0.38 | -0.22 | 0.01 | 0.25 | p=0.81 |
| **CD4$^{+}$ CD8$^{+}$ T cells$_{TIM3}$** | -0.27 | -0.06 | 0.15 | p=0.85 | 0.05 | **0.29** | 0.52 | **p=0.01** |
| **CD4$^{-}$ CD8$^{-}$ T cells$_{TIM3}$** | -0.22 | -0.01 | 0.21 | p=0.87 | -0.40 | **-0.17** | 0.07 | **p<0.01** |
| **CD4$^{+}$ NK T cells$_{TIM3}$** | -0.28 | -0.07 | 0.15 | p=0.85 | 0.02 | **0.26** | 0.50 | p=0.13 |
| **CD8$^{+}$ NK T cells$_{TIM3}$** | -0.35 | **-0.14** | 0.08 | **p=0.04** | -0.11 | **0.13** | 0.36 | **p=0.04** |
| **CD4$^{+}$ T cells$_{LAG3}$** | -0.18 | 0.04 | 0.26 | p=0.26 | -0.57 | **-0.34** | -0.11 | **p=0.01** |
| **CD8$^{+}$ T cells$_{LAG3}$** | -0.18 | 0.03 | 0.25 | p=0.19 | -0.45 | **-0.21** | 0.02 | p=0.08 |
| **CD4$^{+}$ CD8$^{+}$ T cells$_{LAG3}$** | -0.27 | -0.06 | 0.16 | p=0.85 | 0.04 | **0.28** | 0.51 | **p=0.01** |
| **CD4$^{-}$ CD8$^{-}$ T cells$_{LAG3}$** | -0.21 | 0.01 | 0.21 | p=0.83 | -0.41 | **-0.18** | 0.06 | **p=0.02** |
| **CD4$^{+}$ NK T cells$_{LAG3}$** | -0.23 | -0.01 | 0.21 | p=0.30 | -0.49 | **-0.27** | -0.03 | **p=0.03** |
| **CD8$^{+}$ NK T cells$_{LAG3}$** | -0.18 | 0.05 | 0.27 | p=0.24 | -0.57 | **-0.34** | -0.10 | **p=0.01** |
| **B cells$_{CD11b}$** | -0.09 | **0.12** | 0.33 | p=0.76 | -0.19 | 0.04 | 0.26 | p=0.58 |
| **B cells$_{CD19high}$** | -0.23 | **-0.27** | 0.18 | p=0.93 | -0.09 | **0.13** | 0.36 | p=0.13 |
| **B cells$_{CD38high}$** | -0.21 | -0.01 | 0.20 | p=0.33 | -0.58 | **-0.36** | -0.13 | **p<0.01** |
| **B cells$_{CD38int}$** | -0.23 | -0.03 | 0.18 | p=0.90 | -0.09 | **0.13** | 0.36 | **p<0.01** |
| **B cells$_{CD38low}$** | -0.13 | 0.09 | 0.30 | p=0.58 | -0.04 | **0.19** | 0.42 | **p=0.02** |
| **B cells$_{PD-L1}$** | -0.12 | 0.09 | 0.30 | p=0.67 | -0.29 | -0.06 | 0.17 | **p=0.04** |
| **Classical Monocytes$_{CD1c}$** | -0.11 | **0.10** | 0.32 | p=0.10 | -0.14 | 0.09 | 0.32 | p=0.09 |
| **Non-Classical Monocytes$_{PD-L1}$** | -0.44 | **-0.22** | -0.01 | p=0.64 | 0.07 | **0.30** | 0.53 | p=0.13 |
| **Classical Monocytes$_{CTLA-4}$** | -0.20 | 0.01 | 0.22 | p=0.64 | -0.39 | **-0.16** | 0.07 | p=0.62 |
| **Non-Classical Monocytes$_{CTLA-4}$** | -0.28 | -0.06 | 0.15 | p=0.08 | -0.16 | 0.07 | 0.30 | p=0.18 |
| **Classical** | -0.41 | **-0.20** | 0.02 | **p=0.05** | 0.36 | **0.59** | 0.82 | **p<0.01** |

| | | | | | | | | |
|---|---|---|---|---|---|---|---|---|
| **Monocytes$_{HLA-DR}$** | | | | | | | | |
| **Non-Classical Monocytes$_{HLA-DR}$** | -0.19 | 0.02 | 0.23 | p=0.85 | 0.11 | **0.34** | 0.57 | **p<0.01** |
| **Classical Monocytes$_{TIM3}$** | -0.41 | **-0.19** | 0.03 | **p=0.01** | -0.26 | -0.02 | 0.22 | p=0.55 |
| **Non-Classical Monocytes$_{TIM3}$** | -0.60 | **-0.38** | -0.16 | **p<0.01** | 0.01 | **0.24** | 0.48 | **p<0.01** |
| **Classical Monocytes$_{CD56}$** | 0.19 | **0.40** | 0.62 | **p<0.01** | -0.62 | **-0.39** | -0.16 | **p<0.01** |
| **Non-Classical Monocytes$_{CD56}$** | -0.25 | -0.04 | 0.18 | p=0.38 | 0.04 | **0.27** | 0.50 | p=0.04 |
| **NK cells$_{PD-1}$** | -0.20 | 0.01 | 0.22 | p=0.98 | -0.14 | 0.09 | 0.31 | p=0.58 |
| **NK cells$_{HLA-DR}$** | -0.25 | -0.05 | 0.16 | **p=0.05** | 0.04 | **0.27** | 0.50 | p=0.46 |
| **NK cells$_{CD161}$** | -0.28 | -0.06 | 0.16 | **p=0.01** | -0.19 | 0.04 | 0.28 | p=0.07 |
| **NK cells$_{CD8}$** | -0.23 | -0.02 | 0.19 | p=0.73 | -0.39 | **-0.17** | 0.06 | **p=0.01** |
| **Eosinophils$_{HLA-DR}$** | -0.25 | -0.03 | 0.18 | p=0.54 | -0.05 | **0.18** | 0.41 | p=0.28 |
| | | | | | | | | |
| **Absolute Change (%)** | Lower CI | Cohens D | Upper CI | p-value | Lower CI | Cohens D | Upper CI | p-value |
| **CD4$^{+}$ T cells$_{PD1}$** | -0.70 | **-0.49** | -0.27 | **p<0.01** | 0.28 | **0.51** | 0.74 | **p<0.01** |
| **CD8$^{+}$ T cells$_{PD1}$** | -0.45 | **-0.23** | -0.02 | **p<0.01** | -0.02 | **0.21** | 0.44 | **p<0.01** |
| **CD4$^{+}$ CD8$^{+}$ T cells$_{PD1}$** | -0.46 | **-0.24** | -0.03 | **p=0.01** | -0.07 | **0.16** | 0.39 | p=0.09 |
| **CD4$^{-}$ CD8$^{-}$ T cells$_{PD1}$** | -0.45 | **-0.24** | -0.02 | **p<0.01** | 0.08 | **0.31** | 0.54 | **p<0.01** |
| **CD4$^{+}$ NK T cells$_{PD1}$** | -0.28 | -0.07 | 0.15 | p=0.29 | -0.10 | **0.16** | 0.36 | p=0.44 |
| **CD8$^{+}$ NK T cells$_{PD1}$** | -0.28 | -0.06 | 0.15 | **p<0.01** | -0.19 | 0.04 | 0.27 | **p=0.01** |
| **CD4$^{+}$ T cells$_{CD38}$** | -0.59 | **-0.37** | -0.15 | **p<0.01** | 0.10 | **0.33** | 0.56 | **p<0.01** |
| **CD8$^{+}$ T cells$_{CD38}$** | -0.54 | **-0.32** | -0.10 | **p<0.01** | 0.09 | **0.32** | 0.54 | **p<0.01** |

| | | | | | | | | |
|---|---|---|---|---|---|---|---|---|
| $CD4^{+}$ $CD8^{+}$ T cells$_{CD38}$ | -0.37 | **-0.15** | 0.06 | p=0.08 | -0.07 | **0.16** | 0.39 | p=0.29 |
| $CD4^{-}$ $CD8^{-}$ T cells$_{CD38}$ | -0.48 | **-0.26** | -0.05 | **p<0.01** | 0.04 | **0.27** | 0.50 | **p<0.01** |
| $CD4^{+}$ NK T cells$_{CD38}$ | -0.29 | -0.08 | 0.14 | p=0.26 | -0.12 | **0.11** | 0.34 | p=0.49 |
| $CD8^{+}$ NK T cells$_{CD38}$ | -0.36 | **-0.14** | 0.07 | **p=0.02** | -0.07 | **0.16** | 0.40 | p=0.15 |
| $CD4^{+}$ T cells$_{PD-L1}$ | -0.27 | -0.05 | 0.17 | **p=0.03** | -0.08 | **0.15** | 0.34 | p=0.25 |
| $CD8^{+}$ T cells$_{PD-L1}$ | -0.40 | **-0.18** | 0.03 | p=0.20 | -0.06 | **0.17** | 0.40 | p=0.34 |
| $CD4^{+}$ $CD8^{+}$ T cells$_{PD-L1}$ | -0.17 | 0.04 | 0.26 | p=0.81 | -0.13 | **0.10** | 0.33 | p=0.29 |
| $CD4^{-}$ $CD8^{-}$ T cells$_{PD-L1}$ | -0.54 | **-0.32** | -0.10 | **p=0.05** | -0.02 | **0.21** | 0.44 | p=0.15 |
| $CD4^{+}$ NK T cells$_{PD-L1}$ | -0.38 | **-0.16** | 0.06 | **p=0.02** | -0.20 | 0.03 | 0.26 | p=0.13 |
| $CD8^{+}$ NK T cells$_{PD-L1}$ | -0.29 | -0.07 | 0.14 | **p=0.04** | -0.25 | -0.02 | 0.21 | p=0.20 |
| $CD4^{+}$ T cells$_{CTLA-4}$ | 0.01 | **0.23** | 0.44 | **p<0.01** | -0.35 | **-0.12** | 0.11 | **p=0.13** |
| $CD8^{+}$ T cells$_{CTLA-4}$ | 0.31 | **0.53** | 0.75 | **p<0.01** | -0.62 | **-0.39** | -0.16 | **p<0.01** |
| $CD4^{+}$ $CD8^{+}$ T cells$_{CTLA-4}$ | 0.06 | **0.28** | 0.50 | **p<0.01** | -0.71 | **-0.48** | -0.24 | **p<0.01** |
| $CD4^{-}$ $CD8^{-}$ T cells$_{CTLA-4}$ | 0.12 | **0.33** | 0.55 | **p<0.01** | -0.42 | **-0.19** | 0.04 | **p<0.01** |
| $CD4^{+}$ NK T cells$_{CTLA-4}$ | -0.21 | 0.01 | 0.22 | p=0.08 | -0.10 | **0.13** | 0.36 | p=0.27 |
| $CD8^{+}$ NK T cells$_{CTLA-4}$ | -0.11 | **0.11** | 0.32 | p=0.28 | -0.16 | 0.07 | 0.30 | p=0.59 |
| $CD4^{+}$ T cells$_{HLA-DR}$ | -0.78 | **-0.57** | -0.35 | **p<0.01** | 0.30 | **0.53** | 0.77 | **p<0.01** |
| $CD8^{+}$ T cells$_{HLA-DR}$ | -0.58 | **-0.36** | -0.15 | **p<0.01** | 0.28 | **0.52** | 0.75 | **p<0.01** |
| $CD4^{+}$ $CD8^{+}$ T cells$_{HLA-DR}$ | -0.45 | **-0.24** | -0.02 | **p<0.01** | -0.01 | **0.23** | 0.45 | **p<0.01** |
| $CD4^{-}$ $CD8^{-}$ T cells$_{HLA-DR}$ | -0.52 | **-0.30** | -0.09 | **p<0.01** | 0.22 | **0.45** | 0.68 | **p<0.01** |
| $CD4^{+}$ NK T cells$_{HLA-DR}$ | -0.39 | **-0.17** | 0.05 | **p=0.02** | -0.02 | **0.21** | 0.44 | p=0.08 |
| $CD8^{+}$ NK T cells$_{HLA-DR}$ | -0.35 | **-0.14** | 0.08 | **p=0.01** | -0.03 | **0.20** | 0.43 | p=0.26 |
| $CD4^{+}$ T cells$_{CD161}$ | -0.72 | **-0.50** | -0.28 | **p<0.01** | 0.34 | **0.58** | 0.82 | **p<0.01** |
| $CD8^{+}$ T cells$_{CD161}$ | -0.49 | **-0.27** | -0.05 | **p<0.01** | -0.01 | **0.23** | 0.47 | **p<0.01** |
| $CD4^{+}$ $CD8^{+}$ T cells$_{CD161}$ | -0.46 | **-0.24** | -0.02 | **p<0.01** | 0.01 | **0.25** | 0.49 | **p<0.01** |
| $CD4^{-}$ $CD8^{-}$ T cells$_{CD161}$ | -0.35 | **-0.13** | 0.09 | **p<0.01** | -0.03 | **0.21** | 0.45 | **p<0.01** |

| | | | | | | | | |
|---|---|---|---|---|---|---|---|---|
| **$CD4^+$ NK T cells$_{CD161}$** | -0.43 | **-0.20** | 0.02 | **p=0.05** | -0.11 | **0.13** | 0.37 | p=0.16 |
| **$CD8^+$ NK T cells$_{CD161}$** | -0.13 | 0.09 | 0.31 | **p<0.01** | -0.51 | **-0.28** | -0.04 | p=0.16 |
| **$CD4^+$ T cells$_{TIM3}$** | -0.13 | 0.09 | 0.31 | p=0.40 | -0.51 | **-0.28** | -0.04 | **p=0.01** |
| **$CD8^+$ T cells$_{TIM3}$** | -0.29 | -0.08 | 0.13 | p=0.23 | -0.24 | -0.06 | 0.23 | p=0.89 |
| **$CD4^+$ $CD8^+$ T cells$_{TIM3}$** | -0.39 | **-0.18** | 0.04 | p=0.22 | 0.09 | **0.33** | 0.57 | **p=0.02** |
| **$CD4^-$ $CD8^-$ T cells$_{TIM3}$** | -0.54 | **-0.33** | -0.11 | **p<0.01** | -0.10 | **0.13** | 0.37 | **p=0.04** |
| **$CD4^+$ NK T cells$_{TIM3}$** | -0.16 | 0.06 | 0.28 | p=0.42 | -0.10 | **0.13** | 0.37 | p=0.60 |
| **$CD8^+$ NK T cells$_{TIM3}$** | -0.40 | **-0.19** | 0.03 | **p=0.01** | -0.03 | **0.21** | 0.45 | p=0.08 |
| **$CD4^+$ T cells$_{LAG3}$** | -0.21 | 0.01 | 0.23 | p=0.59 | -0.48 | **-0.26** | -0.02 | **p=0.03** |
| **$CD8^+$ T cells$_{LAG3}$** | -0.18 | 0.04 | 0.26 | p=0.28 | -0.47 | **-0.24** | -0.07 | **p=0.04** |
| **$CD4^+$ $CD8^+$ T cells$_{LAG3}$** | -0.39 | **-0.18** | 0.04 | **p=0.03** | 0.09 | **0.33** | 0.57 | **p<0.01** |
| **$CD4^-$ $CD8^-$ T cells$_{LAG3}$** | -0.55 | **-0.33** | -0.11 | **p<0.01** | -0.09 | **0.14** | 0.37 | p=0.07 |
| **$CD4^+$ NK T cells$_{LAG3}$** | -0.16 | 0.05 | 0.27 | p=0.27 | -0.32 | -0.09 | 0.14 | p=0.33 |
| **$CD8^+$ NK T cells$_{LAG3}$** | -0.14 | 0.08 | 0.29 | p=0.41 | -0.42 | **-0.19** | 0.05 | p=0.08 |
| **B cells$_{CD11b}$** | -0.26 | -0.04 | 0.17 | p=0.19 | -0.18 | 0.05 | 0.28 | p=0.26 |
| **B cells$_{CD19high}$** | -0.46 | **-0.25** | -0.04 | p=0.42 | -0.17 | -0.06 | 0.29 | p=0.40 |
| **B cells$_{CD38high}$** | -0.43 | **-0.21** | 0.01 | **p<0.01** | 0.01 | **0.24** | 0.47 | p=0.18 |
| **B cells$_{CD38int}$** | -0.41 | **-0.20** | 0.11 | **p=0.05** | -0.39 | -0.08 | 0.15 | **p=0.01** |
| **B cells$_{CD38low}$** | -0.22 | 0.01 | 0.22 | p=0.18 | -0.013 | **0.22** | 0.45 | p=0.16 |
| **B cells$_{PD-L1}$** | -0.42 | **-0.21** | 0.01 | p=0.11 | 0.01 | **0.23** | 0.48 | p=0.15 |
| **Classical Monocytes$_{CD1c}$** | 0.14 | **0.36** | 0.58 | **p<0.01** | -0.46 | **-0.23** | 0.01 | **p=0.01** |
| **Non-Classical Monocytes$_{CD1c}$** | -0.22 | 0.01 | 0.22 | **p=0.01** | -0.13 | **0.22** | 0.45 | **p=0.01** |
| **Non-Classical Monocytes$_{PD-L1}$** | -0.53 | **-0.31** | -0.09 | **p<0.01** | 0.11 | **0.35** | 0.58 | **p<0.01** |

| | | | | | | | | |
|---|---|---|---|---|---|---|---|---|
| **Classical Monocytes**CTLA-4 | 0.08 | **0.30** | 0.52 | p=0.78 | -0.52 | **-0.28** | -0.05 | p=0.68 |
| **Non-Classical Monocytes**CTLA-4 | -0.31 | -0.08 | 0.14 | **p<0.01** | -0.25 | 0.01 | 0.22 | p=0.20 |
| **Classical Monocytes**HLA-DR | 0.03 | **0.25** | 0.47 | p=0.74 | -0.23 | 0.01 | 0.24 | p=0.76 |
| **Non-Classical Monocytes**HLA-DR | -0.27 | -0.05 | 0.17 | **p<0.01** | -0.07 | **0.16** | 0.39 | **p<0.01** |
| **Classical Monocytes**TIM3 | -0.03 | **0.19** | 0.41 | p=0.60 | -0.50 | **-0.26** | -0.03 | p=0.37 |
| **Non-Classical Monocytes**TIM3 | -0.63 | **-0.41** | 0.19 | **p<0.01** | -0.04 | **0.28** | 0.52 | **p<0.01** |
| **Classical Monocytes**CD56 | 0.22 | **0.44** | 0.67 | **p<0.01** | -0.71 | **-0.48** | -0.24 | **p<0.01** |
| **Non-Classical Monocytes**CD56 | -0.42 | **-0.19** | 0.03 | p=0.15 | 0.15 | **0.38** | 0.62 | p=0.79 |
| **NK cells**PD-1 | -0.33 | **-0.11** | 0.10 | p=0.11 | 0.02 | **0.23** | 0.46 | **p<0.01** |
| **NK cells**HLA-DR | -0.52 | **-0.30** | -0.09 | p=0.23 | 0.33 | **0.57** | 0.79 | **p<0.01** |
| **NK cells**CD161 | -0.52 | **-0.31** | -0.09 | **p<0.01** | 0.12 | **0.36** | 0.60 | **p<0.01** |
| **NK cells**CD8 | -0.50 | **-0.28** | -0.07 | **p<0.01** | 0.01 | **0.24** | 0.47 | **p<0.01** |
| **Eosinophils**HLA-DR | -0.61 | **-0.39** | -0.16 | **p=0.02** | 0.25 | **0.48** | 0.72 | **p=0.04** |

*Data is depicted as Cohens D and lower/upper confidence interval (CI). A Cohens D above/below ± 0.1 is bold printed. PD1, programmed cell death protein 1 (CD279), PD-L1, programmed cell death protein 1 ligand 1 (CD274). CTLA-4, cytotoxic T-lymphocyte-associated Protein 4 (CD152). HLA-DR, human leucocyte antigen DR. TIM3, T-cell immunoglobulin and mucin-domain containing-3 (CD366). LAG3, lymphocyte-activation gene 3 (CD223).*

**Table S4.** Alterations of cytokines and chemokines from baseline to one day post-cTACE.

| Variable | Lower CI | Cohen's D | Upper CI | p-value |
|---|---|---|---|---|
| IL-1β | -0.24 | 0.29 | 0.81 | <0.01 |
| IL-2 | -0.40 | 0.12 | 0.64 | 0.29 |
| IL-4 | -0.62 | -0.10 | 0.42 | 0.94 |
| IL-5 | -0.62 | -0.10 | 0.42 | 0.34 |
| IL-6 | -0.11 | 0.41 | 0.94 | 0.96 |
| IL-8 | -1.05 | -0.53 | 0.00 | 0.94 |
| IL-10 | -0.36 | 0.16 | 0.68 | 0.31 |
| IL-13 | -0.55 | -0.04 | 0.48 | 0.03 |
| IL-17 | -0.98 | -0.46 | 0.07 | 0.01 |
| MIP1α | -0.67 | -0.16 | 0.37 | 0.01 |
| MCP1 | -1.07 | -0.54 | -0.01 | <0.01 |
| VEGF | -0.67 | -0.15 | 0.37 | <0.01 |
| INFγ | -1.27 | -0.73 | -0.19 | 0.03 |
| TNFα | -0.80 | -0.28 | 0.25 | 0.11 |
| βFGF | -0.45 | 0.07 | 0.59 | 0.73 |

**Table S5.** Tumor response after brachytherapy (iBT), conventional transarterial chemoembolization (cTACE), and cTACE/iBT according to response evaluation criteria in solid tumors (RECIST), modified (m)RECIST, and Liver Imaging and Data Reporting System – Treatment Response Algorithm version 2017 (LI-TRA v2017).

| | 1st Follow-Up (2 months post-LRT) | | | 2nd Follow-Up (6 months post-LRT) | | | 3rd Follow-Up (12 months post-LRT) | | |
|---|---|---|---|---|---|---|---|---|---|
| **RECIST** | **cTACE** n = 20 | **iBT** n = 58 | **cTACE/iBT** n = 37 | **cTACE** n = 14 | **iBT** n = 55 | **cTACE/iBT** n = 30 | **cTACE** n = 4 | **iBT** n = 47 | **cTACE/iBT** n = 32 |
| **CR** | - | - | - | - | - | - | - | - | - |
| **PR** | 30.0 % (6) | 20.7 % (12) | 29.7 % (11) | 42.9 % (6) | 32.7 % (18) | 53.3 % (16) | 50.0 % (2) | 46.8 % (22) | 75.0 % (24) |
| **SD** | 70.0 % (14) | 74.1 % (43) | 64.9 % (24) | 50.0 % (7) | 61.8 % (34) | 40.0 % (12) | 50.0 % (2) | 51.1 % (24) | 25.0 % (8) |
| **PD** | - | 5.2 % (3) | 5.4 % (2) | 7.1 % (1) | 5.5 % (3) | 6.7 % (2) | - | 2.1 % (1) | - |
| | | | | | | | | | |
| **mRECIST** | **cTACE** n = 20 | **iBT** n = 57 | **cTACE/iBT** n = 36 | **cTACE** n = 14 | **iBT** n = 54 | **cTACE/iBT** n = 29 | **cTACE** n = 4 | **iBT** n = 46 | **cTACE/iBT** n = 31 |
| **CR** | 55.0 % (11) | 66.7 % (38) | 63.9 % (23) | 50.0 % (7) | 68.5 % (37) | 65.5 % (19) | 50.0 % (2) | 71.7 % (33) | 74.2 % (23) |
| **PR** | 35.0 % | 15.8 | 25.0 % (9) | 21.4 % | 11.1 | 23.1 % (7) | - | 6.5 % | 12.9 % (4) |

| | | | | | | | | | |
|---|---|---|---|---|---|---|---|---|---|
| | (7) | % (9) | | (3) | % (6) | | | (3) | |
| **SD** | 5.0 % (2) | 17.5 % (10) | 8.3 % (3) | 14.3 % (2) | 16.7 % (9) | 6.9 % (2) | 25.0 % (1) | 13.0% (6) | 9.7 % (3) |
| **PD** | - | - | 2.8 % (1) | 14.3 % (2) | 3.7 % (2) | 3.5% (1) | 25.0 % (1) | 8.7 % (4) | 3.2 % (1) |
| | | | | | | | | | |
| **LI-TRA v2017** | **cTACE** n = 22 | **iBT** n = 62 | **cTACE/iBT** n = 39 | **cTACE** n = 13 | **iBT** n = 56 | **cTACE/iBT** n = 32 | **cTACE** n = 8 | **iBT** n = 58 | **cTACE/iBT** n = 28 |
| **Non-Viable** | 31.8 % (7) | 33.9 % (21) | 28.2 % (11) | 38.5 % (5) | 39.3 % (22) | 28.1 % (9) | 16.7 % (1) | 55.1 % (27) | 32.1 % (9) |
| **Equivocal** | 18.2 % (4) | 38.7 % (24) | 43.6 % (17) | 7.7 % (1) | 26.8 % (15) | 31.2 % (10) | 16.7 % (1) | 14.3 % (7) | 28.6 % (8) |
| **Viable** | 50.0 % (11) | 27.4 % (17) | 28.2 % (11) | 53.8 % (7) | 33.9 % (19) | 40.6 % (13) | 66.7 % (4) | 30.6 % (24) | 39.3 % (11) |

CR complete response; PR partial response; SD stable disease; PD progressive disease; LRT locoregional therapy.

Postinterventional follow-up times: 1st Follow-Up: 2 months, 2nd Follow-Up: 6 months, 3rd Follow-Up: 12 months.

**Table S6.** Immune alterations from baseline to one day post-locoregional therapy (LRT) as well as treatment response according to LIRADS Treatment Response Algorithm (LI-TRA) v2017 according to immune clustering including PD1$^{+}$, PDL-1$^{+}$ and CTLA4$^{+}$ CD4$^{+}$ and CD8$^{+}$ T cells as revealed by TSNE- analysis.

| **Immune cell population** | **Cluster 1 (n=68)** Cohen's D (95% CI) | **Cluster 2 (n=39)** Cohen's D (95% CI) |
|---|---|---|
| B cells | -0.45 [-0.61,-0.30] | 0.14 [-0.10,0.47] |
| T cells | -0.43 [-0.56, -0.33] | 0.07 [0.10,0.24] |
| NK cells | -0.37 [-0.62, -0.15] | -0.12 [-0.30, 0.15] |
| Monocytes | -0.12 [-0.43, 0.40] | 1.05 [0.59, 2.67] |
| Granulocytes | 0.24 [-0,34, 0.90] | 2.45 [0.81, 4.73] |
| cDCs | -0,52 [-0.80, -0.10] | 0.18 [-0.34, 0.97] |
| **LI-TRA v2017** 6 months post-LRT | | |
| Non-viable | 15 (30.6%) | 12 (36.4%) |
| Equivocal | 7 (14.3%) | 12 (36.4%) |
| Viable | 27 (55.1%) | 9 (27.3%) |

## Supplementary figures

Fig. S1

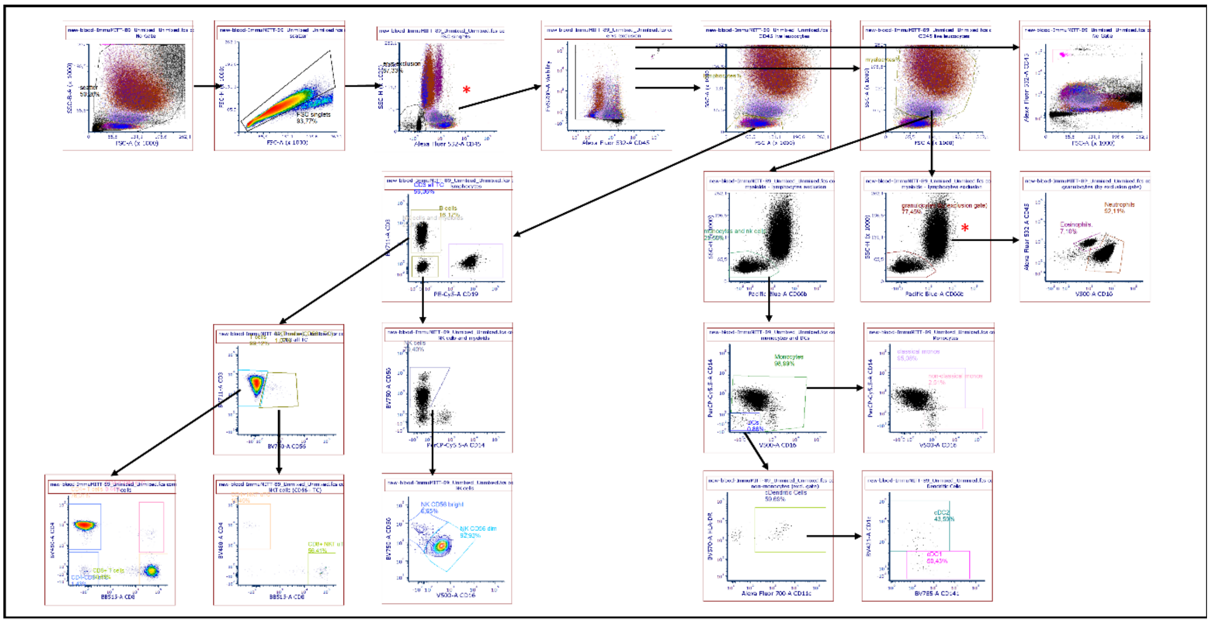

Fig. S2

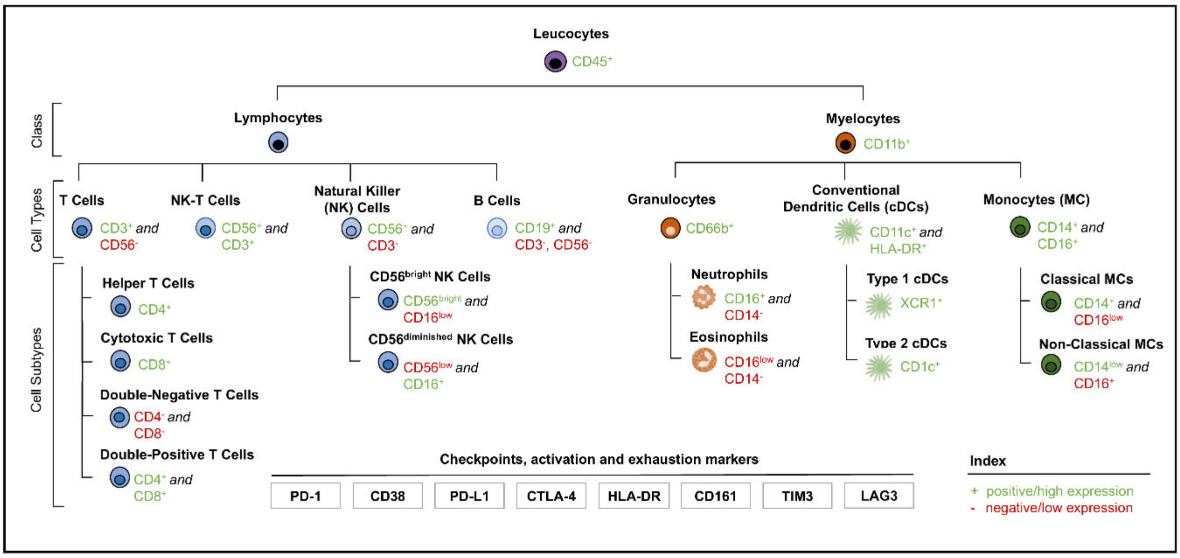


Fig. S3

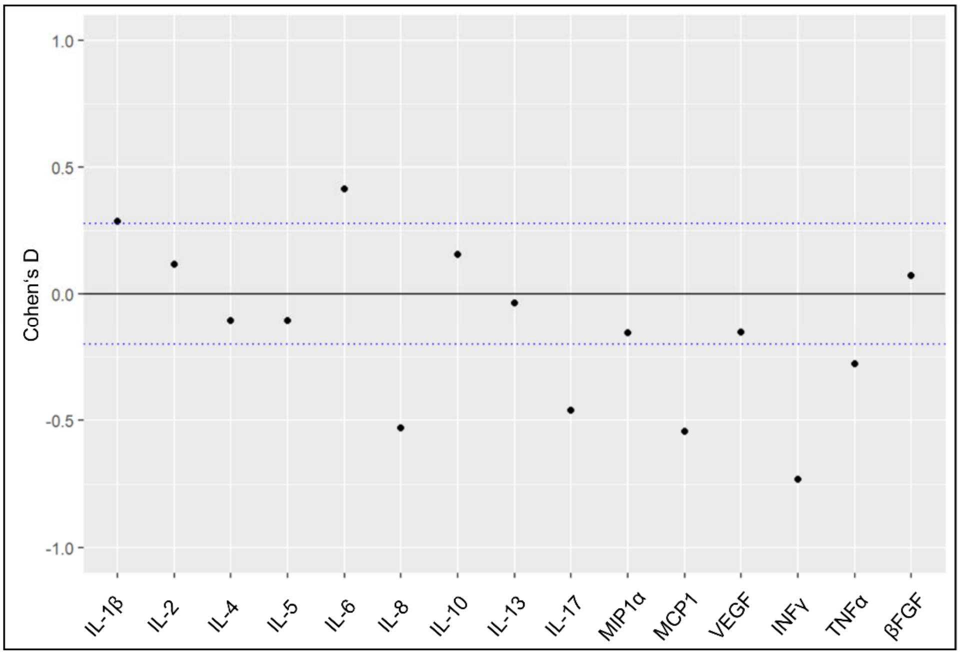
1.0
0.5
0.0
-0.5
-1.0
Cohen's D
IL-1β
IL-2
IL-4
IL-5
IL-6
IL-8
IL-10
IL-13
IL-17
MIP1α
MCP1
VEGF
INFγ
TNFα
βFGF

Fig. S4

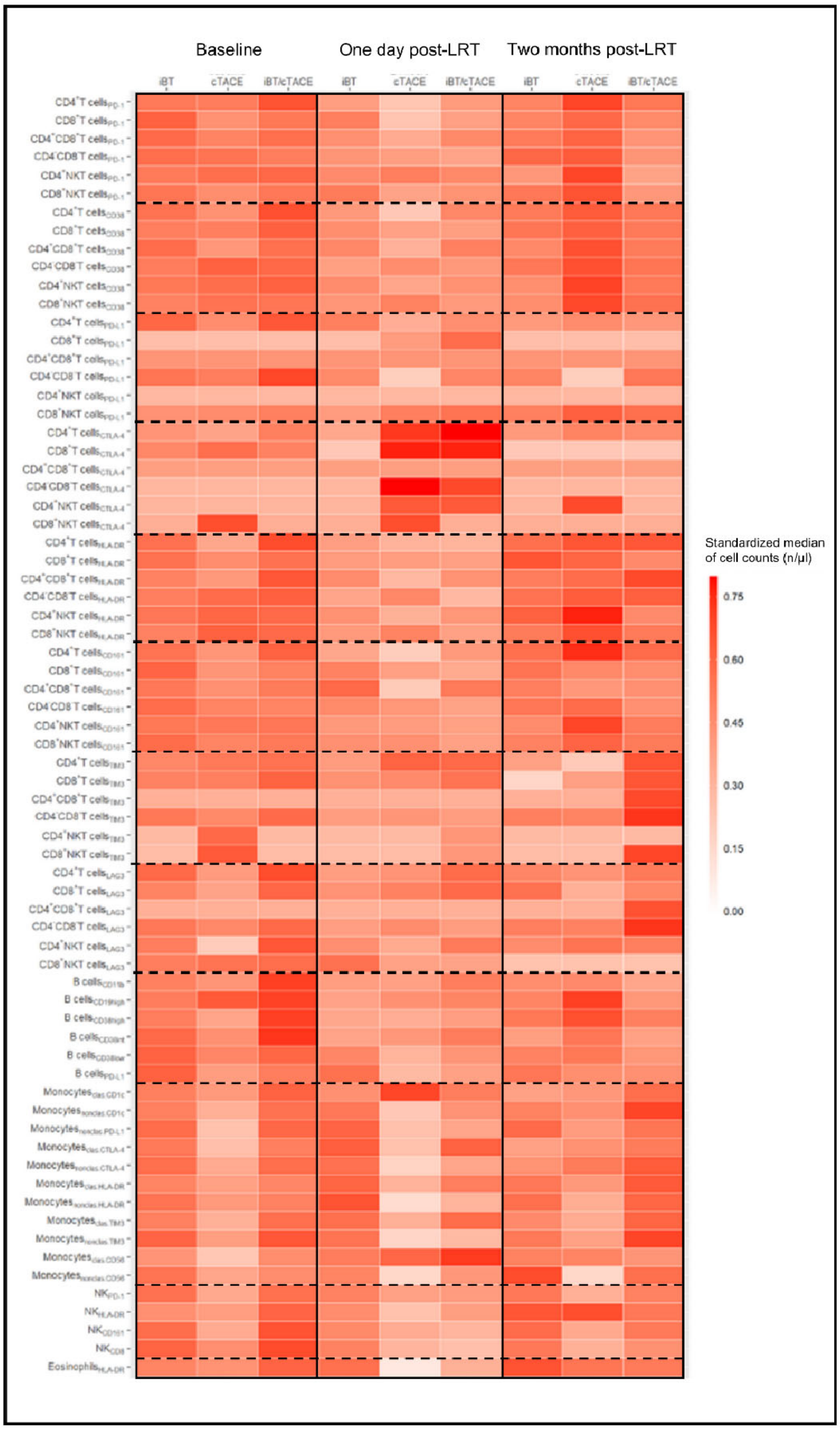
Baseline
One day post-LRT
Two months post-LRT
iBT
cTACE
iBT/cTACE
CD4$^{+}$T cells$_{PD-1}$
CD8$^{+}$T cells$_{PD-1}$
CD4$^{+}$CD8$^{+}$T cells$_{PD-1}$
CD4$^{-}$CD8$^{-}$T cells$_{PD-1}$
CD4$^{+}$NKT cells$_{PD-1}$
CD8$^{+}$NKT cells$_{PD-1}$
CD4$^{+}$T cells$_{CD38}$
CD8$^{+}$T cells$_{CD38}$
CD4$^{+}$CD8$^{+}$T cells$_{CD38}$
CD4$^{-}$CD8$^{-}$T cells$_{CD38}$
CD4$^{+}$NKT cells$_{CD38}$
CD8$^{+}$NKT cells$_{CD38}$
CD4$^{+}$T cells$_{PD-L1}$
CD8$^{+}$T cells$_{PD-L1}$
CD4$^{+}$CD8$^{+}$T cells$_{PD-L1}$
CD4$^{-}$CD8$^{-}$T cells$_{PD-L1}$
CD4$^{+}$NKT cells$_{PD-L1}$
CD8$^{+}$NKT cells$_{PD-L1}$
CD4$^{+}$T cells$_{CTLA-4}$
CD8$^{+}$T cells$_{CTLA-4}$
CD4$^{+}$CD8$^{+}$T cells$_{CTLA-4}$
CD4$^{-}$CD8$^{-}$T cells$_{CTLA-4}$
CD4$^{+}$NKT cells$_{CTLA-4}$
CD8$^{+}$NKT cells$_{CTLA-4}$
CD4$^{+}$T cells$_{HLA-DR}$
CD8$^{+}$T cells$_{HLA-DR}$
CD4$^{+}$CD8$^{+}$T cells$_{HLA-DR}$
CD4$^{-}$CD8$^{-}$T cells$_{HLA-DR}$
CD4$^{+}$NKT cells$_{HLA-DR}$
CD8$^{+}$NKT cells$_{HLA-DR}$
CD4$^{+}$T cells$_{CD161}$
CD8$^{+}$T cells$_{CD161}$
CD4$^{+}$CD8$^{+}$T cells$_{CD161}$
CD4$^{-}$CD8$^{-}$T cells$_{CD161}$
CD4$^{+}$NKT cells$_{CD161}$
CD8$^{+}$NKT cells$_{CD161}$
CD4$^{+}$T cells$_{TIM3}$
CD8$^{+}$T cells$_{TIM3}$
CD4$^{+}$CD8$^{+}$T cells$_{TIM3}$
CD4$^{-}$CD8$^{-}$T cells$_{TIM3}$
CD4$^{+}$NKT cells$_{TIM3}$
CD8$^{+}$NKT cells$_{TIM3}$
CD4$^{+}$T cells$_{LAG3}$
CD8$^{+}$T cells$_{LAG3}$
CD4$^{+}$CD8$^{+}$T cells$_{LAG3}$
CD4$^{-}$CD8$^{-}$T cells$_{LAG3}$
CD4$^{+}$NKT cells$_{LAG3}$
CD8$^{+}$NKT cells$_{LAG3}$
B cells
B cells$_{CD19high}$
B cells$_{CD38high}$
B cells$_{CD38int}$
B cells$_{CD38low}$
B cells$_{PD-L1}$
Monocytes$_{clas CD1c}$
Monocytes$_{nonclas CD1c}$
Monocytes$_{nonclas PD-L1}$
Monocytes$_{clas CTLA-4}$
Monocytes$_{nonclas CTLA-4}$
Monocytes$_{clas HLA-DR}$
Monocytes$_{nonclas HLA-DR}$
Monocytes$_{clas TIM3}$
Monocytes$_{nonclas TIM3}$
Monocytes$_{clas CD56}$
Monocytes$_{nonclas CD56}$
NK$_{PD-1}$
NK$_{HLA-DR}$
NK$_{CD161}$
NK$_{CD8}$
Eosinophils$_{HLA-DR}$
Standardized median of cell counts (n/μl)
0.75
0.60
0.45
0.30
0.15
0.00

Fig. S5

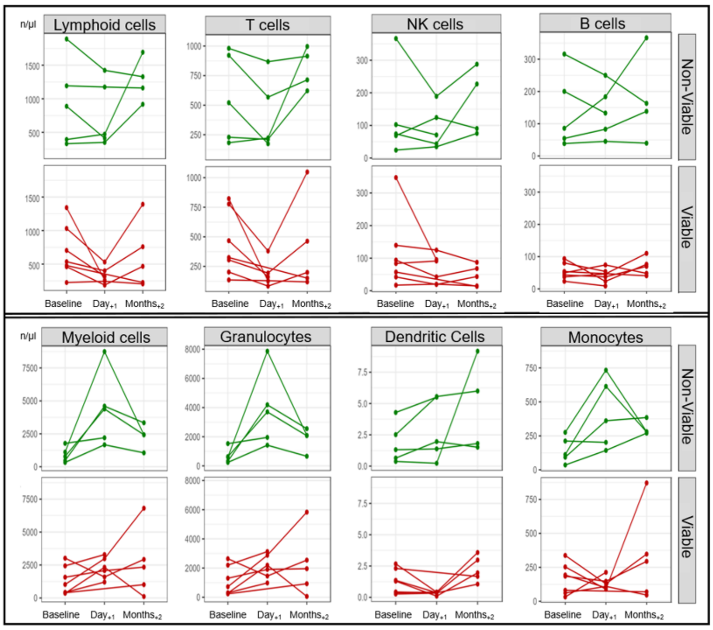

n/µl
Lymphoid cells
T cells
NK cells
B cells
Non-Viable
Viable
Myeloid cells
Granulocytes
Dendritic Cells
Monocytes
Baseline
Day+1
Months+2


Fig. S6

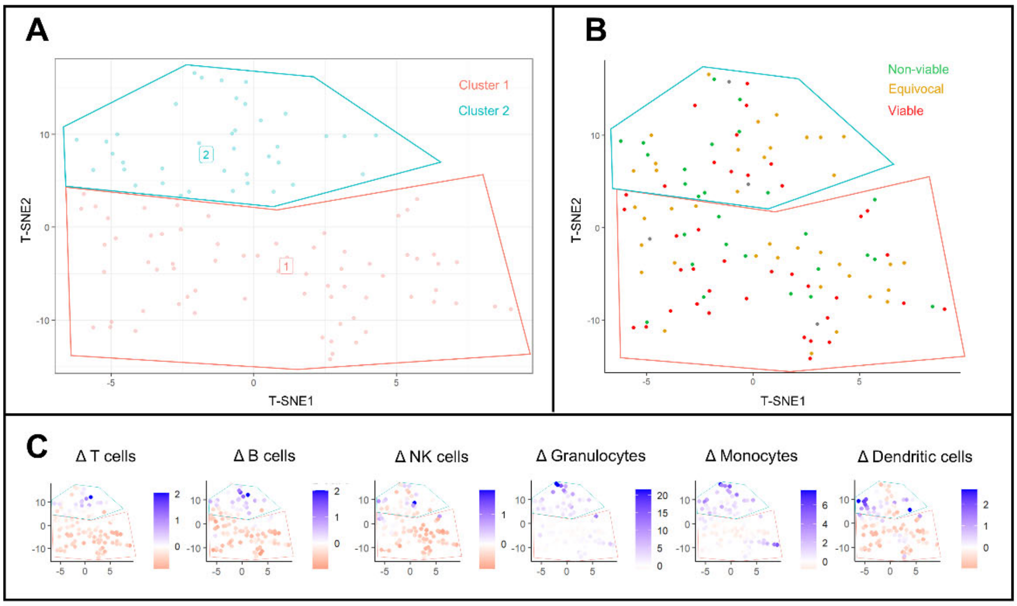

A
Cluster 1
Cluster 2
T-SNE2
T-SNE1
B
Non-viable
Equivocal
Viable
C
Δ T cells
Δ B cells
Δ NK cells
Δ Granulocytes
Δ Monocytes
Δ Dendritic cells


Fig. S7

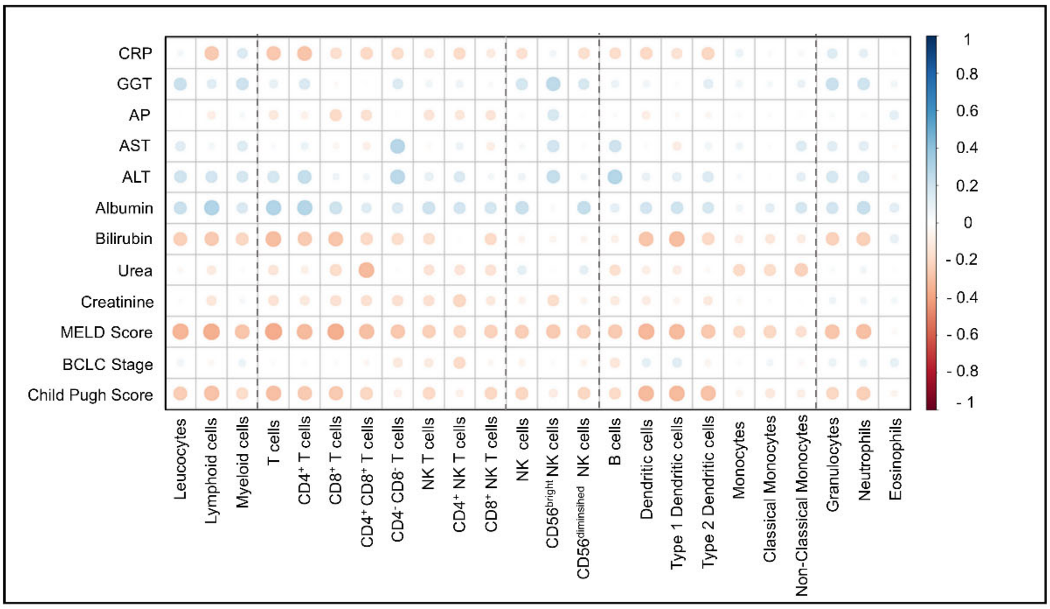
CRP
GGT
AP
AST
ALT
Albumin
Bilirubin
Urea
Creatinine
MELD Score
BCLC Stage
Child Pugh Score
Leucocytes
Lymphoid cells
Myeloid cells
T cells
CD4+ T cells
CD8+ T cells
CD4+ CD8+ T cells
CD4- CD8- T cells
NK T cells
CD4+ NK T cells
CD8+ NK T cells
NK cells
CD56bright NK cells
CD56diminished NK cells
B cells
Dendritic cells
Type 1 Dendritic cells
Type 2 Dendritic cells
Monocytes
Classical Monocytes
Non-Classical Monocytes
Granulocytes
Neutrophils
Eosinophils
1
0.8
0.6
0.4
0.2
0
- 0.2
- 0.4
- 0.6
- 0.8
- 1

**Supplementary figure legends**

**Fig. S1: Gating Strategy.** FSC-A and SSC-B-A scatters were used to identify leucocyte cell populations. SSC-A scatter was used to identify single cells vs cell debris, while SSC-H scatter was used to exclude red blood cell products. Next, CD45+ cell populations were gated according to major lymphoid and myeloid cell populations as described in the methods. Red asterisks indicate the use of an exclusion gate instead of an inclusion gate.

**Fig. S2 – Immune cell hierarchy.** Immune cell populations are mapped with their cell markers (CD - cluster of differentiation proteins) and sorted into hierarchical structure for gating of the flow cytometry results.

**Fig. S3 – Alterations of cytokines and chemokines following conventional transarterial chemoembolization (cTACE).** Dot plots reveal the absolute changes of pro- and inti-inflammatory cytokines and chemokines one day post-cTACE. The blue dotted line indicates the cohort's median Cohen's D of ± 0.29.

**Fig. S4 - Longitudinal changes of activation, exhaustion, and checkpoint molecules on immune cell populations stratified by type of locoregional therapies.** Heatmap showing standardized medians (SM, from 0 to 1) of checkpoints, activation, and exhaustion molecules on immune cell populations before and after interstitial brachytherapy (iBT), conventional transarterial chemoembolization (cTACE), or a combined cTACE/iBT. At one day post-LRT, SM of molecules on monocytes and B cells decreased, but SM of CTLA-4 on T cells increased

demonstrating the most profound effect in patients with cTACE. For these patients compared to the other treatment groups, higher expression of PD-1, HLA-DR, and CD161 on T cells and of molecules on B cells were observed at two months post-LRT.

**Fig. S5 - Associations of immune cell profiles with tumor response following conventional transarterial chemoembolization.** Line graphs show individual immune cell count alterations for completed treatment cycles of conventional transarterial chemoembolization (cTACE, n=27). Responders according to LI-RADS-TRA v2017 (green lines) generally showed higher cell counts of lymphoid and myeloid populations at all time points. Additionally, in responders, lymphoid cells demonstrated a steady increase in the longitudinal course, while in non-responders on follow-up imaging (red lines), lymphoid cells tended to decrease as early as one day but then re-increased at two months after LRT. Regarding myeloid cells, responders showed an increase one day post-LRT, whereas non-responders only showed a slight increase at two months post-LRT.

**Fig. S6 - Associations of immune cell profiles with tumor response.** Clustering analysis using immune alterations of major immune cell populations as well as CD4+ and CD8+ T cell subsets expressing PD1, PDL-1 and CTLA4 shows a similar clustering (TSNE-pattern) and immune cell subset distribution within two clusters as the results from the clustering analysis without including checkpoint molecules as independent clustering parameters. A) TSNE-clustering analysis based on immune cell alterations of major immune cell populations from baseline to one day post-LRT revealed two clusters of patients with different immunological responses. B) Patients

in the first cluster showed higher rates of remaining tumor viability according to LIRADS TRA v2017, while patients in the second cluster demonstrated more inflamed tumor response with higher rates of equivocal tumor. C) Patients from the first cluster show decreasing populations of T-, B-, NK cells, monocytes, and conventional dendritic cells, while patients from the second cluster showed no trends in lymphoid populations but an increase in conventional dendritic cells, monocytes, and granulocytes.

**Fig. S7 - Correlation of baseline immune cell counts with clinical and laboratory patient characteristics.** Correlation matrix shows the extent (size and color of the circles) and direction of the correlation of baseline immune cell counts with clinical and laboratory parameters (blue- parallel; red - antiparallel). Spearman correlation did not reveal any strong correlations, however trends in parallel correlations of immune cell counts with albumin, gamma-glutamyl transferase levels (γGT), Child-Pugh, and Model of End-Stage Liver Disease (MELD) score were found, respectively, while anti-parallel correlative trends were mainly observed between immune cell counts and C-reactive protein, bilirubin, urea, creatinine, and the Barcelona Clinic Liver Cancer (BCLC) score, respectively.

**Supplementary references**

1. Cossarizza A, Chang HD, Radbruch A, Abrignani S, Addo R, Akdis M, et al. Guidelines for the use of flow cytometry and cell sorting in immunological studies (third edition). Eur J Immunol. 2021; 51: 2708-3145.

2. Kapellos TS, Bonaguro L, Gemünd I, Reusch N, Saglam A, Hinkley ER, et al. Human Monocyte Subsets and Phenotypes in Major Chronic Inflammatory Diseases. Front Immunol. 2019; 10: 2035.

3. Poli A, Michel T, Thérésine M, Andrès E, Hentges F, Zimmer J. CD56bright natural killer (NK) cells: an important NK cell subset. Immunology. 2009; 126: 458-65.

4. Mousset CM, Hobo W, Woestenenk R, Preijers F, Dolstra H, van der Waart AB. Comprehensive Phenotyping of T Cells Using Flow Cytometry. Cytometry A. 2019; 95: 647-54.

5. Lencioni R, Petruzzi P, Crocetti L. Chemoembolization of hepatocellular carcinoma. Semin Intervent Radiol. 2013; 30: 3-11.

6. Ricke J, Wust P, Stohlmann A, Beck A, Cho CH, Pech M, et al. CT-guided interstitial brachytherapy of liver malignancies alone or in combination with thermal ablation: phase I-II results of a novel technique. Int J Radiat Oncol Biol Phys. 2004; 58: 1496-505.

7. Bretschneider T, Ricke J, Gebauer B, Streitparth F. Image-guided high-dose-rate brachytherapy of malignancies in various inner organs - technique, indications, and perspectives. J Contemp Brachytherapy. 2016; 8: 251-61.

8. Collettini F, Schnapauff D, Poellinger A, Denecke T, Schott E, Berg T, et al. Hepatocellular carcinoma: computed-tomography-guided high-dose-rate brachytherapy (CT-HDRBT) ablation of large (5-7 cm) and very large (>7 cm) tumours. Eur Radiol. 2012; 22: 1101-9.
