# Supplementary material for: Immunologic effects of locoregional therapies for unresectable hepatocellular carcinoma
Source: JHEP Rep. 2025 Aug 22;7(12):101555. doi: 10.1016/j.jhepr.2025.101555 (PMC12637058; doi:10.1016/j.jhepr.2025.101555)
Supplement: Multimedia component 4 [file mmc4.zip › Clinical Trial/DRKS - ImmuMITT registry protocol.pdf]

Immunologische Effekte minimal-invasiver Therapien in Patienten mit hepatozellulärem Karzinom

Organisatorische Daten

DRKS-ID der Studie:
DRKS00026994

Status der Rekrutierung:
Rekrutierung abgeschlossen, Studie läuft noch

Registrierungsdatum in DRKS:
07.12.2021

Letzte Aktualisierung in DRKS:
27.01.2023

Art der Registrierung:
Retrospektiv

Studienakronym/Studienabkürzung

ImmuMITT

Internetseite der Studie

Kein Eintrag

Allgemeinverständliche Kurzbeschreibung

ImmuMITT untersucht das periphere Immunzellprofil bei Patientinnen und Patienten mit hepatozellulärem Karzinom (HCC).

Die Entstehung von Leberkrebs ist eng an das Vorliegen einer chronisch-entzündlichen Lebererkrankung geknüpft, bei welcher Immunzellen und insbesondere die Fehlfunktion des Immunsystems entscheidend beteiligt sind.

In der Beobachtungsstudie werden Patienten mit Leberkrebs und der Empfehlung zur minimal-invasiven Therapie vor und nach der Therapie zu routinemäßigen Zeitpunkten Blut abgenommen und dieses auf das Vorliegen von Immunzellpopulationen sowie deren Aktivierungs- bzw. Erschöpfungsgrad untersucht. Das Ziel ist es, damit prognostische Aussagen über die Therapieoptionen zu tätigen, aber auch herauszufinden, welcher Patient insbesondere für eine Immunsystem-unterstützende Kombinationstherapie geeignet sein und profitieren könnte.

Wissenschaftliche Kurzbeschreibung

Das HCC tritt zumeist metachron und multizentrisch auf. In über 80% der Fälle liegt eine chronisch-entzündliche Lebererkrankung vor, welches ein insgesamt proinflammatorisches, tumorogenes Milieu erschafft. Beim nicht-resezierbaren hepatozellulären Karzinom (HCC) sind minimal-invasive, sogenannte lokoregionäre Therapien die leitliniengerechte Standardtherapien für die Behandlung des HCCs im frühen und intermediären Stadium. Aktuelle Innovationen in der Tumortherapie konzentrieren sich auf immunologische Interventionen, die das Ziel haben, die Barriere der Immunsuppression zu senken und die Ressourcen des körpereigenen Immunsystems zu stärken. Allerdings haben diese systemischen Ansätze beim HCC bisher kaum zu einem nennenswerten Überlebensvorteil geführt und während einige Systemtherapien wie bspw. die Behandlung mit Atelizumab/Bevazicumab und Sorafenib bereits die FDA-Zulassung für die Erstlinienbehandlung beim HCC im fortgeschrittenen Stadium erhalten haben, gibt es kaum Daten für die Behandlung des HCC in einem frühen und intermediären Stadium, insbesondere nicht für den kombinatorischen Einsatz mit lokoregionären Therapien. Deshalb zielt diese Studie darauf hinab, Patienten mit einem HCC und der Empfehlung zur lokoregionären Therapien zu beobachten, um so möglicherweise günstige immunologische Effekte aber auch neue prognostische Biomarker zu identifizieren.

Untersuchte Krankheit, Gesundheitsproblem

ICD10:
C22.0 - Leberzellkarzinom

Gesunde Probanden:
Nein

Interventionsgruppen, Beobachtungsgruppen

Arm 1:
Transarterielle Chemoembolisation

Arm 2:
Hochdosisbestrahlungstherapie (Brachytherapie) in Afterloading-Technik

Arm 3:
Transarterielle Chemoembolisation und konsekutiver Hochdosisbestrahlungstherapie (Brachytherapie) in Afterloading-Technik

Endpunkte

Primärer Endpunkt:
Therapieansprechen - gemessen anhand standardisierter, bildmorphologischer Kriterien u.a. (m)RECIST oder LIRADS-TRA v2017 zu jeweils definierten Zeitpunkten (nach 2 Monaten, nach 5 Monaten sowie anschließend alle 3-6 Monate postinterventionell)

Sekundärer Endpunkt:

Gesamtüberleben, Progressions-freies Überleben - gemessen anhand der Meldedaten aus dem Krankenhaus-eigenem Dokumentationssystem sowie nationalen Krebsregistern und ggf. Meldeämtern

Studiendesign

Studienzweck:
Prognose

Retrospektiv/Prospektiv:
Prospektive Datenerhebung

Studientyp:
Nicht-interventionell

Längsschnitt/Querschnitt:
Längsschnittstudie

Studientyp nicht-interventionell:
Epidemiologische Studie

Rekrutierung

Status der Rekrutierung:
Rekrutierung abgeschlossen, Studie läuft noch

Grund, falls Rekrutierung eingestellt oder zurückgezogen:
Kein Eintrag

Rekrutierungsorte

Rekrutierungsländer:
Deutschland

Anzahl Prüfzentren:
Monozentrisch

Rekrutierungsstandort(e):
Universitätsklinikum Charité - Universitätsmedizin Berlin Berlin

Rekrutierungszeitraum und Teilnehmerzahl

Geplanter Studienstart:
Kein Eintrag

Tatsächlicher Studienstart:
30.06.2020

Geplantes Studienende:
Kein Eintrag

Tatsächliches Studienende:
Kein Eintrag

Geplante Teilnehmeranzahl:
128

Tatsächliche Teilnehmeranzahl:
122

Einschlusskriterien

Geschlecht:
Alle

Mindestalter:
kein Mindestalter

Höchstalter:
kein Höchstalter

Weitere Einschlusskriterien:
nicht-resektables HCC (C22.0) mit der Tumorboard-Empfehlung zur minimal-invasiven Therapie

Ausschlusskriterien

andere lebereigenene und -fremde Tumore außer HCC

Adressen

Initiierung der Studie durch (Primärer Sponsor)

Charité Campus Virchow-Klinikum
Augustenburger Platz 1
13353 Berlin
Deutschland

Telefon:
Kein Eintrag

Fax:
Kein Eintrag

Kontakt per E-Mail:
Kein Eintrag

URL der Einrichtung:
http://www.charite.de *(Link: http://www.charite.de)*

Wissenschaftsinitiierte Studie (IST/IIT):
Ja

Kontakt für wissenschaftliche Anfragen

Klinik für Diagnostische und Interventionelle Radiologie - Charité Universitätsmedizin Berlin, CVK
Dr. med. Lynn Savic

Augustenburger Platz 1
13353 Berlin
Deutschland

Telefon:
+49 30 450 657 093

Fax:
Kein Eintrag

Kontakt per E-Mail:
lynn-jeanette.savic@charite.de

URL der Einrichtung:
https://radiologie.charite.de/forschung/forschungsschwerpunkte_und_arbeitsgruppen/minimal_invasive_tumortherapien_mitt_lab/ *(Link: https://radiologie.charite.de/forschung/forschungsschwerpunkte_und_arbeitsgruppen/minimal_invasive_tumortherapien_mitt_lab/)*

Kontakt für allgemeine Anfragen

Minimal-invasive Tumortherapie (MITT) Ambulanz
Augustenburger Platz 1
13353 Berlin
Deutschland

Telefon:
+49 30 450 527 235

Fax:
+49 30 450 553 928

Kontakt per E-Mail:
minimal-invasive-ambulanz@charite.de

URL der Einrichtung:
https://tumortherapie.charite.de/ *(Link: https://tumortherapie.charite.de/)*

Wissenschaftliche Leitung (PI)

Klinik für Diagnostische und Interventionelle Radiologie - Charité Universitätsmedizin Berlin, CVK
Dr. med. Lynn Savic
Augustenburger Platz 1
13353 Berlin
Deutschland

Telefon:
+49 30 450 657 093

Fax:

Kein Eintrag

Kontakt per E-Mail:
lynn-jeanette.savic@charite.de

URL der Einrichtung:
https://radiologie.charite.de/forschung/forschungsschwerpunkte_und_arbeitsgruppen/minimal_invasive_tumortherapien_mitt_lab/ *(Link: https://radiologie.charite.de/forschung/forschungsschwerpunkte_und_arbeitsgruppen/minimal_invasive_tumortherapien_mitt_lab/)*

Finanzierungsquellen

Kommerziell (Pharma, med.-techn. Industrie u. a.)

Guerbet GmbH
Otto-Volger-Str. 11
65843 Sulzbach
Deutschland

Telefon:
06196/762-0

Fax:
06196/73934

Kontakt per E-Mail:
med.wiss(at)guerbet-group.com

URL der Einrichtung:
https://www.guerbet.com/de-de/ *(Link: https://www.guerbet.com/de-de/)*

Ethikkommission

Adresse der Ethikkommission

Ethikkommission der Charité – Universitätsmedizin Berlin
Charitéplatz 1
10117 Berlin
Deutschland

Telefon:
(+49)30-450517222

Fax:
(+49)30-450517952

Kontakt per E-Mail:
ethikkommission@charite.de

URL der Einrichtung:
Kein Eintrag

Votum der federführenden Ethikkommission

Antragsdatum bei der Ethikkommission:
31.05.2019

Bearbeitungsnummer der Ethikkommission:
EA2/091/19

Votum der Ethikkommission:
Zustimmende Bewertung

Datum des Votums:
11.07.2019

Weitere Identifikationsnummern

Andere WHO-Primär-Register/Datenprovider-ID:
Kein Eintrag

EudraCT-Nr.:
Kein Eintrag

UTN (Universal Trial Number):
Kein Eintrag

EUDAMED-Nr.:
Kein Eintrag

IPD - Individual Participant Data / Teilnehmerbezogene Daten

Planen Sie, die teilnehmerbezogenen Daten (IPD) anderen Forschern anonymisiert zur Verfügung zu stellen?:
Nein

IPD Sharing Plan:
Kein Eintrag

Studienprotokoll und weitere Studiendokumente

Studienprotokolle:

Kein Eintrag

Abstract zur Studie:

Kein Eintrag

Weitere Studiendokumente:

Kein Eintrag

Hintergrundliteratur:

Kein Eintrag

Verwandte DRKS-Studien:

Kein Eintrag

Veröffentlichung der Studienergebnisse

Geplante Publikation:

2025

Publikationen/Studienergebnisse:

Kein Eintrag

Datum der ersten Publikation von Ergebnissen in einer Zeitschrift:

Kein Eintrag

DRKS-Eintrag erstmalig mit Ergebnissen publiziert:

Kein Eintrag

Grundlegende Ergebnisse

Basic Reporting/Ergebnistabellen:

Kein Eintrag

Kurzzusammenfassung der Ergebnisse:

Kein Eintrag

Alle Rechte vorbehalten. © BfArM, www.bfarm.de
