# Supplementary material for: Immunologic effects of locoregional therapies for unresectable hepatocellular carcinoma
Source: JHEP Rep. 2025 Aug 22;7(12):101555. doi: 10.1016/j.jhepr.2025.101555 (PMC12637058; doi:10.1016/j.jhepr.2025.101555)
Supplement: Multimedia component 4 [file mmc4.zip › Clinical Trial/ENG study information.pdf]

Patient information for the comparison group of the NAFLD cohort study of the Department of Hepatology and Gastroenterology, Charité - Universitätsmedizin Berlin, Campus Charité Mitte and Campus Virchow-Klinikum

Dear patient,

Dear lady, dear gentleman,

You have been diagnosed with chronic liver disease and have now been offered participation in a clinical trial. To ensure that you are as fully informed as possible before giving your consent, you will find a description of the project below.

Background

Non-alcoholic fatty liver disease (NAFLD) is the most common liver disease worldwide with a steadily increasing incidence and number of new cases. The incidence in Germany is currently estimated at around 20%, while in the USA, for example, it is 35%. NAFLD is often associated with overweight and obesity, type 2 diabetes mellitus, high blood pressure (arterial hypertension) and a lipid metabolism disorder (dyslipidemia). If the disease progresses, there is an increased risk of cardiovascular disease as well as liver-related complications such as liver cirrhosis or liver cancer (hepatocellular carcinoma, or HCC for short). The latter risk exists for patients in whom a fatty liver progresses to fatty liver inflammation (so-called fatty liver hepatitis or steatohepatitis). Despite intensive research, the pathogenesis of the disease has not yet been clarified with certainty. It is currently assumed that various damaging factors occur at the same time. According to the latest research, these include an unfavorable diet with a high proportion of fructose (mainly contained in industrially produced food and so-called soft drinks) and omega-6 fatty acids, as well as genetic factors (e.g. variants in the so-called PNPLA3 gene) and a disturbed intestinal microflora (so-called dysbiosis). All of these factors promote fatty degeneration of the liver, which in turn causes an inflammatory reaction in the liver and can lead to fatty liver hepatitis. If this lasts long enough, “wound healing processes” are activated, which lead to an accumulation of connective tissue in the liver (fibrosis). If these processes are not interrupted, complete connective tissue remodeling can occur in the liver, which is referred to as liver cirrhosis and can lead to a loss of liver function. Liver cirrhosis itself is in turn the most important risk factor for hepatocellular carcinoma (HCC).

What is the aim of the study?

The purpose of the study is to investigate a liver disease. We would like to characterize patients with NAFLD in order to better understand the development, prognosis and possible treatment options. We also want to develop new methods for investigating fatty liver. In future, this may help to make a diagnosis independently of the direct examination of liver tissue by means of a liver biopsy, for example, and to better assess the progression of the disease. Furthermore, the connection between NAFLD and other metabolic diseases - including lipometabolic disorders and diabetes - is to be investigated. For this purpose, however, it is scientifically necessary to compare patients with NAFLD with patients with other chronic liver diseases. We are therefore also asking these patients for their consent and participation.

A large group (cohort) of patients with NAFLD and, as a comparison group, a large cohort of patients with other chronic liver diseases are to be established at our clinic. With your liver disease, you would be part of the comparison/control group. This overall cohort (NAFLD and

comparison groups) will then be used in studies to analyze the development, diagnosis and further course of NAFLD.

Inclusion criteria and study procedure

The Medical Clinic for Hepatology and Gastroenterology, including the Metabolic Diseases Unit, Charité - Universitätsmedizin Berlin, Campus Charité Mitte and Campus Virchow-Klinikum, will be responsible for study inclusion. Approximately 3000 study participants will be included. All patients over the age of 18 who have given their written consent to participate in the study and who suffer from non-alcoholic fatty liver disease (NAFLD) can take part in this study.

However, it is also possible for you to participate if you suffer from another chronic liver disease. In this case, you will be asked to consent and participate in our local NAFLD cohort study as part of the comparison group.

If you agree to participate in the study, the study doctor will collect information about you and the severity of your disease. This data includes your age, previous blood tests, radiological and sonographic examinations, physical activity and diet. Previous medical information that is relevant for study purposes will also be recorded. You will be asked about your state of health and lifestyle habits using questionnaires. In addition, you will be asked to provide urine and stool samples, which are not part of routine medical practice. The above-mentioned study-related measures require an additional 20 minutes per annual treatment appointment.

It is possible that a liver biopsy will be taken as part of routine medical care and if medically necessary, or that a liver biopsy has already been taken in the past. A small portion of the liver biopsy, which is not required for clinical diagnostics, is to be used for the purpose of research into liver diseases. However, you can also participate without a liver biopsy. No liver biopsy will be performed as part of this study. If you take part in the study, up to 6 teaspoons (approx. 60 ml) of blood will also be taken as part of planned blood samples. It cannot be assumed that taking this amount of blood will have any effect on health.

Imaging procedures - including MRI and ultrasound examinations - are also evaluated. If such an examination has been carried out or has been scheduled as part of your medical care, we would ask you to provide us with your findings for future research projects.

You will also be asked to give your consent to be contacted so that the study team can contact you should studies arise in the future in which you could participate. However, this does not mean that you have to take part in further studies. Any further investigations or studies will be reviewed and assessed in advance by the relevant ethics committees.

During the course of the study, third parties involved in the study for quality assurance reasons may also have access to the data (so-called monitors), but they are also subject to a duty of confidentiality.

What happens to the data collected during the study?

Your study data will be recorded electronically on the Charité's own server at the study center at the Medical Clinic for Hepatology and Gastroenterology, including the Metabolic Diseases Unit, Charité - Universitätsmedizin Berlin, Campus Charité Mitte and Campus Virchow-Klinikum.

Your data is pseudonymized. Pseudonymization means that your data will be assigned to a code without your date of birth or name. Your medical data will be entered into the study register by the study doctor and his scientific staff at the study center. The electronically stored data is

stored on encrypted and password-protected servers, so that both access control and data security are in place. The records are kept for 15 years.

Decryption only takes place under the conditions prescribed by law. In the context of this planned study, only the study personnel, who are bound by medical confidentiality, will have access to your personal data. The key (code) remains with the study doctor.

What happens to my samples?

As part of the research study at our clinic (NAFLD cohort of the Charité), excess material (e.g. liver tissue) or easily obtainable bodily excretions (blood, stool and urine) will be collected at the time of your consent and in the further course of the study - when you present again. As part of the planned examinations, your samples will be sent to other laboratories involved in the research project for analysis. This will only take place after pseudonymization.

Genetic tests will also be carried out as part of this research study. Genetic changes in the blood taken will be examined to find out which of your genes contribute to which disease characteristics of your liver disease and to what extent. This involves an examination of your entire genetic material (genome). Genes contribute to a person's uniqueness and are only examined with your express consent. The connection between genetic changes and NAFLD or the diseases associated with it is unclear and, according to current knowledge, the genetic examination will not have any consequences for your treatment. The genetic material is used exclusively to test for genes that are thought to be important in NAFLD, other chronic liver diseases and metabolic diseases such as type 2 diabetes. Various methods are used for this purpose. These can, for example, examine individual, previously selected genes or search for larger changes such as gains or losses of genetic material. Newer methods make it possible to search for changes in the entire human genome (i.e. the entirety of all genes and the regions in between). The evaluation of such studies is always carried out exclusively with regard to genes that appear to be relevant for NAFLD and chronic liver diseases.

You or your family doctor will not be informed of any additional findings from the genetic test. The examinations serve to gain knowledge of disease correlations in the group of all patients. Publication of the entirety of your genetic information (whole genome) is excluded without your express written consent.

Your blood and tissue samples will be stored at the Medical Clinic for Hepatology and Gastroenterology, including the Metabolic Diseases Unit, Charité - Universitätsmedizin Berlin, Campus Charité Mitte and Campus Virchow-Klinikum. It is possible that samples are stored for many years before they are used for research purposes. DNA (genetic information), RNA (RNA is the “blueprint” for proteins in the cells), proteins, fats or other blood components that indicate inflammation and liver damage are isolated and examined from the samples. Your samples will be stored in the biobank for the duration of the study and beyond, to enable future analysis. You will be asked for permission for the collected samples to be used for future research projects.

Do you have to participate in this study and can you change your decision?

Your participation in this study is voluntary. If you decide not to participate, you will not suffer any disadvantages. Your participation in the study has no influence on your medical treatment, about which you have already been informed by your attending physician. Additional visits to the clinic are not necessary. You are free to withdraw from the study (end your participation) at

any time and without giving reasons. If you wish to withdraw from the research study, please inform your study doctor. You can do this at any time and without giving reasons.

What are the advantages and disadvantages of participating in a study?

There is no individual benefit to be expected beyond the scientific findings on risk factors for non-alcoholic fatty liver disease. In principle, you will not incur any relevant risks by participating. The blood sample is taken during routine blood sampling, so that any risk does not arise specifically from the study.

Is there any additional insurance cover?

As you are participating as a patient of our clinic and as the examinations are carried out as part of your routine check-ups, there is no separate insurance cover for the study. The Charité employees involved in the study (study physicians, study nurses, etc.) are insured by the Charité's public liability insurance against liability claims that could result from their culpable conduct. There is also no insurance for incidents that happen to you on the way to the examination or on your way back after the examination.

Is there an expense allowance?

You will not be reimbursed for your participation in the examination. However, you will not incur any costs as a result of your participation in the study.

Will I be personally informed of the results of the studies?

No. Neither you nor the doctors treating you will be informed of the results. The results will also not be recorded in your medical records. The scientific observations will be published at medical congresses and in scientific publications. Your name or other identifying features will not be included. If you wish, we will be happy to inform you about the results of this study.

Who organizes and finances the studies?

This NAFLD cohort study was designed by the Medical Department of Hepatology and Gastroenterology, including the Metabolic Diseases Unit, Charité-Universitätsmedizin Berlin, Campus Charité Mitte and Campus Virchow-Klinikum, and is not funded by third parties (a so-called IIT study, Investigator Initiated Trial: a study initiated by scientists, universities or study centers without commercial interest).

Disclosure of information outside the study center

Within the framework of research consortia, other scientists may be given access to some of your clinical data and samples - insofar as this is necessary for the development of the new tests. All information is pseudonymized by a code. This code remains at the study center and can only be traced by the doctors at the study center and the study staff, who are also subject to confidentiality.

The samples and data are used for the following purposes

o Research into new non-invasive markers to identify the stage of your liver disease

o Publication of data and use for educational purposes in anonymized form

o Development of future scientific questions regarding liver disease research

Information on data protection

The processing of your personal data is only lawful with your consent (Article 6 General Data Protection Regulation; GDPR). The study results are protected against unauthorized access. Decryption only takes place under the conditions prescribed by law.

The legal basis for the processing of your personal data in clinical studies is your voluntary written consent in accordance with the General Data Protection Regulation (GDPR) and the Declaration of Helsinki (Declaration of the World Medical Association on Ethical Principles for Medical Research Involving Human Subjects) and the Guideline for Good Clinical Practice (GCP).

You have the following rights with regard to your data:

Right of access: you have the right of access to the personal data concerning you that is collected, processed or, if applicable, transmitted to third parties in the context of the clinical trial (provision of a copy) (Article 15 GDPR).

Right to rectification: You have the right to have inaccurate personal data concerning you rectified (Articles 16 and 19 GDPR).

Right to erasure: You also have the right to the erasure of all personal data concerning you, insofar as this is possible (e.g. if this data is no longer necessary for the purpose for which it was collected (Articles 17 and 19 GDPR)).

Right to restriction of processing: Under certain conditions, you have the right to request a restriction of processing, i.e. the data may only be stored, not processed. You must request this. To do so, please contact your examiner or the data protection officer of the test center (Articles 18 and 19 GDPR).

Right to data portability: You have the right to receive the personal data concerning you that you have provided to the person responsible for the clinical study/clinical trial. This means that you can request that this data be transferred either to you or, if technically possible, to another body designated by you (Article 20 GDPR).

Right to object: You have the right to object at any time to specific decisions or measures concerning the processing of personal data relating to you (Art. 21 GDPR, Section 36 BDSG-new). Such processing will then generally no longer take place.

Consent to the processing of personal data and the right to withdraw this consent: The processing of your personal data is only lawful with your consent (Article 6 GDPR). You have the right to withdraw your consent to the processing of personal data at any time and can also decide whether the data already collected should be deleted or may continue to be used (Article 7 (3) GDPR).

If you would like to exercise any of these rights, please contact

Responsible study physicians:

PD Dr. med. Münevver Demir (study director) and Prof. Dr. Frank Tacke (representative)

Charité - Universitätsmedizin Berlin, Medical Clinic specializing in Hepatology and Gastroenterology including the Metabolic Diseases Unit, Campus Virchow-Klinikum, Augustenburger Platz 1, 13353 Berlin

Tel. + 49 30 450 66 53 37; Fax: +49 30 450 55 39 42

The data protection officer of the controller is

Charité - Universitätsmedizin Berlin

Campus Charité Mitte

Data Protection Office

Charitéplatz 1

10117 Berlin
