# Supplementary material for: Immunologic effects of locoregional therapies for unresectable hepatocellular carcinoma
Source: JHEP Rep. 2025 Aug 22;7(12):101555. doi: 10.1016/j.jhepr.2025.101555 (PMC12637058; doi:10.1016/j.jhepr.2025.101555)
Supplement: Multimedia component 4 [file mmc4.zip › Clinical Trial/GER study information.pdf]

Leitung: Prof. Dr. F. Tacke

Medizinische Klinik m. S.
Hepatologie und Gastroenterologie
einschl. Arbeitsbereich Stoffwechselerkrankungen
Zertifiziertes Darmkrebszentrum
ENETS Center of Excellence (ENETS CoE)
Augustenburger Platz 1 | 13353 Berlin

Telefon: +49 30 450 553 022
Telefax: +49 30 450 553 902
E-Mail: gastro-cvk@charite.de
https://hepatologie-gastroenterologie.charite.de

06.01.2020

**Patienteninformation für die Vergleichsgruppe zur NAFLD-Kohortenstudie der Medizinischen Klinik m. S. Hepatologie und Gastroenterologie einschl. Arbeitsbereich Stoffwechselerkrankungen, Charité - Universitätsmedizin Berlin, Campus Charité Mitte und Campus Virchow-Klinikum**

***Sehr geehrte Patientin, sehr geehrter Patient,***
***Sehr geehrte Dame, sehr geehrter Herr,***

bei Ihnen wurde eine chronische Lebererkrankung festgestellt und Ihnen wurde nun die Teilnahme an einer klinischen Studie vorgeschlagen. Damit Sie vor Ihrer Einwilligung möglichst umfassend informiert sind, finden Sie nachstehend eine Beschreibung des Vorhabens.

***Hintergrund***

Die Nichtalkoholische Fettlebererkrankung (kurz: NAFLD) ist weltweit die häufigste Lebererkrankung mit stetig steigender Krankheitshäufigkeit und Anzahl an Neuerkrankungen. Die Häufigkeit wird in Deutschland aktuell auf ca. 20% geschätzt, während sie in den USA z.B. bei 35% liegt. Die NAFLD ist häufig mit Übergewicht und Fettsucht (Adipositas), einem Diabetes mellitus Typ 2, einem Bluthochdruck (arterielle Hypertonie) und einer Fettstoffwechselstörung (Dyslipidämie) vergesellschaftet. Die Bedeutung der Erkrankung liegt bei einem Fortschreiten darin, ein erhöhtes Risiko für Herz-Kreislauferkrankungen zu haben, aber auch leberbezogene Komplikationen wie eine Leberzirrhose oder einen Leberkrebs (Hepatocelluläres Carcinom, kurz: HCC) zu entwickeln. Letzteres Risiko besteht für Patienten, bei denen eine Fettleber zu einer Fettleberentzündung (sog. Fettleberhepatitis oder Steatohepatitis) voranschreitet. Die Krankheitsentstehung ist trotz intensiver Forschung noch nicht sicher geklärt. Aktuell geht man von einem gleichzeitigen Auftreten von unterschiedlichen schädigenden Faktoren aus. Hierzu zählen nach dem aktuellsten Stand der Forschung, unter anderem eine ungünstige Ernährungsweise mit einem hohen Anteil an Fructose (v.a. enthalten in industriell gefertigter Nahrung und sog. Softdrinks) und Omega-6-Fettsäuren, sowie genetische Faktoren (z.B. Varianten im sog. PNPLA3-Gen) und eine gestörte Darm-Mikroflora (sog. Dysbiose). Alle diese Faktoren begünstigen eine Leberverfettung, die wiederum in der Leber eine Entzündungsreaktion verursacht und zu einer Fettleberhepatitis führen kann. Dauert diese lange genug an, werden „Wundheilungsprozesse“ aktiviert, die zu einer Einlagerung von Bindegewebe in der Leber führen (Fibrose). Werden diese Prozesse nicht unterbrochen, kann es zu einem kompletten bindegewebigen Umbau in der Leber kommen, welches als Leberzirrhose bezeichnet wird und

zu einem Funktionsverlust der Leber führt. Die Leberzirrhose selbst ist wiederum der wichtigste Risikofaktor für ein Hepatocelluläres Carcinom (HCC).

***Was ist das Ziel der Studie?***
Die Studie dient der Erforschung einer Lebererkrankung. Wir möchten gerne Patienten mit einer NAFLD charakterisieren, um die Entstehung, Prognose sowie auch mögliche Therapieoptionen besser zu verstehen. Ebenso sollen neue Methoden zur Untersuchung der Fettleber entwickelt werden. Dies kann helfen, zukünftig z.B. die Diagnose auch unabhängig von der direkten Untersuchung des Lebergewebes mittels einer Leberbiopsie zu stellen und den Verlauf der Erkrankung besser einzuschätzen. Weiterhin soll der Zusammenhang von NAFLD und anderen Erkrankungen des metabolischen Formenkreises – dazu gehören Fettstoffwechselstörungen und Diabetes – untersucht werden. Hierfür ist es aber wissenschaftlich erforderlich, die Patienten mit NAFLD mit Patienten mit anderen chronischen Lebererkrankungen zu vergleichen. Daher bitten wir auch diese Patienten um Einwilligung und Teilnahme.

An unserer Klinik soll eine große Gruppe (Kohorte) von Patienten mit NAFLD und als Vergleichsgruppe eine große Kohorte von Patienten mit anderen chronischen Lebererkrankungen aufgebaut werden. Mit Ihrer Lebererkrankung wären Sie Teil der Vergleichs-/Kontrollgruppe. Aus dieser Gesamt-Kohorte (NAFLD und Vergleichsgruppen) sollen dann im Rahmen von Studien Analysen zur Entstehung, der Diagnose und dem weiteren Verlauf der NAFLD durchgeführt werden.

***Einschlusskriterien und Ablauf der Studie***
Der Studieneinschluss erfolgt durch die Medizinische Klinik m.S. Hepatologie und Gastroenterologie einschl. Arbeitsbereich Stoffwechselerkrankungen, Charité - Universitätsmedizin Berlin, Campus Charité Mitte und Campus Virchow-Klinikum. Es werden ungefähr 3000 Studienteilnehmer/-innen eingeschlossen. An dieser Studie können alle Patienten teilnehmen, die über 18 Jahre alt sind, ihr schriftliches Einverständnis zur Teilnahme an der Studie gegeben haben und an einer Nichtalkoholischen Fettlebererkrankung (NAFLD) leiden.

Eine Teilnahme ist aber auch für Sie möglich, da Sie an einer anderen chronischen Lebererkrankung leiden. In diesem Fall werden Sie als Teil der Vergleichsgruppe um Einwilligung und Teilnahme an unserer lokalen NAFLD-Kohortenstudie gebeten.

Sollten Sie der Studienteilnahme zustimmen, wird der Studienarzt/die Studienärztin Informationen zu Ihnen und der Erkrankungsausprägung erheben. Zu diesen Daten zählen Ihr Alter, bereits durchgeführte Blutuntersuchungen, radiologische und sonographische Untersuchungen, die körperliche Aktivität und die Art der Ernährung. Auch frühere medizinische Informationen, die für Studienzwecke relevant sind, sollen erfasst werden. Sie werden mit Fragebögen zu Ihrem Gesundheitszustand und Ihren Lebensgewohnheiten befragt. Zusätzlich werden Sie gebeten, Urin- und Stuhlproben abzugeben, die nicht Teil der medizinischen Routine sind. Die genannten studienbedingten Maßnahmen erfordern einen zusätzlichen Zeitaufwand von 20 Minuten pro jährlichen Behandlungstermin.

Möglicherweise ist im Rahmen der routinemäßigen medizinischen Versorgung und bei Vorliegen einer entsprechenden medizinischen Notwendigkeit die Entnahme einer Leberbiopsie vorgesehen oder es wurde bereits in der Vergangenheit eine Leberbiopsie entnommen. Von der Leberbiopsie soll ein kleiner Teil, der nicht für die klinische Diagnostik benötigt wird, für den Zweck der Forschung über Lebererkrankungen eingesetzt werden. Allerdings können Sie auch ohne das Vorliegen einer Leberbiopsie teilnehmen. Im Rahmen der Durchführung dieser Studie erfolgt keine Leberbiopsie. Bei Studienteilnahme werden zusätzlich im Rahmen von geplanten Blutproben eine Menge von bis zu 6 Teelöffel (ca. 60 ml) Blut abgenommen. Ein Einfluss auf die Gesundheit bei der Entnahme dieser Menge an Blut ist nicht anzunehmen.

Auch bildgebende Verfahren – dazu gehören MRT und Ultraschalluntersuchungen – werden ausgewertet. Sollte eine derartige Untersuchung durchgeführt worden sein oder ist diese im

Rahmen der medizinischen Versorgung geplant, würden wir Sie um Überlassung der Befunde auch für zukünftige Forschungsvorhaben bitten.

Sie werden auch gebeten, Ihre Zustimmung zur Kontaktaufnahme zu geben, damit das Studienteam Sie kontaktieren kann, sollten sich in der Zukunft Studien ergeben, an denen Sie teilnehmen könnten. Das bedeutet aber nicht, dass Sie an weiteren Studien teilnehmen müssen. Jegliche weiteren Untersuchungen oder Studien werden vorab von den zuständigen Ethikkommissionen geprüft und beurteilt.

Im Rahmen der Durchführung der Studie, können auch Dritte, die an der Studie aus Gründen der Qualitätssicherung beteiligt sind, Einsicht nehmen (sog. Monitore), sie unterliegen aber auch der Schweigepflicht.

***Was passiert mit den Daten, die während der Studie gesammelt wurden?***
Ihre Studiendaten werden im Studienzentrum an der Medizinischen Klinik m. S. Hepatologie und Gastroenterologie einschl. Arbeitsbereich Stoffwechselerkrankungen, Charité - Universitätsmedizin Berlin, Campus Charité Mitte und Campus Virchow-Klinikum elektronisch auf Charité-eigenem Server aufgezeichnet.

Ihre Daten sind pseudonymisiert. Pseudonymisierung bedeutet, dass Ihre Daten einem Code ohne Angaben von Geburtsdatum oder Namen, zugeordnet werden. Ihre medizinischen Daten werden von dem Studienarzt und seinen wissenschaftlichen Mitarbeitern am Studienzentrum in das Studienregister eingegeben. Die elektronisch gespeicherten Daten sind auf verschlüsselten und Passwort-geschützten Servern gespeichert, so dass sowohl eine Zugangskontrolle als auch Datensicherheit besteht. Die Aufzeichnungen werden für 15 Jahre aufgehoben.

Eine Entschlüsselung erfolgt nur unter den vom Gesetz vorgeschriebenen Voraussetzungen. Im Rahmen dieser geplanten Studie hat nur das unter ärztlicher Schweigepflicht stehende Studienpersonal Zugang zu Ihren personenbezogenen Daten. Der Schlüssel (Code) bleibt beim Studienarzt.

***Was passiert mit meinen Proben?***
Im Rahmen der Forschungsstudie unserer Klinik (NAFLD-Kohorte der Charité) werden überschüssiges Material (z.B. Lebergewebe) oder einfach zu gewinnende Körperausscheidungen (Blut, Stuhl und Urin) zum Zeitpunkt Ihrer Zustimmung und im weiteren Verlauf – bei erneuter Vorstellung – gesammelt. Ihre Proben werden im Rahmen der geplanten Untersuchungen an weitere am Forschungsprojekt beteiligte Untersuchungslabore gesendet, um dort analysiert zu werden. Dies erfolgt erst nach Pseudonymisierung.

Im Rahmen dieser Forschungsstudie sollen auch genetische Untersuchungen durchgeführt werden. Dabei werden in dem entnommenen Blut genetische Veränderungen untersucht, um herauszufinden, welche Ihrer Gene in welchem Umfang zu welchen Krankheitsmerkmalen ihrer Lebererkrankung beitragen. Hierzu ist eine Untersuchung Ihrer gesamten Erbsubstanz (Genom) vorgesehen. Die Gene tragen zu der Eigenartigkeit eines Menschen bei und werden nur mit Ihrer ausdrücklichen Zustimmung untersucht. Der Zusammenhang von genetischen Veränderungen und der NAFLD, bzw. den damit verbundenen Erkrankungen ist unklar und eine Konsequenz der genetischen Untersuchung für Ihre Behandlung ergibt sich nach heutigem Wissensstand nicht. Dabei wird das genetische Material ausschließlich zur Untersuchung auf Gene verwandt, für die eine Bedeutung bei der NAFLD, anderen chronischen Lebererkrankungen und Stoffwechselerkrankungen wie z.B. dem Typ 2 Diabetes angenommen wird. Dabei werden verschiedene Methoden eingesetzt. Diese können z.B. einzelne, zuvor ausgewählte Gene untersuchen, oder nach größeren Veränderungen wie Hinzugewinnen oder Verlusten genetischen Materials suchen. Neuere Methoden erlauben es, im gesamten Genom des Menschen (d.h. der Gesamtheit aller Gene und der dazwischenliegenden Regionen) nach Veränderungen zu suchen. Die Auswertung derartiger Studien geschieht immer ausschließlich im Hinblick auf Gene, die für die NAFLD und chronische Lebererkrankungen relevant erscheinen.

Mögliche Zusatzbefunde der genetischen Untersuchung werden Ihnen oder Ihrem Hausarzt nicht mitgeteilt. Die Untersuchungen dienen dem Erkenntnisgewinn von Erkrankungszusammenhängen in der Gruppe aller Patienten. Eine Veröffentlichung der Gesamtheit Ihrer Erbinformation (Gesamtgenom) ist ohne Ihre ausdrückliche schriftliche Einwilligung hierzu ausgeschlossen.

Ihre Blut- und Gewebeproben werden an der Medizinischen Klinik m. S. Hepatologie und Gastroenterologie einschl. Arbeitsbereich Stoffwechselerkrankungen, Charité - Universitätsmedizin Berlin, Campus Charité Mitte und Campus Virchow-Klinikum gelagert. Es ist möglich, dass Proben über viele Jahre gelagert werden, bevor sie für Forschungszwecke eingesetzt werden. Aus den Proben wird DNS (genetische Erbinformation), RNS (RNS sind der "Bauplan" für Proteine in den Zellen), Proteine, Fette oder andere Blutbestandteile, die Entzündung und Leberschädigung anzeigen, isoliert und untersucht. Die Aufbewahrung Ihrer Proben in der Biobank erfolgt für den Zeitraum der Studie und darüber hinaus, um auch in der Zukunft Analysen zu ermöglichen. Sie werden um Erlaubnis gebeten, dass die gesammelten Proben auch für zukünftige Forschungsvorhaben verwendet werden können.

***Müssen Sie an dieser Studie teilnehmen und können Sie ihre Entscheidung ändern?***
Ihre Teilnahme an dieser Studie ist freiwillig. Wenn Sie sich gegen die Teilnahme entscheiden, entstehen damit für Sie keinerlei Nachteile. Ihre Teilnahme an der Studie hat keinen Einfluss auf Ihre medizinische Behandlung, über die Sie bereits von Ihrem behandelnden Arzt aufgeklärt worden sind. Zusätzliche Besuche in der Klinik sind nicht erforderlich. Sie haben die freie Entscheidung, jederzeit und ohne Angaben von Gründen aus der Studie auszuscheiden (Ihre Teilnahme zu beenden). Wenn Sie aus der Forschungsstudie ausscheiden möchten, informieren Sie bitte Ihren Studienarzt. Dies können Sie jederzeit und ohne Angabe von Gründen tun.

***Welche Vor- und Nachteile entstehen für mich bei einer Studienteilnahme?***
Ein individueller Vorteil, der über die wissenschaftlichen Erkenntnisse zu Risikofaktoren für eine Nichtalkoholische Fettlebererkrankung hinausgeht, ist nicht zu erwarten. Durch die Teilnahme entstehen Ihnen im Grundsatz keine relevanten Risiken. Die Blutentnahme erfolgt während der ohnehin durchgeführten Routine-Blutentnahme, so dass das etwaige Risiko nicht spezifisch durch die Studie entsteht.

***Gibt es einen zusätzlichen Versicherungsschutz?***
Da Sie als Patient unserer Klinik teilnehmen und da die Untersuchungen im Rahmen Ihrer routinemäßigen Kontrollen erfolgen, besteht keine separate Probandenversicherung für die vorliegende Studie. Die an der Studie beteiligten Mitarbeiter der Charité (Studienärzte und -ärztinnen, Studienschwestern und -pfleger etc.) sind durch die Betriebshaftpflichtversicherung der Charité gegen Haftpflichtansprüche, welche aus ihrem schuldhaften Verhalten resultieren könnten, versichert. Auch besteht keine Versicherung für Zwischenfälle, die Ihnen auf dem Weg zur Untersuchung oder nach dem Ende der Untersuchung auf Ihrem Rückweg widerfahren.

***Gibt es eine Aufwandsentschädigung?***
Eine Aufwandsentschädigung wird Ihnen für Ihre Teilnahme an der Untersuchung nicht gezahlt. Es entstehen Ihnen jedoch auch keinerlei Kosten durch die Teilnahme an der Studie.

***Werden mir die Ergebnisse der Studien persönlich mitgeteilt?***
Nein. Weder Ihnen noch den behandelnden Ärzten werden diese Ergebnisse mitgeteilt. Die Ergebnisse werden auch nicht in Ihren medizinischen Unterlagen der Krankenversorgung aufgezeichnet. Die erhobenen wissenschaftlichen Beobachtungen werden auf medizinischen Kongressen und im Rahmen von wissenschaftlichen Veröffentlichungen publiziert. Ihr Name oder weitere Identitätsmerkmale sind dabei nicht enthalten. Wenn Sie dies wünschen, informieren wir sie gerne über die Ergebnisse dieser Studie.

***Wer organisiert und finanziert die Studien?***

Diese NAFLD-Kohortenstudie wurde von der Medizinischen Klinik m. S. Hepatologie und Gastroenterologie einschl. Arbeitsbereich Stoffwechselerkrankungen, Charité-Universitätsmedizin Berlin, Campus Charité Mitte und Campus Virchow-Klinikum, konzipiert und wird von Dritten nicht finanziert (eine sog. IIT-Studie, **I**nvestigator **I**nitiated **T**rial: eine von Wissenschaftlern, Universitäten oder Studienzentren initiierte Studie ohne kommerzielles Interesse).

**Weitergabe von Informationen außerhalb des Studienzentrums**
Im Rahmen von Forschungskonsortien erhalten andere Wissenschaftler ggf. Zugang zu einem Teil Ihrer klinischen Daten und Proben – soweit dies für die Entwicklung der neuen Tests erforderlich ist. Alle Informationen sind durch einen Code pseudonymisiert. Dieser Code verbleibt am Studienzentrum und ermöglicht nur den Ärzten im Studienzentrum und dem ebenfalls der Schweigepflicht unterliegenden Studienpersonal eine Rückverfolgung.

Die Proben und Daten werden für folgende Zwecke eingesetzt

- Erforschung neuer nicht-invasiver Marker zur Identifikation des Stadiums Ihrer Lebererkrankungen
- Veröffentlichung von Daten und Verwendung für Ausbildungszwecke in anonymisierter Form
- Entwicklung zukünftiger wissenschaftlicher Fragestellungen hinsichtlich der Erforschung von Lebererkrankungen

***Angaben zum Datenschutz***
Die Verarbeitung Ihrer personenbezogenen Daten ist nur mit Ihrer Einwilligung rechtmäßig (Artikel 6 Datenschutz-Grundverordnung; DSGVO). Die Studienergebnisse sind gegen unbefugten Zugriff gesichert. Eine Entschlüsselung erfolgt nur unter den vom Gesetz vorgeschriebenen Voraussetzungen.

Die **Rechtsgrundlage** zur Verarbeitung der Sie betreffenden personenbezogenen Daten bilden bei klinischen Studien Ihre freiwillige schriftliche Einwilligung gemäß der Datenschutz-Grundverordnung (DSGVO) sowie der Deklaration von Helsinki (Erklärung des Weltärztebundes zu den ethischen Grundsätzen für die medizinische Forschung am Menschen) und der Leitlinie für Gute Klinische Praxis (GCP).

**Bezüglich Ihrer Daten haben Sie folgende Rechte:**
**Recht auf Auskunft:** Sie haben das Recht auf Auskunft über die Sie betreffenden personenbezogenen Daten, die im Rahmen der klinischen Studie erhoben, verarbeitet oder ggf. an Dritte übermittelt werden (Aushändigen einer Kopie) (Artikel 15 DSGVO).
**Recht auf Berichtigung:** Sie haben das Recht, Sie betreffende unrichtige personenbezogene Daten berichtigen zu lassen (Artikel 16 und 19 DSGVO).
**Recht auf Löschung:** Sie haben auch das Recht auf Löschung aller Sie betreffenden personenbezogener Daten, sofern dies möglich ist (z. B. wenn diese Daten für den Zweck, für den sie erhoben wurden, nicht mehr notwendig sind (Artikel 17 und 19 DSGVO)).
**Recht auf Einschränkung der Verarbeitung:** Unter bestimmten Voraussetzungen haben Sie das Recht, eine Einschränkung der Verarbeitung zu verlangen, d.h. die Daten dürfen nur gespeichert, nicht verarbeitet werden. Dies müssen Sie beantragen. Wenden Sie sich hierzu bitte an ihren Prüfer oder an den Datenschutzbeauftragten des Prüfzentrums (Artikel 18 und 19 DSGVO).
**Recht auf Datenübertragbarkeit:** Sie haben das Recht, die Sie betreffenden personenbezogenen Daten, die Sie dem Verantwortlichen für die klinische Studie/klinische Prüfung bereitgestellt haben, zu erhalten. Damit können Sie beantragen, dass diese Daten entweder Ihnen oder, soweit technisch möglich, einer anderen von Ihnen benannten Stelle übermittelt werden (Artikel 20 DSGVO).
**Widerspruchsrecht:** Sie haben das Recht, jederzeit gegen konkrete Entscheidungen oder Maßnahmen zur Verarbeitung der Sie betreffenden personenbezogenen Daten Widerspruch

einzulegen (Art 21 DSGVO, § 36 BDSG-neu). Eine solche Verarbeitung findet anschließend grundsätzlich nicht mehr statt.
**Einwilligung zur Verarbeitung personenbezogener Daten und Recht auf Widerruf dieser Einwilligung:** Die Verarbeitung ihrer personenbezogenen Daten ist nur mit Ihrer Einwilligung rechtmäßig (Artikel 6 DSGVO). Sie haben das Recht, Ihre Einwilligung zur Verarbeitung personenbezogener Daten jederzeit zu widerrufen und können weiterhin darüber entscheiden, ob die bereits erhobenen Daten gelöscht werden oder weiterverwendet werden dürfen (Artikel 7, Absatz 3 DSGVO).
Möchten Sie eines dieser **Rechte in Anspruch nehmen,** wenden Sie sich bitte an:

**Verantwortliche Studienärzte:**
PD Dr. med. Münevver Demir (Studienleiterin) und Prof. Dr. Frank Tacke (Vertreter)
Charité - Universitätsmedizin Berlin, Medizinische Klinik m. S. Hepatologie und Gastroenterologie einschl. Arbeitsbereich Stoffwechselerkrankungen, Campus Virchow-Klinikum, Augustenburger Platz 1, 13353 Berlin
Tel. + 49 30 450 66 53 37; Fax: +49 30 450 55 39 42

**Die Datenschutzbeauftragte des Verantwortlichen ist:**
Charité - Universitätsmedizin Berlin
Campus Charité Mitte
Stabsstelle Datenschutz
Charitéplatz 1
10117 Berlin
