# Supplementary material for: Immunologic effects of locoregional therapies for unresectable hepatocellular carcinoma
Source: JHEP Rep. 2025 Aug 22;7(12):101555. doi: 10.1016/j.jhepr.2025.101555 (PMC12637058; doi:10.1016/j.jhepr.2025.101555)
Supplement: Multimedia component 5 [file mmc5.pdf]

Immunologic effects of locoregional therapies for unresectable hepatocellular carcinoma

Robin Schmidt[1,2], Bernhard Gebauer[1], Nilufar Akbari[3], Christoph Roderburg[4], Giovanni Federico Torsello[5], Uli Fehrenbach[1], Timo Alexander Auer[1,6], Raphael Mohr[7], Gracia Lana Ardila Pardo[1], Winna Lim[1], Fabio Pivetta[1], Elif Can[1], Charlie Alexander Hamm[1,6], Frank Tacke[7], Bernd Hamm[1], Linda Hammerich[7], Lynn Jeanette Savic[1,2,6,*]

JHEP Reports **2025**. vol. 7 | 1–12

Check for updates

Background & Aims: The combination of locoregional therapies (LRT) with immune checkpoint inhibitors (ICIs) in unresectable hepatocellular carcinoma (HCC) is expected to enhance immune-mediated anti-tumor effects. Although clinical trials are underway, an unmet need exists to understand the immunological effects of LRT and how they evolve. This study aimed to longitudinally assess immune cell subpopulations and checkpoint expression after LRT.

Methods: This prospective, single-center study (DRKS00026994) enrolled 128 consecutive patients with unresectable HCC, who underwent conventional transarterial chemoembolization (cTACE), interstitial high-dose-rate brachytherapy (iBT), or a combination of cTACE and iBT (from July 2020 to September 2021). Peripheral blood samples were collected at baseline, 1 day after LRT, and 2 months after LRT. Immune cells were quantified using spectral flow cytometry. Immune cell subpopulations and checkpoint molecule expression were compared longitudinally and among treatment groups. Cluster analyses were used to explore immune profiles and their relationship with treatment response.

Results: Changes in absolute immune cell counts were detected 1 day after LRT, which largely diminished by 2 months. Myeloid populations increased significantly, whereas most lymphoid cells decreased after LRT. However, relative proportions of anti-tumoral $CD56^{diminished}$ NK cells (Cohen's D = 0.40, 95% CI 0.19–0.61, p <0.01), $CD8^{+}$ T cells (Cohen's D = 0.15, 95% CI -0.06 to 0.35, p = 0.01), and CTLA-4 expression on T cells ($CD4^{+}$: Cohen's D = 0.54, 95% CI 0.33–0.75, p <0.01; $CD8^{+}$: Cohen's D = 0.15, 95% CI 0.36–0.78, p <0.01) were upregulated at 1 day, particularly after cTACE. Cluster analysis distinguished responders from non-responders based on distinct immune profiles.

Conclusions: LRT induce an early pro-inflammatory immune response with increased myeloid, CTLA-4^{+} T cells, and cytotoxic lymphocytes, particularly after cTACE. These findings support the potential of immune profiling to guide personalized combination strategies with LRT and systemic immunotherapies.

Clinical trial number: DRKS00026994 (https://drks.de/search/de/trial/DRKS00026994).

© 2025 The Authors. Published by Elsevier B.V. on behalf of European Association for the Study of the Liver (EASL). This is an open access article under the CC BY license (http://creativecommons.org/licenses/by/4.0/).

Introduction

Hepatocellular carcinoma (HCC) is the most common primary liver cancer and the third leading cause of cancer-related death worldwide.[1] In unresectable HCC, image-guided locoregional therapies (LRT) are guideline-approved first-line therapies in early- and intermediate-stage HCC, achieving good local tumor control with limited adverse events.[2] Of these, conventional transarterial chemoembolization (cTACE) is considered the standard of care for intermediate-stage HCC (Barcelona Clinic Liver Cancer [BCLC] B), although there is substantial evidence for percutaneous ablation in early-stage HCC (BCLC A).[3] In contrast to thermal ablation techniques, the therapeutic efficacy of interstitial CT-guided high-dose-rate brachytherapy (iBT) is not limited by tumor size or heat dissipation. Therefore, iBT can also be used to treat larger tumors in the vicinity of vessels or other thermosensitive structures.[4,5] In addition, iBT can be combined with cTACE to treat hypervascularized, large HCC.[6,7]

HCC tumorigenesis is mainly driven by underlying chronic liver diseases, which are hypothesized to generate an immunosuppressive tumorigenic milieu, leading to the characteristic metachronous and multicentric occurrence of HCC.[8] Novel systemic immunotherapies aim at lowering the barrier of immunosuppression while restoring the resources of the patients' immune system to overcome cancer immune evasion.[9] Among these therapies, immune checkpoint inhibitors (ICIs)

* Corresponding author. Address: Department of Radiology, Charité–Universitätsmedizin Berlin, Augustenburger Platz 1, 13353 Berlin, Germany. Tel.: +49 30 450 657 093; fax: +49 30 450 755 794 7.
E-mail address: lynn-jeanette.savic@charite.de (L.J. Savic).
https://doi.org/10.1016/j.jhepr.2025.101555

are the most studied tools and have already been included in the first-line treatment of advanced-stage HCC (BCLC C) following the IMbrave-150 (atezolizumab/bevacizumab) and HIMALAYA trials (durvalumab/tremelimumab).[10,11] However, the response to ICIs in HCC is inferior to that of other cancer entities and varies among patients, and therapy-limiting adverse events occur frequently, calling for further strategies to improve tumor susceptibility.

In this regard, ongoing phase III trials are investigating the synergistic potential of ablative and embolic LRT combined with systemic therapies to improve tumor response and patient survival in different disease stages.[12–15] To date, available results from such trials are promising, demonstrating prolonged recurrence-free survival in patients with early HCC undergoing ablation or resection (IMbrave-050, NCT04102098, although not sustained in the long-term follow-up[16]), or intermediate-stage HCC undergoing cTACE (EMERALD-1 trial, NCT03778957) combined with systemic therapies.[17,18] The underlying rationale for combining LRT with ICI is based on the hypothesis that LRT induce unregulated, unprogrammed immunogenic cell death. Consequently, cell debris, including tumor-associated antigens, is presented via antigen-presenting cells, which subsequently activate $CD8^+$ T cell response.[19–24] In addition, with increasing evidence supporting the importance of hydrophobicity in immune system activation, Lipiodol®-based cTACE may be a favorable modality for this endeavor.[25–27]

Although this commonly cited rationale is reasonable,[28] the biophysiological mechanisms of LRT-induced immunological effects and their temporal dynamics are not yet well understood or characterized.[29] An improved understanding of the systemic immune response induced by LRT may help exploit their potential as conditioning tools to convert immune-resistant tumor habitats toward a more susceptible tumor microenvironment that could be targeted with ICIs even in earlier disease stages.

Therefore, this study aims to longitudinally quantify the absolute cell counts and relative proportions of peripheral immune cell subpopulations and their functional status in patients with unresectable HCC following different ablative and embolic LRT, including cTACE, iBT, and cTACE/iBT.

Materials and methods

Study design and participants

Patients with a primary diagnosis of unresectable HCC and the multidisciplinary tumor board consensus recommendation for LRT were consecutively recruited at the *Charité—University Medicine Berlin* between July 29, 2020, and September 15, 2021, as part of this prospective, single-center, investigator-initiated observational clinical trial (DRKS00026994). LRT comprised cTACE or iBT or a combination of both in curative or palliative intent or for bridging to transplant. Moreover, eligible patients had to have preserved liver function (Child–Pugh A or B), an Eastern Cooperative Oncology Group of 0 or 1, no systemic treatment or prior LRT on the target lesion, and no contraindications for LRT, such as decompensated liver cirrhosis with ascites, coagulopathy, thrombocytopenia, and incompliance (Fig. 1). Cross-sectional, peri-interventional imaging, including MRI and CT, was obtained using standardized protocols as described below. Peripheral blood sampling for flow cytometry analysis was performed at baseline, 1 day after LRT, and 2 months after LRT, along with routine diagnostic blood sampling to avoid additional patient harm. For patients who received cTACE/iBT, blood samples were obtained 1 day after iBT.

Study objectives

The primary endpoint of this study was to provide longitudinal immunological profiles of peripheral blood samples and the immune cells' functional status at baseline, 1 day after LRT, and 2 months after LRT to characterize LRT-specific immune cell alterations following ablation and or embolization. Next, patients were stratified according to their type of treatment (iBT, cTACE, or cTACE/iBT) to compare the immunologic response among their different mechanisms. Secondary endpoints included the prediction of response to therapy using cluster analysis based on lymphoid and myeloid immune cell counts and their functional molecules, as well as the correlation of baseline immune profiles and immune cell alterations 1 day after LRT with clinical, disease, and laboratory parameters.

Immunostaining and FACS

The detailed staining protocol is described in the Supplementary material. Briefly, samples were analyzed using a Cytek® Aurora flow cytometer (Cytek Biosciences, Fremont, CA, USA). Unmixing and spillover correction were performed using SpectroFlo software (Cytek Biosciences). Data were analyzed using FCS Express V7 (De Novo Software, Pasadena, CA, USA) to assess $CD45^+$ lymphoid and myeloid immune cell populations.[30] The gating strategy is shown in Fig. S1 and described in the Supplementary material. Briefly, cells were grouped into three morphological subsets: (1) T cell responses, (2) antigen-presenting cells, and (3) inflammatory and phagocytic cells. Checkpoint molecules and exhaustion markers were assessed on these subpopulations (Fig. S2).

Multiplex cytokine and chemokine assay analyses

Serum samples from patients undergoing cTACE were collected at baseline and 1 day after cTACE and analyzed for pro- and anti-inflammatory cytokines and chemokines using V-PLEX assay plates from Meso Scale Discovery (MSD, Rockville, MD, USA). The target antigens were immobilized on individual carbon spots within the wells, where diluted serum samples were applied. After washing, the plates were processed using the MSD QuickPlex instrument, which generates electrical currents to induce chemiluminescent reactions. The emitted light is captured by the device's camera and lens system, and a calibrator system adjusts the signals to calculate the minimum detectable concentrations based on international reference standards.

Completed and sequential treatment cycles

The standardized protocols of cTACE and iBT are described in the Supplementary material. A treatment cycle was considered completed when all target lesions defined at baseline were completely irradiated with the target dose of 20 Gy during iBT (both following iBT and cTACE/iBT) or when Lipiodol® was distributed within the whole tumor mass during cTACE. To achieve completed treatment cycles, some patients underwent

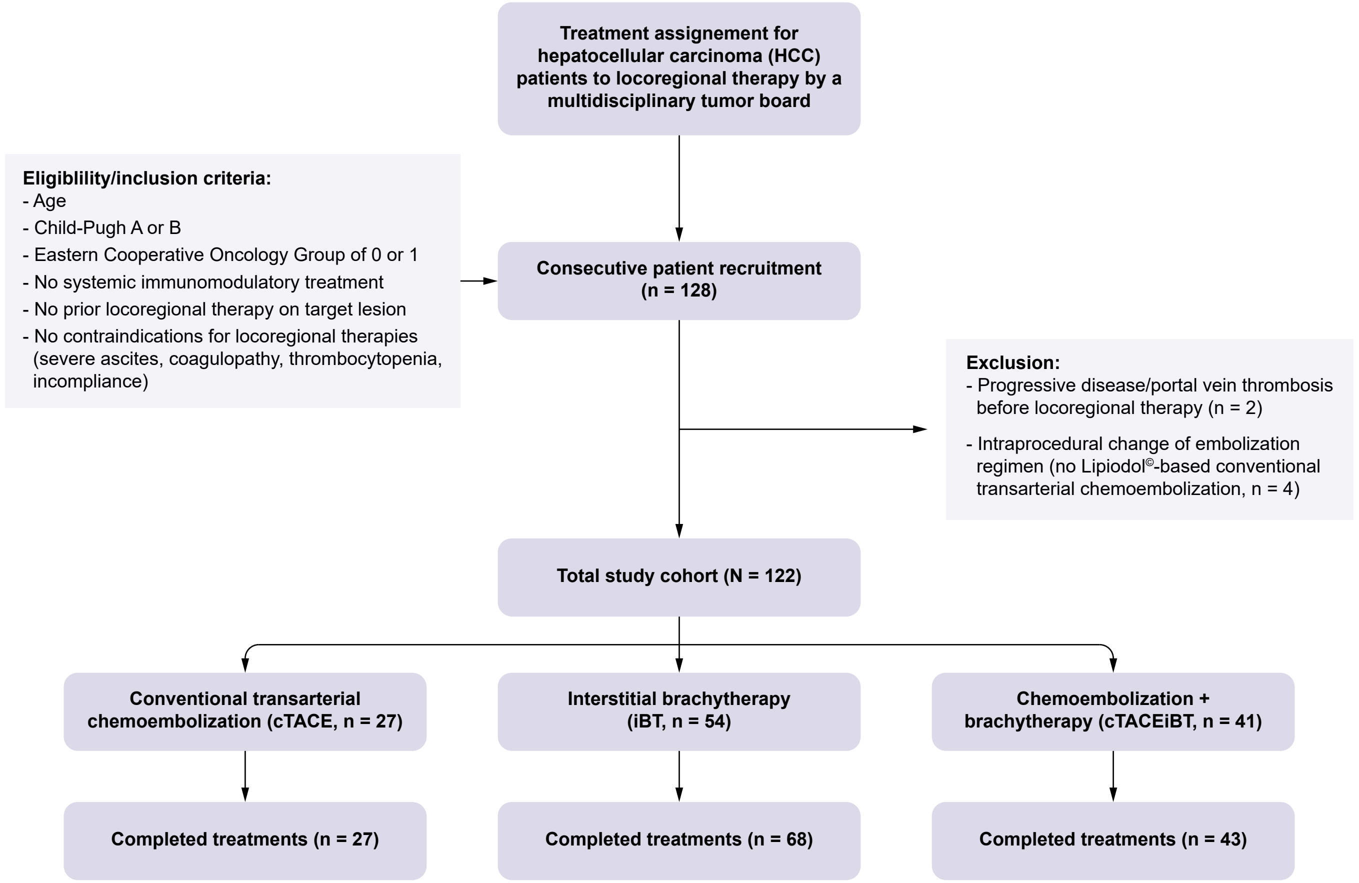

Fig. 1. Study cohort flowchart.

sequential treatments to avoid adverse effects from tumor lysis or to reduce cumulative puncture risk when the patient had multifocal or large lesions at baseline that could not be addressed within one session. If a patient developed a new target lesion during follow-up that was not present at baseline, and LRT was recommended by the multidisciplinary tumor board and performed for these new lesions within the recruitment time frame, the respective independent treatment cycles were considered separate, recorded in the study cohort, and followed the same study protocol as previously described.

Imaging time points and image analysis

Baseline multiparametric MRI was acquired within 30 days before the first completed treatment cycle, and follow-up MRI scans were obtained at approximately 2 months after each completed cycle, then every 3 months for the first year, and then every 6 months for the following years. CT imaging without the use of contrast media was performed 1 day after cTACE for the assessment of Lipiodol® distribution and could be used for iBT planning in the cTACE/iBT group. Details on image acquisition and protocols are provided in the Supplementary material.

Tumor response was assessed after each completed treatment cycle at each available follow-up time point within the first year using the Response Evaluation Criteria In Solid Tumors (RECIST) 1.1, the modified (m)RECIST criteria, and the Liver Imaging and Data Reporting System (LI-RADS) Treatment Response Algorithm (TRA) v2017, in consensus by two radiologists with 3 and 8 years of experience in abdominal imaging, who did not allocate or perform LRT.[31–33] Patients with complete or partial response (RECIST or mRECIST) or LI-RADS TRA non-viable were considered responders. In contrast, the group of non-responders consisted of patients with stable or progressive disease (RECIST or mRECIST) or LI-RADS TRA viable and LI-RADS TRA equivocal. Images were viewed, and calculations were performed using Visage Picture Archiving and Communication Systems (PACS) client version 7 (Visage Imaging, EU HQ, Berlin, Germany).

Statistics

Sample size calculation

A statistical *a priori* sample size calculation was performed using GPower 3.1 (Heinrich-Heine-Universität, Düsseldorf, Germany). Effect sizes for alterations in T cells, B cells, NK cells, monocytes, granulocytes, and classical dendritic cells (cDCs) were estimated for each treatment group using data from 47 consecutively enrolled patients during active recruitment. Parameters for high probability (1 - β, 80% power) and detection of a significant change (α, 5%) were set, and minimum sample sizes were calculated for each of the three treatment groups. Among these, the most homogeneous

values were observed in the population of B cells (effect sizes for patients following cTACE = 0.28, iBT = 0.14, and cTACE/iBT = 0.19), which was chosen as a reference parameter for the estimation of the overall cohort's sample size. The minimum estimated sample size was the lowest in patients treated with transarterial chemoembolization (TACE), given that the largest effects were observed in this group in the preliminary analysis. Assuming a drop-off rate of 5%, the total sample size was 128 patients to be enrolled.

Statistical analysis

The evaluation was explorative and descriptive. Baseline characteristics were analyzed. For continuous variables, descriptive statistics included the mean, standard deviation, median, first and third quartiles, minimum, and maximum values. For categorical variables, statistics included absolute and relative frequencies. The focus of the study's analysis was to estimate effect sizes. Because of the exploratory study design, p values (if calculated) do not enable confirmatory conclusions. Statistical analyses were performed using R software v4.3 (R Foundation for Statistical Analysis, Vienna, Austria).

LRT-specific effects on immune alterations

Cohen's D effect sizes of immune cell alterations were calculated between baseline and 1 day after LRT, and between 1 day after LRT and 2 months after LRT. In addition, Cohen's D effect sizes of the cytokine/chemokine alterations were calculated between baseline and 1 day after cTACE only. Heatmaps were generated to summarize longitudinal immune cell changes and compare them among treatment groups using the median of standardized rank-transformed parameters. Taking outliers and partially skewed data into account, all parameters were rank-transformed, ranging from 0 to 1.

Cluster analysis and effects of immune profiles on treatment response

Patients were stratified into treatment responders versus non-responders according to radiological response assessed at 6 months after LRT. T-distributed stochastic neighbor embedding (t-SNE) was used for dimensionality reduction. Hierarchical cluster analysis was performed to identify immune profiles. The choice of hyperparameters was based on previous publications.[34] Different cluster analyses containing the cube root-transformed immune parameters were conducted using major baseline immune cell counts as well as the immune alterations of T cells, B cells, NK cells, monocytes, granulocytes, and cDCs, as well as PD1$^+$, PDL-1$^+$, and CTLA4$^+$-expressing CD4$^+$ and CD8$^+$ T cells to account for the impact of the most relevant ICI targets.

Subsequently, the clusters adjusted by immune cell alterations were further analyzed for differences in general immune profiles as well as the distribution of clinical parameters and treatment response. In addition, within a two-way linear correlation matrix, Spearman's correlation coefficient and linear regression analyses were performed to compare baseline immune parameters and dynamic immune alterations, respectively, with selected patients, disease, and laboratory parameters.

Results

Study cohort

A total of 128 consecutive patients were enrolled. Two patients experienced progressive disease and did not undergo LRT as planned. Four patients, who were originally scheduled to undergo cTACE, were excluded from the analysis because of a intraprocedural decision to perform TACE with degradable starch microspheres (DSM-TACE) instead. Ultimately, 122 consecutive enrolled patients receiving 183 separate treatments, including 138 completed treatment cycles for the treatment of 171 target lesions, were considered as the final study cohort. Characterizing completed treatment cycles, 27 patients were treated with cTACE, 54 patients were treated with iBT, and 41 with cTACE/iBT (Fig. 1). Specifically, 15 patients treated with cTACE (55.6%), three with iBT (5.6%), and seven with cTACE/iBT (17.1%) received sequential treatment cycles to achieve treatment completion. Meanwhile, 14 patients treated with iBT (25.9%) and two with cTACE/iBT (4.9%) received separate completed treatments targeting individual lesions that occurred metachronously during active study recruitment. Most patients had BCLC A disease when scheduled for ablation and TACE, as they were being bridged to transplantation. Patients, tumor, and disease characteristics are summarized in Table 1.

Immune cell subpopulations over time

In the entire study cohort, the absolute lymphoid cell numbers decreased 1 day after LRT (Cohen's D = -0.48, 95% CI -0.69 to -0.26, p <0.01) and increased again by 2 months (Cohen's D = 0.43, 95% CI 0.19–0.44, p <0.01). In contrast, the myeloid cell counts largely increased 1 day after LRT (Cohen's D = 0.73, 95% CI 0.51–0.95, p <0.01) and decreased by 2 months (Cohen's D = -0.67, 95% CI -0.91 to -0.44, p <0.01). Overall, most cell counts at 2 months after LRT were comparable to those at baseline (Fig. 2).

Specifically, regarding T cell response, the absolute counts of CD4$^+$ T helper cells decreased the most 1 day after LRT (Cohen's D = -0.56, 95% CI -0.77 to -0.34, p <0.01). Among antigen-presenting cells, classical monocytes increased the most 1 day after LRT (Cohen's D = 0.39, 95% CI 0.17–0.61, p <0.01). Of all inflammatory and phagocytic cells, the absolute numbers of CD56^{bright} NK cells decreased the most 1 day after LRT (Cohen's D = -0.77, 95% CI -0.99 to -0.54, p <0.01), whereas neutrophils increased the most in absolute numbers (Cohen's D = 0.77, 95% CI 0.54–0.99, p <0.01).

With regard to the relative alterations of the major immune cell subpopulations, a relative increase in the proportions of CD8$^+$ T effector cells (p = 0.01), classical monocytes (p <0.01), type 1 cDCs (p = 0.13), and CD56^{diminished} NK cells (p <0.01) was observed. In contrast, a relative decrease in the proportions of CD4$^+$ T helper cells (p <0.01), non-classical monocytes (p <0.01), type 2 cDCs (p = 0.03), and CD56^{bright} NK cells (p <0.01) was observed 1 day after LRT (Fig. 3 and Table S1–S3).

Regarding the changes in cytokines and chemokines following cTACE, most humoral factors decreased 1 day after cTACE, including IL-13 (p = 0.03), IL-17 (p = 0.01), pro-inflammatory cytokines INFγ (p = 0.03) and TNFα (p = 0.11), chemokines MCP1 (p <0.01) and MIP1α (p <0.01), and the

Table 1. Baseline characteristics including patient, disease, and laboratory parameters.

Demographics	All	cTACE	iBT	cTACE/iBT
**Patient characteristics**				
Patients, n	122	27	54	41
Age (years), median [IQR]	70.0 [64.3, 77.8]	65.0 [57.5, 68.0]	71.0 [67.3, 78.0]	71.0 [65.0, 78.0]
Male/female, % (n)	78.7/21.3 (96/26)	74.1/25.9 (20/7)	87.0/13.0 (47/7)	70.7/29.3 (29/12)
**Disease characteristics**				
Cirrhosis (by imaging or histology), % (n)	95.1 (116)	92.6 (25)	96.3 (52)	95.1 (39)
Etiology of cirrhosis, % (n)				
Hepatitis B	12.1 (14)	20.0 (5)	9.6 (5)	10.3 (4)
Hepatitis C	16.3 (19)	12.0 (3)	15.4 (8)	20.5 (8)
Alcoholic-associated liver disease	41.4 (48)	40.0 (10)	44.2 (23)	38.5 (15)
MASLD	18.1 (21)	20.0 (5)	21.2 (11)	12.8 (5)
Others	12.1 (14)	8.0 (2)	9.6 (5)	17.9 (7)
CP class				
A, % (n)	92.2 (107)	61.1 (18)	100.0 (52)	94.9 (37)
B, % (n)	7.8 (9)	38.9 (7)	0.0 (0)	5.1 (2)
CP points, mean ± SD	5.3 ± 0.7	5.8 ± 1.1	5.1 ± 0.3	5.3 ± 0.5
MELD score, mean ± SD	9.7 ± 2.6	10.2 ± 2.8	9.8 ± 2.9	9.1 ± 2.2
**Target tumor characteristics**				
Completed treatments, n	138	27	68	43
Lesions per patient, mean ± SD	1.4 ± 0.6	1.4 ± 0.7	1.4 ± 0.6	1.3 ± 0.6
Unifocal/multifocal, % (n)	69.6/30.4 (96/42)	74.1/25.9 (20/7)	64.7/35.3 (44/24)	74.4/25.6 (32/11)
Index lesion size, median [IQR]	25.2 [17.2, 35.8]	22.9 [14.4, 30.0]	25.1 [17.2, 35.7]	28.0 [20.9, 51.3]
Barcelona Clinic Liver Cancer stage, % (n)				
A	90.6 (125)	70.4 (19)	100.0 (68)	88.4 (38)
B	9.4 (13)	29.6 (8)	0.0 (0)	11.6 (5)
**Laboratory parameters of liver function**				
Albumin (g/L), mean ± SD	39.4 ± 4.2	36.4 ± 3.0	40.1 ± 4.0	38.9 ± 4.4
Bilirubin (mg/dl), median [IQR]	0.75 [0.49, 1.13]	1.04 [0.69, 1.56]	0.70 [0.44, 0.95]	0.76 [0.49, 1.17]
ALT (U/L), median [IQR]	33.0 [24.0, 52.0]	40.0 [22.0, 63.0]	33.0 [26.8, 48.8]	29.0[21.0, 48.0]
AST (U/L), median [IQR]	45.0 [36.0, 60.0]	54.0 [39.5, 84.5]	44.5 [32.8, 42.3]	45.0 [36.0, 66.0]
γ-GT (U/L), median [IQR]	125.0 [63.0, 268.0]	89.0 [52.8, 218.3]	112.0 [66.8, 298.5]	142.0 [77.0, 217.0]
AP (U/L), median [IQR]	115.5 [85.3, 157.8]	121.0 [88.5, 149.0]	108.5 [79.0, 170.3]	125.0 [88.0, 156.5]
INR, median [IQR]	1.23 [1.13, 1.32]	1.22 [1.15, 1.31]	1.10 [1.05, 1.18]	1.11 [1.06, 1.17]

γ-GT gamma-glutamyl transferase; ALT, alanine aminotransferase; AP, alkaline phosphatase; AST, aspartate aminotransferase; CP, Child–Pugh; cTACE, conventional transarterial chemoembolization; iBT interstitial brachytherapy; MASLD, metabolic dysfunction-associated steatotic liver disease; MELD, model for end-stage liver disease.

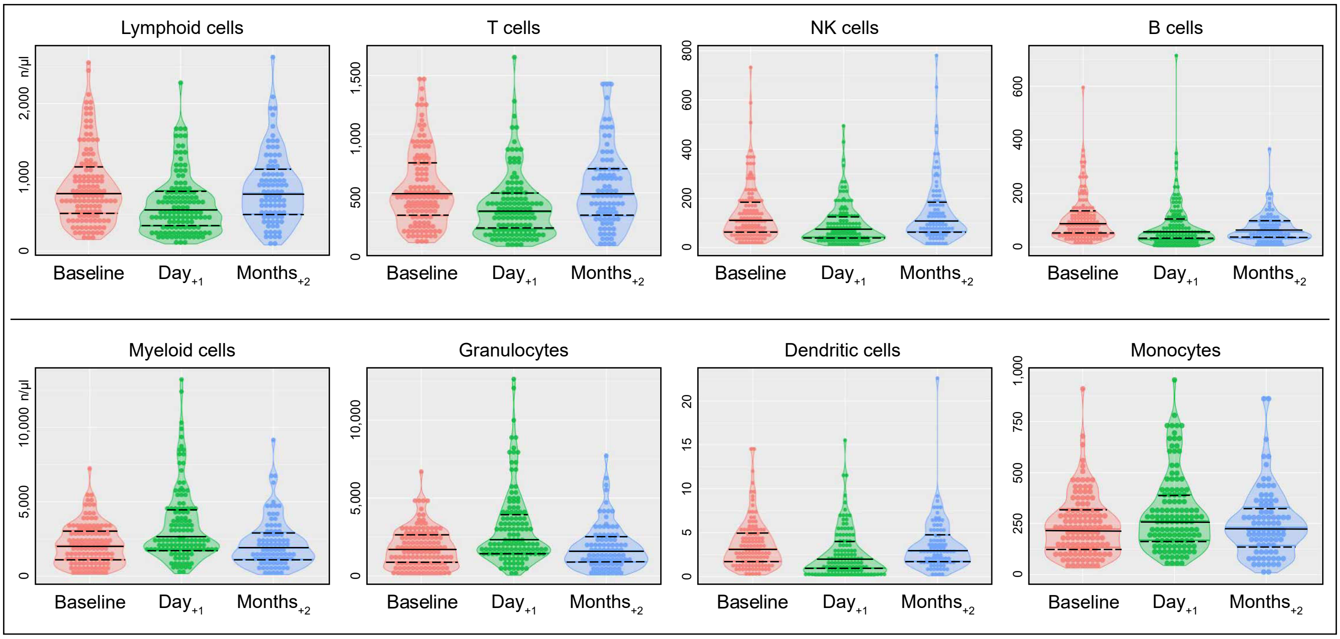

Fig. 2. Changes in absolute cell counts of immune cell populations following LRT. Violin plots depict absolute cell counts of major lymphoid (top row) and myeloid cell populations (bottom row). Data are depicted at baseline, 1 day after LRT, and 2 months after LRT. Although lymphoid cell counts tended to decrease 1 day after LRT and increase again 2 months after LRT, myeloid cell counts showed the opposite pattern. Solid lines indicate the median, and dashed lines show the first and third quartiles. Absolute cell counts of all immune cell populations are listed in Table S1. LRT, locoregional therapies.

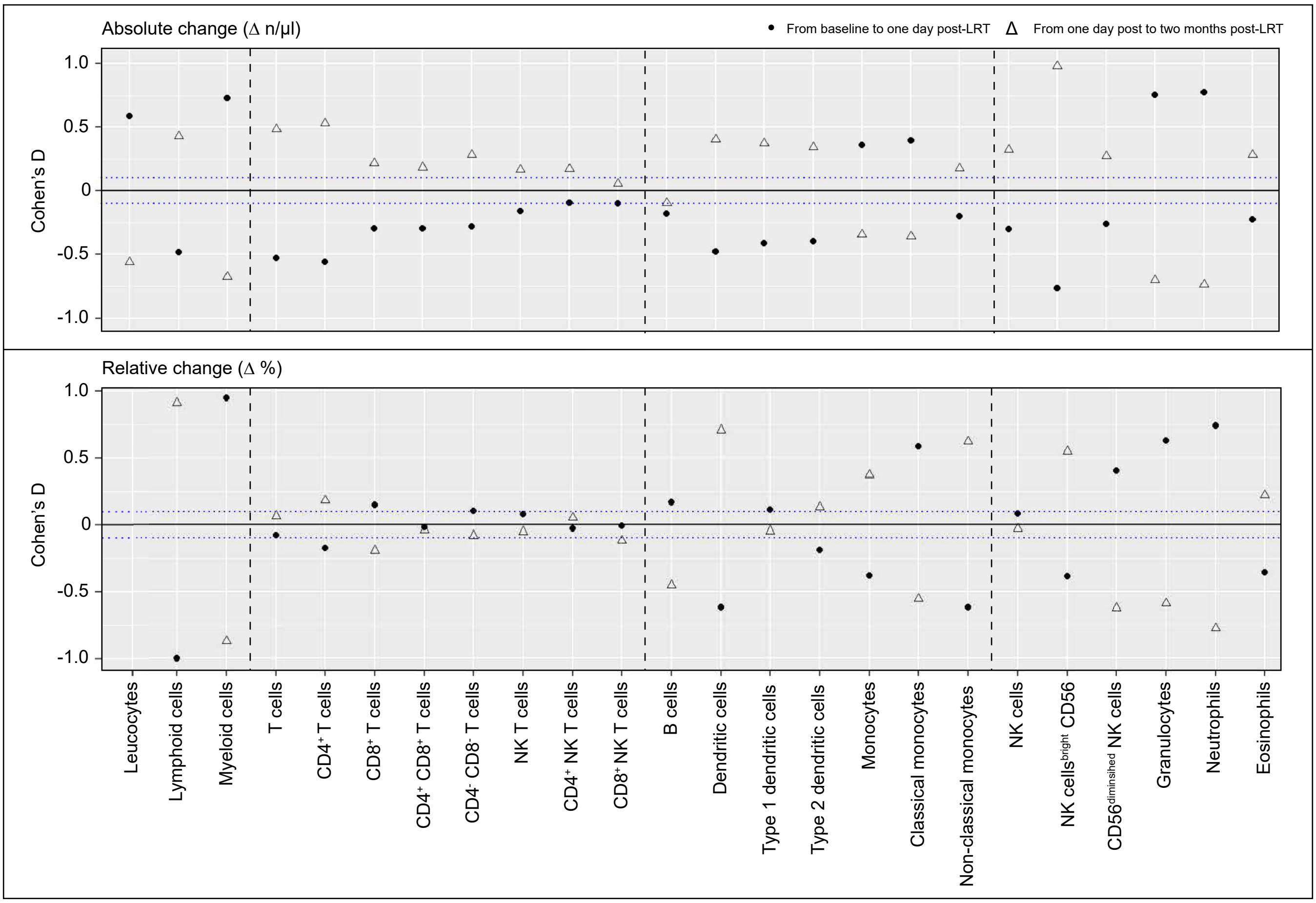

Fig. 3. Longitudinal changes in immune cell populations following LRT. Dot plots reveal the absolute (top row) and relative (bottom row) changes in immune cell populations after LRT. A relative shift in T cell, NK-cell, conventional dendritic cell, and monocyte subpopulations was observed 1 day after LRT, indicating an antitumoral and pro-inflammatory response that reverted 2 months after LRT. Filled dots show alterations between baseline and 1 day after LRT, and empty triangles show alterations between 1 day and 2 months after LRT. The blue dotted line indicates the cohort's median Cohen's D of ± 0.1. LRT, locoregional therapies.

angiogenesis factor VEGF (p <0.01). Only IL-1β showed a significant increase (p <0.01; Fig. S3 and Table S5). In contrast, several humoral factors decreased 1 day after TACE, including IL-4, IL-5, IL-8, IL-17, IFNγ, TNFα, MIP1α, MCP1, VEGF, and βFGF. Among these, only IL-13 (p = 0.03), IL-17 (p = 0.01), MIP1α (p = 0.01), MCP1 (p <0.01), and IFNγ (p = 0.03) showed statistically significant reductions (Table S4).

Immune cell subpopulations over time stratified by type of LRT

Baseline standardized medians of both lymphoid and myeloid cell counts were lower in patients following cTACE compared with patients following iBT or cTACE/iBT. The largest alterations in standardized medians of immune cell counts were also observed 1 day and 2 months after cTACE. Specifically, the standardized medians of T cells, non-classical monocytes, cDCs, CD56^{bright} NK cells, and eosinophils showed a larger decrease, and classical monocytes and neutrophils showed a larger increase 1 day after LRT compared with patients following iBT or cTACE/iBT. In addition, by 2 months after LRT, significantly higher standardized medians were observed for CD4^{+} T helper cells, CD4^{+} NK-T cells, B cells, and CD56^{bright} NK cells after cTACE compared with patients following iBT or cTACE/iBT (Fig. 4).

Functional immune cell markers over time

Similarly, the majority of activation markers expressing immune cells decreased 1 day after LRT. Only CTLA-4 expressing CD4^{+} and CD8^{+} T cells increased significantly in absolute numbers (Cohen's D = 0.54, 95% CI 0.33–0.75, p <0.01) and relative numbers (Cohen's D = 0.57, 95% CI 0.36–0.78, p <0.01). In addition, there was a distinct increase in classical monocytes expressing functional immune markers (*e.g.* CD1c, CTLA-4, HLA-DR, and TIM3). However, this increase was not confirmed in terms of the relative cell fractions. The absolute cell concentrations and effect sizes of the alterations at each time point are provided in Fig. 5 and Table S1–S3.

Functional immune cell markers over time stratified by type of LRT

Longitudinal changes in the expression of checkpoint, activation, and exhaustion molecules are provided in Fig. S4. Similarly, at baseline, patients following cTACE had comparable or

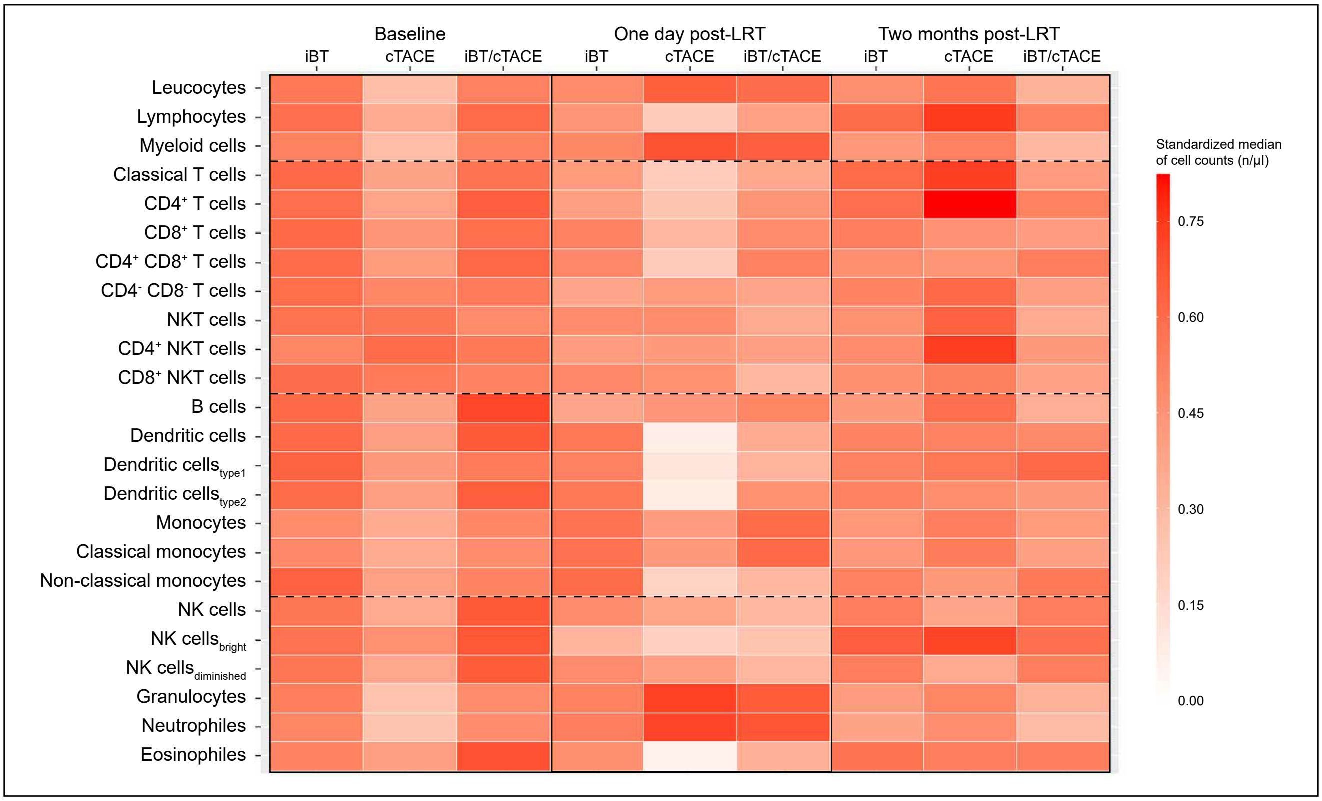

Fig. 4. Longitudinal changes in immune cell populations stratified by LRT type. Heatmap showing standardized medians (0–1) of immune cell counts at baseline and at 1 day and 2 months after iBT, cTACE, and combined cTACE/iBT. At baseline, patients in the cTACE group showed generally lower immune cell counts compared with the other groups. The strongest immunological effects of LRT were observed after cTACE, with standardized medians of immune cell populations decreasing substantially 1 day after LRT, followed by a larger increase 2 months after LRT. cTACE, conventional transarterial chemoembolization; iBT, interstitial brachytherapy; LRT, locoregional therapies.

lower standardized medians for each marker compared with patients following iBT and cTACE/iBT. However, 1 day after LRT, patients following cTACE demonstrated lower standardized medians of activation and exhaustion molecules on monocytes and B cells but higher CTLA-4 expression on both $CD4^+$ and $CD8^+$ T cells. In contrast, nearly no change was observed in CTLA-4 expression in patients following iBT. Moreover, following cTACE, higher standardized medians of $PD\text{-}1^+$, $CD38^+$, and $HLA\text{-}DR^+$ T cells and functional molecules on B cells were observed 2 months after LRT compared with patients following iBT or cTACE/iBT.

Associations of immune cell profiles with treatment response

Higher and lower myeloid cell counts were found in responders at both 2 and 6months after LRT. In addition, in responders according to LI-RADS TRA v2017, lymphoid cells steadily increased from baseline, over 1 day to 2 months after LRT. In non-responders, lymphoid cells showed a bidirectional trend, first decreasing 1 day after LRT and then increasing 2 months after LRT. Conversely, in responders according to LI-RADS TRA, myeloid cells largely increased 1 day after LRT, whereas in non-responders, they steadily increased from baseline, over 1 day to 2 months after LRT. These findings were most evident in patients following cTACE (Fig. S5). Overall treatment response according to RECIST, mRECIST, and LI-RADS TRA v2017 criteria is summarized in Table S5.

Immune profile clustering analysis

Overall, clustering analysis based on baseline immune cell counts revealed four clusters showing similar immunological profiles and no differences in treatment response at any follow-up interval. However, clustering analysis based on immune cell alterations revealed two more comprehensive clusters demonstrating different immunological profiles and differences in treatment response. The first cluster includes patients with a trend of decreasing T cell, B cell, NK-cell, monocyte, and cDC counts, whereas the second cluster included patients with no trends in lymphoid populations but largely increasing monocyte and granulocyte counts (Fig. 6). Regarding treatment response to LI-RADS TRA v2017, patients in the first cluster had higher rates of tumor viability at any follow-up interval (cluster 1 *vs*. cluster 2: first follow-up 43.5% *vs*. 24.4%, second follow-up 55.3% *vs*. 26.3%, and third follow-up 48.6% *vs*. 30.0%). In contrast, patients in the second cluster had higher rates of equivocal tumor response at any follow-up interval (cluster 1 *vs*. cluster 2: first follow-up 30.6% *vs*. 46.7%, second follow-up 12.8% *vs*. 36.8%, and third follow-up 13.5% *vs*. 16.7%) (Table 2). Probabilities for LI-RADS TRA non-viable did not differ between clusters and follow-up intervals. Additional cluster analysis, including the immune cell parameters of $PD1^+$,

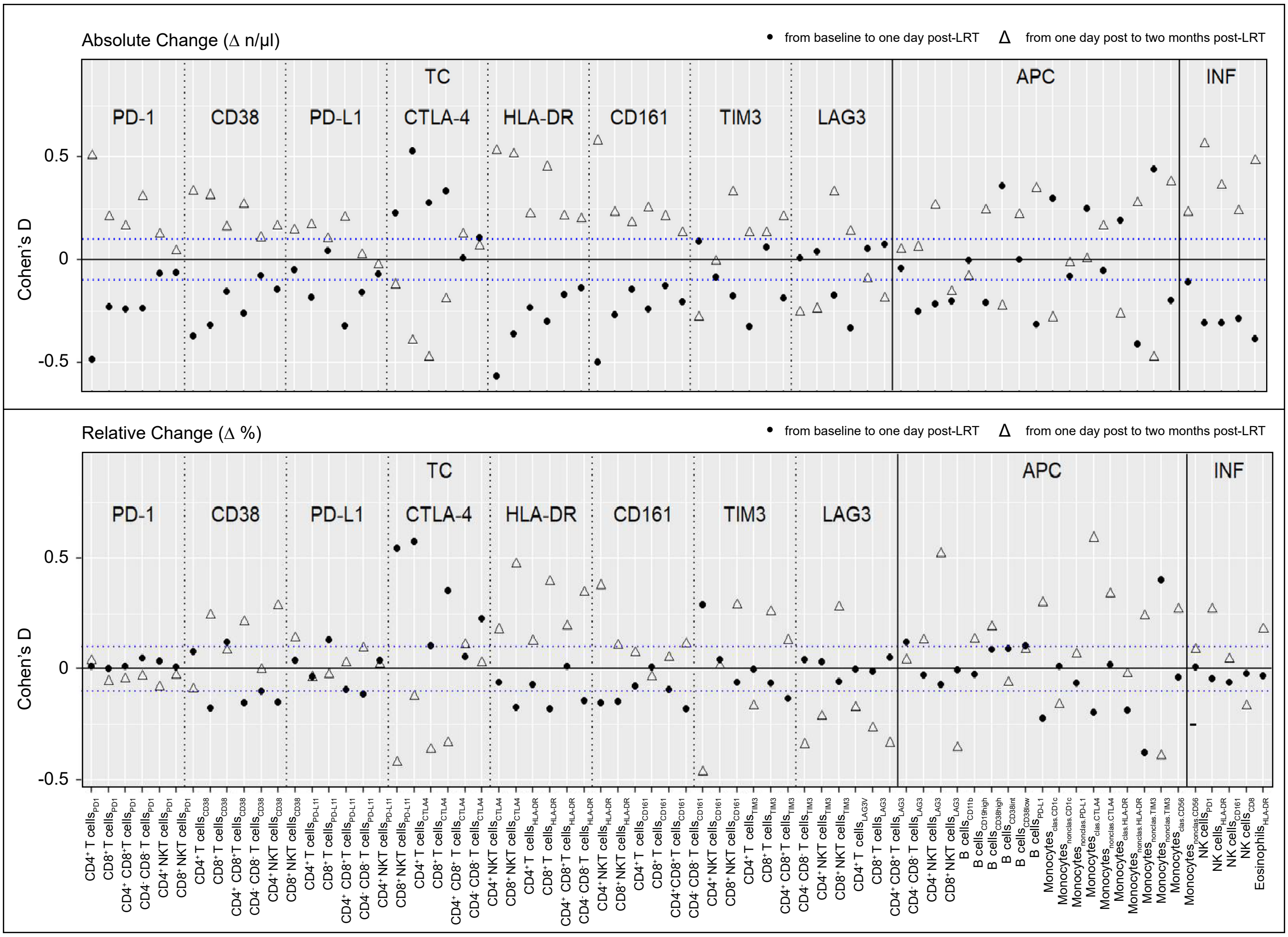

Fig. 5. Longitudinal changes in activation, exhaustion, and checkpoint molecules on immune cell populations following LRT. Dot plots reveal the absolute (top row) and relative (bottom row) alterations in checkpoints, activation, and exhaustion molecules after LRT. Although most cell counts and proportions decreased 1 day after LRT and increased again 2 months after LRT, opposite trends were observed for CTLA-4 and LAG3 expressions on T cells. Filled dots show alterations between baseline and 1 day after LRT, and empty triangles show the alterations between 1 day and 2 months after LRT. The blue dotted line shows the cohort's median Cohen's D of ± 0.1. LRT, locoregional therapies.

PD-L1$^+$, and CTLA4$^+$-expressing CD4$^+$ and CD8$^+$ T cells, did not reveal any different trends (Fig. S6 and Table S6).

Correlation of immune cell counts with patient, disease, and laboratory features

Spearman's correlation matrix analysis revealed a stronger correlation between baseline lymphoid cell counts and patients, disease, and laboratory values than myeloid cell counts. Particularly, the Child–Pugh and MELD scores showed a negative correlation with T cell counts (Child–Pugh: r_s = -0.29; MELD score: r_s = -0.37) and B-cell counts (Child–Pugh: r_s = -0.18, MELD score: r_s = -0.25). In contrast, only cDCs showed a positive, conclusive correlation with the BCLC stage. Regarding laboratory parameters, positive correlations were found for immune cell counts with albumin and gamma-glutamyl transferase levels, whereas negative correlations were observed for most immune cell counts with C-reactive protein, bilirubin, urea, and creatinine (Fig. S7). Linear regression analysis revealed BCLC stages B and C (compared with BCLC stage A) as the strongest predictors of immune cell alterations, namely, decreased dendritic cells and increased granulocytes (Fig. S8).

Discussion

This prospective observational study analyzed immune cell dynamics in 122 patients with unresectable HCC undergoing 138 LRT. Longitudinal flow cytometry of peripheral blood revealed transient but distinct immunomodulatory effects after LRT, with significant changes observed 1 day after LRT that largely diminished by 2 months.

One day after LRT, a predominantly pro-inflammatory response was observed, characterized by increased myeloid cell counts and decreased lymphoid populations across all treatment modalities. In addition, immune checkpoint molecules were measured, as they are common targets of immunotherapies and are currently being exploited in ongoing clinical trials alongside LRT.[28,35,36] In this context, cTACE induced a relative increase in CD8$^+$ T cells and CTLA-4-expressing CD4$^+$ and CD8$^+$ T cells, suggesting early T cell activation followed by potential immune regulatory feedback.

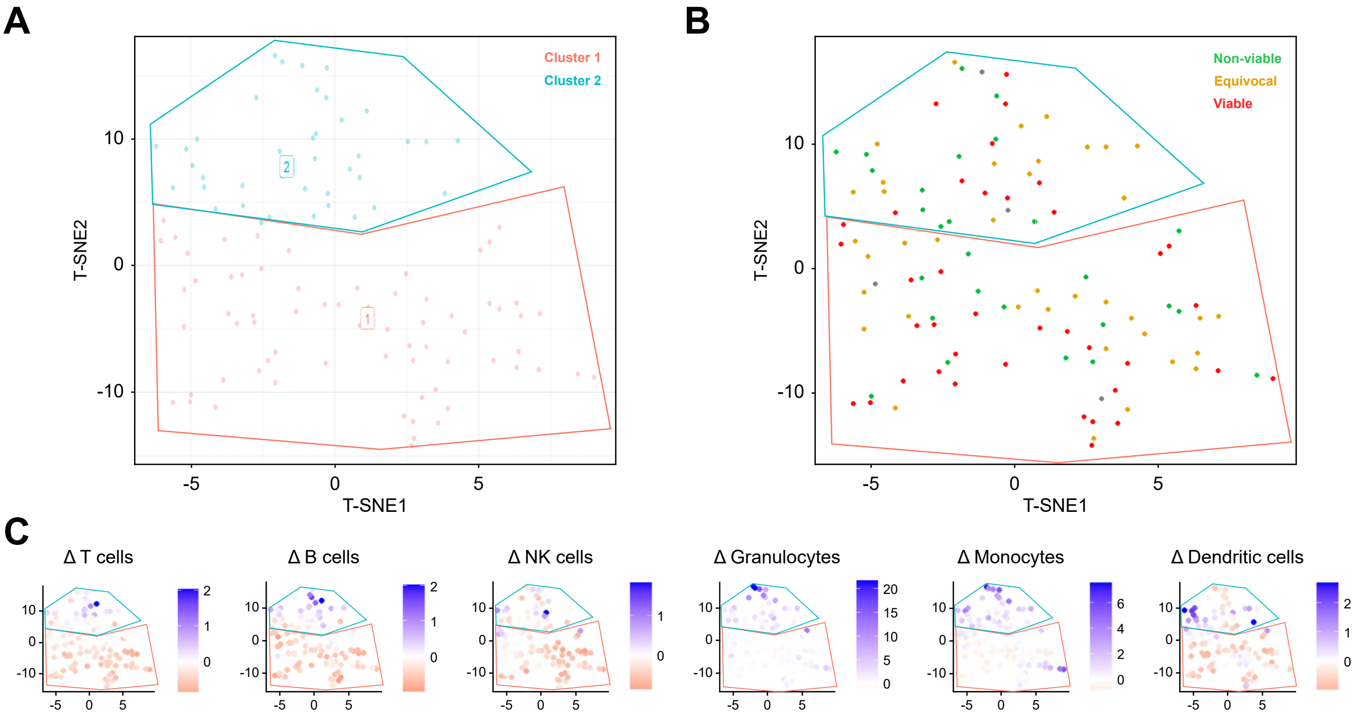

Fig. 6. Associations of immune cell dynamics with tumor response. (A) T-SNE clustering analysis based on immune cell alterations from baseline to 1 day after LRT revealed two clusters of patients with different immunological responses. (B) Patients in the first cluster showed higher rates of remaining tumor viability according to LI-RADS TRA v2017, whereas patients in the second cluster demonstrated more inflamed tumor response with higher rates of equivocal tumor response. (C) Patients in the first cluster showed decreasing populations of T cells, B cells, NK cells, monocytes, and conventional dendritic cells, whereas patients in the second cluster showed no trends in lymphoid populations but an increase in conventional dendritic cells, monocytes, and granulocytes. LI-RADS TRA, Liver Imaging and Data Reporting System Treatment Response Algorithm; LRT, locoregional therapies; t-SNE, T-distributed stochastic neighbor embedding.

Table 2. Immune cell alterations from baseline to 1 day after LRT and treatment responses according to LI-RADS TRA v2017, based on immune clustering revealed by t-SNE analysis.

	Cluster 1 (n = 64) Cohen's D (95% CI)	Cluster 2 (n = 47) Cohen's D (95% CI)
**Immune cell population**		
B cells	-0.45 (-0.65 to -0.29)	0.06 (-0.25 to 0.45)
T cells	-0.44 (-0.56 to -0.34)	-0.03 (-0.17 to 0.22)
NK cells	-0.42 (-0.62 to -0.28)	-0.05 (-0.24 to 0.29)
Monocytes	-0.18 (-0.48 to 0.30)	0.91 (0.6 to 2.09)
Granulocytes	0.23 (-0.36 to 0.94)	1.70 (0.30 to 4.73)
cDCs	-0.57 (-0.84 to -0.29)	0.26 (-0.18 to 1.04)
**LI-RADS TRA v2017 6 months after LRT, n (%)**		
Non-viable	15 (31.9)	14 (36.8)
Equivocal	6 (12.8)	14 (36.8)
Viable	26 (55.3)	10 (26.3)

cDC, classical dendritic cell; LI-RADS TRA, Liver Imaging and Data Reporting System Treatment Response Algorithm; LRT, locoregional therapies; t-SNE, T-distributed stochastic neighbor embedding.

Previous studies have investigated certain cell populations or cytokines as surrogates of a pro-inflammatory immune response in the context of LRT. Erinjeri *et al.*[37] reported significant increases in IL-6 and IL-10 plasma levels after thermal ablation compared with baseline, which varied depending on the treatment modality and tumor type, respectively. IL-6 may mediate immune cell invasion by promoting the activation and recruitment of cytotoxic T cells.[37]

Cytokine analysis after TACE in the ImmuMITT cohort revealed elevated IL-1β and IL-6 (pro-inflammatory, neutrophil-recruiting), IL-2 (regulatory T cell [Treg]- and effector T cell-recruiting), and IL-10 (immunomodulatory), likely driven by anitgen prensenting cells (APC)-mediated processing of tumor-associated antigens released after embolization.[15] In contrast, levels of IL-4, IL-5, IL-8, IL-17, INFγ, TNFα, MIP1α, MCP1, VEGF, and βFGF declined, with VEGF/βFGF reduction potentially indicating decreased pro-angiogenic signaling. Given that VEGF/FGF-mediated angiogenesis functions as a 'vascular barrier' impeding immune cell infiltration (angiogenic immune evasion), these findings suggest a shift in the tumor microenvironment favoring immune infiltration.[38] VEGF also modulates immune function by inhibiting dendritic cell maturation, promoting Tregs, and enhancing myeloid-derived suppressor cells, further linking angiogenesis to immune regulation.[17] cTACE triggers rapid necrosis through ischemia and chemotherapy-induced damage, promoting a robust inflammatory response.[39,40]

In contrast, iBT induces tumor cell death gradually via radiation, leading to mitotic catastrophe and apoptosis, followed by an early monocyte response without acute inflammation.[41] Although radiation has been described as immunogenic, the delayed onset of its effects raises questions about the optimal timing for ICI combination. Additionally, apoptotic clearance by macrophages and dendritic cells may limit early inflammatory responses after iBT.[42] Notably, cTACE alone induced stronger immune alterations than the cTACE/iBT combination, suggesting no additional immune benefit of combining embolization with brachytherapy.

CD8$^+$ T cells, classical monocytes, type 1 cDCs, and CD56^{diminished} NK cells increased after LRT, reflecting an activated immune response. However, the simultaneous upregulation of CTLA-4 on CD4$^+$ and CD8$^+$ T cells 1 day after cTACE suggests a negative feedback mechanism to regulate excessive activation and prevent clonal overexpansion.[43] Given the transient nature of these changes, initiating ICI therapy soon after LRT may optimize synergistic efficacy.

In contrast to T cell activation, checkpoint and exhaustion marker levels on B cells decreased. Because radiation can lead to lymphodepletion, the observed T cell decline after LRT may reflect their migration to lymph nodes for expansion before returning to the tumor site, a process supported by preclinical animal models.[44,45] Although lymphocytopenia is generally associated with poor outcomes in patients with cancer receiving radiotherapy,[46] studies on stereotactic body radiation therapy combined with ICI have shown increased CD8$^+$ T cells after treatment.[47] However, recent data suggest that CD8$^+$ T cells may be less radiosensitive than B cells or CD4$^+$ T cells, suggesting dose-dependent immunomodulation after radiotherapy.[48] As iBT delivers significantly higher radiation doses than stereotactic body radiation therapy, results from this study are potentially challenging previous observations while acknowledging that the predefined time points may not fully capture gradual changes after iBT.

In this study, only a subset of immune parameters remained sustainably elevated in patients following cTACE. This subset included CD4$^+$ helper T cells, NK-T cells, CD56^{bright} NK cells, and expression levels of PD-1, CD38, and HLA-DR on T cell populations. Although several studies have also shown increasing peripheral CD4$^+$ T helper cells and Tregs and increasing PD-1 expression levels on Tregs after cTACE, it remains unclear whether these alterations represent an impaired response to residual tumor cells or an immunological state of surveillance and regulation.[49–51] Early evidence suggests a link between the upregulation of Tregs and the expression of inhibitory molecules such as PD-1 and CTLA-4 after cTACE, which may exert tumor-mediated immunosuppression.[52] Moreover, hypoxia-induced VEGF signaling, which is upregulated after cTACE, may additionally impair T cell functionality and modulate increased checkpoint expression.[53,54]

Patients with higher baseline myeloid and lower lymphoid cell counts exhibited poorer responses to LRT, aligning with prior studies linking elevated monocytes and reduced lymphocytes to worse outcomes in HCC after iBT.[55]

Preprocedural monocyte levels are established predictors of poor prognosis following HCC resection and ablation owing to their role in tumor-associated macrophage differentiation, which suppresses anti-tumor immunity.[56]

In addition, lower baseline CD4$^+$ and CD8$^+$ T cell counts have been associated with worse tumor response and survival. Specifically, previous studies showed that high neutrophil- and platelet-to-leucocyte ratios at baseline and increased CD4$^+$ CD25$^+$ Treg counts after treatment were associated with poorer response to TACE.[57–59]

Interestingly, cluster analysis identified a subset of patients with an increasing myeloid response who demonstrated better tumor outcomes according to LI-RADS TRA v2017, contradicting the general assumption that higher myeloid levels predict poor prognosis. This finding underscores the importance of dynamic immune profiling over static baseline assessments in predicting treatment response.

As potential biases resulting from consecutive recruitment and heterogeneity of this cohort cannot be entirely excluded, linear (Supplementary material) and logistic regression (not included in this paper) were conducted to assess the influence of disease characteristics on immune profiles and dynamics. However, no strong correlations were identified between peripheral immune cell profiles and patient demographics, disease characteristics, or laboratory values, potentially because of high statistical uncertainty caused by an insufficient number of events. Notably, previous studies suggest that immune exhaustion differs between viral and non-viral cirrhosis.[59] In this study, however, viral cirrhosis was associated with only a minor, statistically insignificant increase in granulocytes and monocytes compared with non-viral liver disease.

This study has several limitations. It was conducted as a single-center, real-world cohort, resulting in certain heterogeneities in tumor burden, liver function, and cirrhosis etiology. Treatment allocation followed multidisciplinary tumor board recommendations rather than randomization, introducing potential biases. Regression analyses, however, did not reveal strong correlations between immune profiles and patient or disease characteristics. Despite comprehensive flow cytometry and cytokine analyses, some immune cell subsets (*e.g.* Tregs, myeloid-derived suppressor cells, and circulating tumor cells) were not measured because of technical constraints. However, these immune cells and circulating tumor cells are rarely detectable in human peripheral blood samples and may require separate FACS protocols for staining intracellular antigens (*e.g.* FoxP3).[60,61] Blood sampling was limited to baseline, 1 day after LRT, and 2 months after LRT, potentially missing delayed immune effects. Although incorporating thermal ablation techniques could have provided additional insights, particularly in the context of adjuvant trial discussions, ablation therapy was restricted to iBT, as it represents the institution's standard-of-care ablation modality. Furthermore, although tissue biopsies were obtained, local immune responses were not analyzed in this study.

In conclusion, this study demonstrates that LRT induce systemic immune modulation in HCC, with early but transient inflammatory responses that vary by treatment type. The pronounced upregulation of CTLA-4-expressing T cells after cTACE suggests potential synergy with ICI therapy as explored in ongoing trials. Furthermore, despite the complexity of the findings, distinct immune response patterns correlated with treatment outcomes, highlighting the potential of immune profiling to guide personalized therapeutic strategies. Future research should focus on optimizing the timing of ICI administration and validating these findings in larger, longitudinal, multi-center cohorts.

Affiliations

[1]*Charité–Universitätsmedizin Berlin, Campus Virchow-Klinikum, Department of Radiology, Berlin, Germany;* [2]*Experimental Clinical Research Center (ECRC) at Charité–Universitätsmedizin Berlin and Max-Delbrück-Centrum für molekulare Medizin (MDC), Berlin, Germany;* [3]*Charité–Universitätsmedizin Berlin, Institute for*

Biometry and Clinical Epidemiology, Berlin, Germany; [4]Universitätsklinikum Düsseldorf, Department for Gastroenterology, Hepatology and Infectiology, Düsseldorf, Germany; [5]Universitätsklinikum Göttingen, Department of Diagnostic and Interventional Radiology, Göttingen, Germany; [6]Berlin Institute of Health at Charité–Universitätsmedizin Berlin, Berlin, Germany; [7]Charité–Universitätsmedizin Berlin, Campus Virchow-Klinikum (CVK) and Campus Charité Mitte (CCM), Department of Hepatology and Gastroenterology, Berlin, Germany

Abbreviations

BCLC, Barcelona Clinic Liver Cancer; cDC, classical dendritic cell; cTACE, conventional transarterial chemoembolization; DSM, degradable starch microspheres; HCC, hepatocellular carcinoma; iBT, interstitial brachytherapy; ICI, immune checkpoint inhibitor; LI-RADS TRA, Liver Imaging and Data Reporting System Treatment Response Algorithm; LRT; locoregional therapies; MSD, Meso Scale Discovery; (m)RECIST, modified Response Evaluation Criteria in Solid Tumors; RECIST, Response Evaluation Criteria In Solid Tumors; TACE, transarterial chemoembolization; Treg, regulatory T cell.

Financial support

Financial support for this investigator-initiated study was provided by Guerbet, Villepinte, France.

Conflicts of interest

The authors have no conflicts of interest to declare. Outside the submitted work, RS received funding from Berliner Krebsgesellschaft. BG received payment for lectures in the past from Parexel/Calyx, C.R. Bard/BD, Sirtex Medical, St. Jude Medical, Cook, AngioDynamics, Pharmcept, Guerbet, Ewimed, Boston Scientific, Terumo, Roche, Merck, 3M, Beacon Bioscience/ICON, Ipsen, Bayer, Pfizer, Eisai, MSD, Inari, and Siemens/Varian. CAH received funding from Siemens Healthineers and previously received seed funding from the University Medicine Greifswald. FT receives funding from the German Research Foundation (DFG Ta434/8-1, SFB/TRR 296 and CRC1382, Project ID 403224013). LH receives funding from the German Research Foundation (DFG) SPP 2306 and the Else-Kröner-Fresenius-Stiftung. LJS receives funding from the German Research Foundation (Deutsche Forschungsgemeinschaft, DFG): CRC 1340 'Matrix in Vision' (Project ID 372486779) and DFG research unit FOR5628 'Onco-MRE'. LJS receives research grants from Berliner Krebsgesellschaft e.V., Charité 3R, and research grants and honoraria from Guerbet. LJS, CAH, and TAA are fellows of the BIH (Digital) Clinician Scientist Program funded by the Charité–Universitätsmedizin Berlin and the Berlin Institute of Health. The remaining authors declare no conflicts of interest with respect to this manuscript.

Please refer to the accompanying ICMJE disclosure forms for further details.

Authors' contributions

Conceptualization: RS, BG, CR, FT, LH, LJS. Data curation: RS, NA, LH, LJS. Formal analysis: RS, NA, LJS. Funding acquisition: BG, LH, LJS. Investigation: RS, GFT, UF, TAA, GLAP, WL, FP, EC, CAH, LH, LJS. Methodology: RS, LH, LJS. Project administration: BG, CR, RM, FT, BH, LH, LJS. Resources: BG, CR, RM, FT, BH, LH, LJS. Software: RS, NA, LH, LJS. Supervision: BG, CR, RM, FT, BH, LH, LJS. Validation: RS, LH, LJS. Visualization: RS, NA, CR, FT, LH, LJS. Writing—original draft: RS, NA, LH, LJS. Writing—review and editing: RS, BG, NA, CR, GFT, UF, TAA, RM, GLAP, WL, FP, EC, CAH, FT, BH, LH, LJS.

Data availability

All data generated or analyzed during this study are included in this article and its Supplementary material. Further inquiries can be directed to the corresponding author.

Acknowledgements

Parts of this work have been presented at the SIO Annual Meeting 2022, ECR Overture 2022, CIRSE 2022, and RSNA 2021 and 2022. The authors would like to thank the following individuals for their support with this work: Yubei He, Han Xu, Emine Yaren Yilmaz, Yu Liu, Jonas Alexander Leppig, Ines Eichhorn, Dario Zocholl, Dominik Müller, Tobias Penzkofer, Christian Reul, Veronica Prieto, Andreas Wilhelm, and Gerald Grütz.

Statement of ethics

Study approval statement

This prospective, single-center, investigator-initiated observational clinical trial (DRKS00026994) was reviewed and approved by the Institutional Review Board of the Charité–University Medicine Berlin (approval number EA2/091/19), and written informed consent was obtained.

Consent to participate statement

The study protocol conforms with the ethical guidelines of the Declaration of Helsinki as reflected in a prior approval by the institution's human research committee. The manuscript was written in acknowledgement of the STROBE reporting guideline.

Supplementary data

Supplementary data to this article can be found online at https://doi.org/10.1016/j.jhepr.2025.101555.

References

Author names in bold designate shared co-first authorship

[1] Sung H, Ferlay J, Siegel RL, et al. Global Cancer Statistics 2020: GLOBOCAN estimates of incidence and mortality worldwide for 36 cancers in 185 countries. CA Cancer J Clin 2021;71:209–249.

[2] Reig M, Forner A, Rimola J, et al. BCLC strategy for prognosis prediction and treatment recommendation: the 2022 update. J Hepatol 2022;76:681–693.

[3] Heinze C, Damm R, Othmer M, et al. Local tumor control of intermediate and advanced stage hepatocellular carcinoma after local ablative treatment with image-guided interstitial high-dose-rate brachytherapy: a subgroup analysis of 286 HCC nodules. Brachytherapy 2023;22:231–241.

[4] Bretschneider T, Mohnike K, Hass P, et al. Efficacy and safety of image-guided interstitial single fraction high-dose-rate brachytherapy in the management of metastatic malignant melanoma. J Contemp Brachytherapy 2015;7:154–160.

[5] Collettini F, Schnapauff D, Poellinger A, et al. Hepatocellular carcinoma: computed-tomography-guided high-dose-rate brachytherapy (CT-HDRBT) ablation of large (5–7 cm) and very large (>7 cm) tumours. Eur Radiol 2012;22:1101–1109.

[6] Schnapauff D, Tegel BR, Powerski MJ, et al. Interstitial brachytherapy in combination with previous transarterial embolization in patients with unresectable hepatocellular carcinoma. Anticancer Res 2019;39:1329–1336.

[7] Xu Z, Xie H, Zhou L, et al. The combination strategy of transarterial chemoembolization and radiofrequency ablation or microwave ablation against hepatocellular carcinoma. Anal Cell Pathol (Amst) 2019;2019:8619096.

[8] Whittaker S, Marais R, Zhu AX. The role of signaling pathways in the development and treatment of hepatocellular carcinoma. Oncogene 2010;29:4989–5005.

[9] Zhang Y, Zhang Z. The history and advances in cancer immunotherapy: understanding the characteristics of tumor-infiltrating immune cells and their therapeutic implications. Cell Mol Immunol 2020;17:807–821.

[10] Finn RS, Qin S, Ikeda M, et al. Atezolizumab plus bevacizumab in unresectable hepatocellular carcinoma. N Engl J Med 2020;382:1894–1905.

[11] Abou-Alfa GK, Lau G, Kudo M, et al. Tremelimumab plus durvalumab in unresectable hepatocellular carcinoma. NEJM Evid 2022;1:EVIDoa2100070.

[12] Fu Y, Liu S, Zeng S, et al. From bench to bed: the tumor immune microenvironment and current immunotherapeutic strategies for hepatocellular carcinoma. J Exp Clin Cancer Res 2019;38:396.

[13] Gok Yavuz B, Hasanov E, Lee SS, et al. Current landscape and future directions of biomarkers for immunotherapy in hepatocellular carcinoma. J Hepatocell Carcinoma 2021;8:1195–1207.

[14] Giraud J, Chalopin D, Blanc JF, et al. Hepatocellular carcinoma immune landscape and the potential of immunotherapies. Front Immunol 2021;12:655697.

[15] Llovet JM, Castet F, Heikenwalder M, et al. Immunotherapies for hepatocellular carcinoma. Nat Rev Clin Oncol 2022;19:151–172.

[16] Yopp A, Kudo M, Chen M, et al. LBA39 Updated efficacy and safety data from IMbrave050: phase III study of adjuvant atezolizumab (atezo) + bevacizumab (bev) vs active surveillance in patients (pts) with resected or ablated high-risk hepatocellular carcinoma (HCC). Ann Oncol 2024;35:S1230.

[17] Lencioni R, Kudo M, Erinjeri J, et al. EMERALD-1: a phase 3, randomized, placebo-controlled study of transarterial chemoembolization combined with

durvalumab with or without bevacizumab in participants with unresectable hepatocellular carcinoma eligible for embolization. J Clin Oncol 2024;42 (3_suppl). LBA432–LBA432.
[18] Hack SP, Spahn J, Chen M, et al. IMbrave 050: a phase III trial of atezolizumab plus bevacizumab in high-risk hepatocellular carcinoma after curative resection or ablation. Future Oncol 2020;16:975–989.
[19] Li F, Guo Z, Lizée G, et al. Clinical prognostic value of CD4+CD25+FOXP3+regulatory T cells in peripheral blood of Barcelona Clinic Liver Cancer (BCLC) stage B hepatocellular carcinoma patients. Clin Chem Lab Med 2014;52:1357–1365.
[20] Mizukoshi E, Nakamoto Y, Arai K, et al. Comparative analysis of various tumor-associated antigen-specific t-cell responses in patients with hepatocellular carcinoma. Hepatology 2011;53:1206–1216.
[21] Keenan BP, Fong L, Kelley RK. Immunotherapy in hepatocellular carcinoma: the complex interface between inflammation, fibrosis, and the immune response. J Immunother Cancer 2019;7:267.
[22] Lee S, Loecher M, Iyer R. Immunomodulation in hepatocellular cancer. J Gastrointest Oncol 2018;9:208–219.
[23] Li G, Staveley-O'Carroll KF, Kimchi ET. Potential of radiofrequency ablation in combination with immunotherapy in the treatment of hepatocellular carcinoma. J Clin Trials 2016;6:257.
[24] Galluzzi L, Vitale I, Aaronson SA, et al. Molecular mechanisms of cell death: recommendations of the nomenclature committee on cell death 2018. Cell Death Differ 2018;25:486–541.
[25] Moyano DF, Goldsmith M, Solfiell DJ, et al. Nanoparticle hydrophobicity dictates immune response. J Am Chem Soc 2012;134:3965–3967.
[26] de Baere T, Dufaux J, Roche A, et al. Circulatory alterations induced by intra-arterial injection of iodized oil and emulsions of iodized oil and doxorubicin: experimental study. Radiology 1995;194:165–170.
[27] Idée JM, Guiu B. Use of Lipiodol as a drug-delivery system for transcatheter arterial chemoembolization of hepatocellular carcinoma: a review. Crit Rev Oncol Hematol 2013;88:530–549.
[28] Llovet JM, De Baere T, Kulik L, et al. Locoregional therapies in the era of molecular and immune treatments for hepatocellular carcinoma. Nat Rev Gastroenterol Hepatol 2021;18:293–313.
[29] Erinjeri JP, Fine GC, Adema GJ, et al. Immunotherapy and the interventional oncologist: challenges and opportunities—a Society of Interventional Oncology white paper. Radiology 2019;292:25–34.
[30] Cossarizza A, Chang HD, Radbruch A A, et al. Guidelines for the use of flow cytometry and cell sorting in immunological studies (third edition). Eur J Immunol 2021;51:2708–3145.
[31] Schwartz LH, Litière S, de Vries E, et al. Recist 1.1—update and clarification: from the RECIST committee. Eur J Cancer 2016;62:132–137.
[32] Lencioni R, Llovet JM. Modified RECIST (mRECIST) assessment for hepatocellular carcinoma. Semin Liver Dis 2010;30:52–60.
[33] Kielar A, Fowler KJ, Lewis S, et al. Locoregional therapies for hepatocellular carcinoma and the new LI-RADS treatment response algorithm. Abdom Radiol (NY) 2018;43:218–230.
[34] Van der Maaten L, Hinton GE. Visualizing high-dimensional data using t-SNE. J Mach Learn Res 2008;9:2579–2605.
[35] Han JW, Yoon SK. Immune responses following locoregional treatment for hepatocellular carcinoma: possible roles of adjuvant immunotherapy. Pharmaceutics 2021;13:1387.
[36] Pinato DJ, Murray SM, Forner A, et al. Trans-arterial chemoembolization as a loco-regional inducer of immunogenic cell death in hepatocellular carcinoma: implications for immunotherapy. J Immunother Cancer 2021;9:e003311.
[37] Erinjeri JP, Thomas CT, Samoilia A, et al. Image-guided thermal ablation of tumors increases the plasma level of interleukin-6 and interleukin-10. J Vasc Interv Radiol 2013;24:1105–1112.
[38] Bates DO. Vascular endothelial growth factors and vascular permeability. Cardiovasc Res 2010;87:262–271.
[39] Deloch L, Derer A, Hartmann J, et al. Modern radiotherapy concepts and the impact of radiation on immune activation. Front Oncol 2016;6:141.
[40] Galluzzi L, Buqué A, Kepp O, et al. Immunological effects of conventional chemotherapy and targeted anticancer agents. Cancer Cell 2015;28:690–714.
[41] Kimm MA, Kästle S, Stechele MMR, et al. Early monocyte response following local ablation in hepatocellular carcinoma. Front Oncol 2022;12:959987.
[42] Greten TF, Mauda-Havakuk M, Heinrich B, et al. Combined locoregional-immunotherapy for liver cancer. J Hepatol 2019;70:999–1007.
[43] Fife BT, Bluestone JA. Control of peripheral T-cell tolerance and autoimmunity via the CTLA-4 and PD-1 pathways. Immunol Rev 2008;224:166–182.
[44] Berz AM, Santana JG, Iseke S, et al. Impact of chemoembolic regimen on immune cell recruitment and immune checkpoint marker expression following transcatheter arterial chemoembolization in a VX2 rabbit liver tumor model. J Vasc Interv Radiol 2022;33:764–774.e4.
[45] Doemel LA, Santana JG, Savic LJ, et al. Comparison of metabolic and immunologic responses to transarterial chemoembolization with different chemoembolic regimens in a rabbit VX2 liver tumor model. Eur Radiol 2022;32:2437–2447.
[46] Cho Y, Park S, Byun HK, et al. Impact of treatment-related lymphopenia on immunotherapy for advanced non-small cell lung cancer. Int J Radiat Oncol Biol Phys 2019;105:1065–1073.
[47] Tang C, Welsh JW, de Groot P, et al. Ipilimumab with stereotactic ablative radiation therapy: phase I results and immunologic correlates from peripheral T cells. Clin Cancer Res 2017;23:1388–1396.
[48] Crocenzi T, Cottam B, Newell P, et al. A hypofractionated radiation regimen avoids the lymphopenia associated with neoadjuvant chemoradiation therapy of borderline resectable and locally advanced pancreatic adenocarcinoma. J Immunother Cancer 2016;4:45.
[49] Guo J, Wang S, Han Y, et al. Effects of transarterial chemoembolization on the immunological function of patients with hepatocellular carcinoma. Oncol Lett 2021;22:554.
[50] Liao J, Xiao J, Zhou Y, et al. Effect of transcatheter arterial chemoembolization on cellular immune function and regulatory T cells in patients with hepatocellular carcinoma. Mol Med Rep 2015;12:6065–6071.
[51] Park H, Jung JH, Jung MK, et al. Effects of transarterial chemoembolization on regulatory T cell and its subpopulations in patients with hepatocellular carcinoma. Hepatol Int 2020;14:249–258.
[52] Kalathil SG, Lugade AA, Miller A, et al. PD-1^{+} and $Foxp3^{+}$ T cell reduction correlates with survival of HCC patients after sorafenib therapy. JCI Insight 2016;1:e86182.
[53] Petrillo M, Patella F, Pesapane F, et al. Hypoxia and tumor angiogenesis in the era of hepatocellular carcinoma transarterial loco-regional treatments. Future Oncol 2018;14:2957–2967.
[54] Cheng X, Li J, Feng L, et al. The role of hypoxia-related genes in TACE-refractory hepatocellular carcinoma: exploration of prognosis, immunological characteristics and drug resistance based on onco-multi-OMICS approach. Front Pharmacol 2022;13:1011033.
[55] **Kästle S**, **Stechele MR**, **Richter L**, et al. Peripheral blood-based cell signature indicates response to interstitial brachytherapy in primary liver cancer. J Cancer Res Clin Oncol 2023;149:9777–9786.
[56] Shen SL, Fu SJ, Huang XQ, et al. Elevated preoperative peripheral blood monocyte count predicts poor prognosis for hepatocellular carcinoma after curative resection. BMC Cancer 2014;14:744.
[57] Fu J, Zhang Z, Zhou L, et al. Impairment of $CD4^{+}$ cytotoxic T cells predicts poor survival and high recurrence rates in patients with hepatocellular carcinoma. Hepatology 2013;58:139–149.
[58] Xiong B, Feng G, Luo S, et al. Changes of $CD4^{+}$ $CD25^{+}$ regulatory T cells in peripheral blood in patients with hepatocellular carcinoma before and after TACE. J Huazhong Univ Sci Technolog Med Sci 2008;28:645–648.
[59] Schobert IT, Savic LJ, Chapiro J, et al. Neutrophil-to-lymphocyte and platelet-to-lymphocyte ratios as predictors of tumor response in hepatocellular carcinoma after DEB-TACE. Eur Radiol 2020;30:5663–5673.
[60] Bruger AM, Vanhaver C, Bruderek K, et al. Protocol to assess the suppression of T-cell proliferation by human MDSC. Methods Enzymol 2020;632:155–192.
[61] Nikanjam M, Kato S, Kurzrock R. Liquid biopsy: current technology and clinical applications. J Hematol Oncol 2022;15:131.

Keywords: Locoregional therapies; Transarterial chemoembolization; Flow cytometry; Immune phenotyping; Immune response.
Received 24 October 2024; received in revised form 8 August 2025; accepted 12 August 2025; Available online 22 August 2025

Journal of Hepatology, Volume *7*

Supplemental information

Immunologic effects of locoregional therapies for unresectable hepatocellular carcinoma

Robin Schmidt, Bernhard Gebauer, Nilufar Akbari, Christoph Roderburg, Giovanni Federico Torsello, Uli Fehrenbach, Timo Alexander Auer, Raphael Mohr, Gracia Lana Ardila Pardo, Winna Lim, Fabio Pivetta, Elif Can, Charlie Alexander Hamm, Frank Tacke, Bernd Hamm, Linda Hammerich, and Lynn Jeanette Savic

Immunologic effects of locoregional therapies for unresectable hepatocellular carcinoma

Robin Schmidt, Bernhard Gebauer, Nilufar Akbari, Christoph Roderburg, Giovanni Federico Torsello, Uli Fehrenbach, Timo Alexander Auer, Raphael Mohr, Gracia Lana Ardila Pardo, Winna Lim, Fabio Pivetta, Elif Can, Charlie Alexander Hamm, Frank Tacke, Bernd Hamm, Linda Hammerich, Lynn Jeanette Savic

Table of contents

Supplementary methods……………………………………………………………….......2

Supplementary tables…………………………………………………….……….…..6

Supplementary figures………………………………………………………..………25

Supplementary figures legends……………………………………………….…….30

Supplementary references……………………………………………………..…….33

Supplementary methods

Immunostaining and fluorescence-activated cell sorting (FACS)

Blood was collected in ethylenediaminetetraacetic acid-containing tubes, immediately stored at 4° Celsius, and processed for further analysis within a maximum of six hours. A total of 100 µl of whole blood were used for flow cytometry; the remaining sample volume was centrifuged at 3000 rpm for 10 min and the supernatant plasma was archived for further analyses. Viability stain (Fixable viability stain 780, BD Biosciences, Franklin Lake, NJ, USA) was added directly to the whole blood at a dilution of 1:1000 followed by a mix of fluorochrome-conjugated antibodies in FACS blocking buffer (made in house). The staining master mix included monoclonal antibodies against CD1c (L161), CD3 (OKT3), CD11b (M1/70), CD11c (Bu15), CD14 (M5E2), CD16 (3G8), CD19 (HIB19), CD38 (HIT2), CD56 (5.1H11), CD66b (G10F5), CD141 (M80), CTLA-4 (L3D10), HLA-DR (L243), PD1 (NAT105), PD-L1 (29E.2A3), TIM3 (F38-2E2), XCR1 (S15046E, all BioLegend, San Diego, CA, USA), CD4 (RPA-T4) and CD8 (RPA-T8, both BD BioSciences, Franklin Lake, NJ, USA), CD45 (HI30, Thermo Fisher, Waltham, MA, USA), and LAG3 (3DS223H, Thermo Fisher, Waltham, MA, USA). Fluorochrome-Minus-One controls were included for checkpoints, activation, and exhaustion markers. Samples were incubated for 20 minutes at room temperature followed by red blood cell lysis using lysis buffer (1X BD Pharm Lyse, BD Biosciences, Franklin Lake, NJ, USA) for 10 min and centrifugation (500 g, 5 min, 4° Celsius). The supernatant was discarded, and lysis was performed a second time. Samples were fixed with 2% formalin for 10 min at room temperature, washed with PBS and resuspended in 200 µl of PBS for analysis. Additionally, 10 µl of Precision Count Beads^{TM} (BioLegend, San Diego, CA, USA) were added directly before measurement.

Immune cell gating strategy

Samples were analyzed using a Cytek® Aurora flow cytometer (Cytek Biosciences, Fremont, CA, USA). Unmixing and spill-over-correction was performed in SpectroFlo software (Cytek Biosciences, Fremont, CA, USA). Data was analyzed using FCS Express V7 (De Novo Software, Pasadena, CA, USA) with regards to $CD45^+$ lymphoid and myeloid immune cell populations. [1] Gating strategy is depicted in the Supplement Figure 1. Cells were grouped into three morphological subset profiles of 1) T cell response , 2) recruiting and antigen-presenting, and 3) inflammation and phagocytosis, consisting of the following phenotypes: 1) T cells ($CD3^+CD56^-/CD19^-$), and NK-T cells ($CD56^+$ T cells), 2) B cells ($CD19^+/CD3^-/CD56^-$), monocytes ($CD14^+/CD16^+/CD66b^-$), and conventional dendritic cells ($CD11c^+/HLA\text{-}DR^+/CD14^-/CD16^-$), and 3) NK cells ($CD56^+/CD3^-/CD19^-$), neutrophils ($CD66^+/CD11b^+/CD16b^+/CD14^-$) and eosinophils ($CD66^+/CD11b^+/CD16^{low}/CD14^-$). Further markers were used for phenotyping subpopulations such as T-helper cells ($CD4^+$ T cells) and cytotoxic T cells ($CD8^+$ T cells), $CD56^{bright}$ NK cells ($CD56^{high}/CD16^{low}$) and $CD56^{diminished}$ NK cells ($CD56^{low}/CD16^+$), or classical monocytes ($CD14^{high}/CD16^+$) and non-classical monocytes ($CD14^{low}/CD16^+$), as they are described in literature elsewhere. [2-4] In addition, checkpoints, and exhaustion marker profiles e.g., CTLA-4, PD1, PDL-1, TIM3, LAG3 and HLA-DR, CD38, CD161 were assessed on these subpopulations (Supplement Figure 2). Immune cell counts were quantified according to the gating strategy and reported for each time point in absolute and relative numbers. Relative numbers were calculated for each immune subpopulation and the proportion was reported as percentage of all cells in the mother gate (e.g. proportion of $CD4^+$ T cells of all T cells).

Locoregional therapies (LRT)

Conventional transarterial chemoembolization: Selective cTACE was performed using 5 cc of chemotherapy (50 mg doxorubicin in 2.5 cc and 10 mg of mitomycin-C in 2.5 cc) mixed 1:2 with Lipiodol® (Guerbet, Villepinte, France) in a homogenous emulsion [5]. The catheter was inserted through the femoral artery and placed in the tumor feeding artery branches to achieve selective embolization. The emulsion was administered under fluoroscopic guidance into the tumor-feeding arteries, followed by injection of sterile compressed sponge (Gelfoam, Pfizer, New York, NY, USA) until blood flow stasis was reached. In the case of a cTACE/iBT combination therapy, cTACE was performed 24 hours prior to iBT. During iBT, Lipiodol® deposition was visually assessed and aided the catheter placement.

CT-guided interstitial high dose-rate brachytherapy: iBT was performed as described elsewhere [6, 7]. Briefly, iBT was performed under conscious sedation with midazolam and fentanyl, and local anesthesia with lidocaine. Under CT-fluoroscopy, a 6F angiographic sheath was inserted into the lesion, and a closed-end 6F brachytherapy catheter was put through the sheath. For 3D radiation planning, a contrast-enhanced CT scan including at least an arterial and venous phase (15 s and 50 s after injection, respectively) was acquired to depict the catheter in relation to the tumor (slice thickness of 1 mm and 5 mm). The clinical target lesion volume was manually segmented. Radiation was performed using an iridium-192 source enclosing a target dose of 20 Gy for each lesion. Adjacent structures at risk, such as stomach or duodenum, were manually marked and dosages were calculated. Where necessary, overall dosage was modified as described elsewhere [8].

Image acquisition:

MRI scans were obtained on 1,5-T (Avanto, Aera), or 3-T scanners (Skyra; all from Siemens, Munich, Germany) using an eight-channel body phased-array coil. The standard imaging protocol included breath-hold un- and contrast-enhanced T1-weighted sequences (VIBE, in-phase/opposed-phase FLASH) with a hepatocyte-specific contrast agent being applied (0,1 ml/kg intravenous Primovist; Bayer, Germany), T2-weighted sequences (HASTE, TSE), and diffusion-weighted imaging (DWI) with apparent diffusion coefficient (ADC) maps. Dynamic contrast-enhanced images included arterial, portalvenous, venous and hepatobiliary phase (15, 50, 90 seconds and 20 minute after contrast administration, respectively) that were obtained in the axial plane covering the entire liver with 60-72 slices and an adjusted field of view of 255 – 300 mm x 340 – 400 mm (TR 4.26 ms, TE 1.87 ms, flip angle 10°, slice thickness 3 mm, matrix size 256 x 127).

CT scans after cTACE were obtained without contrast agent. Axial slices of 5 mm slice thickness were obtained with field of view of 39,5 mm using a standard kernel within a 512 x 512 matrix, followed by the reconstruction of 0.625-1.0 mm axial slices with multiplanar reconstructions.

Supplementary tables

Table S1: Absolute immune cell counts. Cell concentrations are depicted as n/µl.

	cTACE			iBT			cTACE/iBT		
Time Points	Baseline	One day post	Two months post	Baseline	One day post	Two months post	Baseline	One day post	Two months post
n	27	26	17	68	68	52	43	43	25
**Leucocytes**	**2095.9** [1345.7, 245.4]	**3938.8** [2568.9, 460.5]	**3334.3** [1767.3,3683.8]	**2902.4** [1917.8, 4161.5]	**2826.4** [2241.9, 4668.8]	**2774.5** [1872.4, 4171.5]	**2743.2** [1772.6, 4339.2]	**3169.4** [2354.8, 5333.3]	**2265.4** [1395.7, 3218.2]
**p-values**		**p<0.01**	**p<0.01**		**p=0.04**	p=0.09		p=0.69	**p=0.03**
**Lymphocytes**	**553.1** [410.3, 1177.9]	**418.2** [275.5, 685.6]	**917.4** [487.9, 1310.8]	**765.4** [549.3, 1200.4]	**629.9** [325.5, 843.0]	**805.5** [511.5, 1172.5]	**791.0** [593.9, 1014.0]	**533.6** [361.1, 759.7]	**754.3** [488.8, 898.2]
**p-values**		**p<0.01**	**p<0.01**		**p<0.01**	**p<0.01**		**p=0.01**	p=0.65
**Myeloid Cells**	**1530.9** [795.9, 2347.3]	**3369.4** [2232.9, 5909.8]	**2023.1** [921.4, 2677.8]	**2223.5** [1370.3, 3145.7]	**2188.4** [1789.4 3887.3]	**1949.5** [1174.4, 2885.7]	**2120.8** [1174.1, 3404.5]	**2506.6** [1847.3, 4380	**1590.2** [1040.6, 2685.9]

								.0]	
p-values	**p<0.01**	**p<0.01**		**p<0.01**	**p=0.01**		p=0.33	**p=0.01**	
Classical T cells	**370.7** [220.8, 819.2]	**216.7** [136.9, 400.2]	**621.2** [264.7, 955.3]	**478.9** [333.9, 768.5]	**369.2** [168.9, 504.6]	**501.6** [282.8, 647.6]	**475.6** [343.9, 689.7]	**328.1** [215.4, 498.0]	**391.8** [289.3, 648.8]
p-values	**p<0.01**	**p<0.01**		**p<0.01**	**p<0.01**		**p=0.01**	p=0.52	
$CD4^+$ T cells	**239.4** [169.9, 484.9]	**162.8** [83.1, 226.9]	**513.9** [182.6, 671.1]	**335.3** [192.9, 439.9]	**233.7** [105.2, 304.6]	**322.7** [176.9, 464.8]	**340.2** [256.5, 483.8]	**224.4** [147.4, 298.5]	**301.7** [223.8, 407.8]
p-values	**p<0.01**	**p<0.01**		**p<0.01**	**p<0.01**		**p=0.05**	p=0.96	
$CD8^+$ T cells	**78.8** [45.3, 130.1]	**49.5** [27.9, 106.5]	**76.5** [59.0, 182.9]	**113.4** [47.2, 217.9]	**80.6** [31.4, 155.0]	**101.4** [41.1, 222.8]	**100.6** [44.5, 164.2]	**64.8** [37.2, 113.9]	**67.2** [35.7, 136.2]
p-values	**p<0.01**	**p<0.01**		**p<0.01**	**p<0.01**		**p<0.01**	p=0.34	
$CD4^+$ $CD8^+$ T cells	**1.9** [0.9, 2.9]	**0.9** [0.5, 2.1]	**1.8** [0.9, 3.8]	**2.9** [1.5, 5.8]	**1.9** [0.9, 5.1]	**2.1** [1.2, 5.9]	**2.4** [1.6, 8.2]	**1.8** [0.9, 5.0]	**2.6** [1.0, 7.5]

**p-values**	**p<0.01**	**p<0.01**		**p<0.01**	**p<0.01**		**p=0.03**	p=0.67	
**CD4^{-} CD8^{-} T cells**	**10.8** [8.5, 24.4]	**8.8** [4.0, 19.9]	**13.7** [6.7, 23.4]	**12.7** [6.3, 19.8]	**6.6** [4.0, 17.7]	**11.1** [6.8, 23.0]	**10.9** [6.7, 16.2]	**6.9** [3.9, 11.8]	**8.5** [4.9, 14.7]
**p-values**	**p<0.01**	**p<0.01**		**p<0.01**	**p<0.01**		**p<0.01**	**p<0.01**	
**NK T cells**	**22.9** [9.6, 52.5]	**13.7** [6.6, 35.3]	**26.8** [15.4, 56.9]	**18.6** [7.7, 42.7]	**12.3** [5.1, 35.5]	**13.5** [5.7, 44.5]	**11.1** [4.7, 22.3]	**7.1** [3.9, 17.0]	**9.5** [5.4, 27.5]
**p-values**	**p=0.01**	**p=0.01**		**p=0.03**	**p=0.05**		**p=0.01**	**p=0.04**	
**CD4^{+} NK T cells**	**2.4** [1.2, 5.2]	**1.0** [0.2, 3.3]	**4.3** [2.5, 6.4]	**1.3** [0.7, 2.5]	**0.9** [0,3 2.0]	**1.4** [0.4, 3.6]	**1.6** [0.6, 2.5]	**0.8** [0.4, 2.2]	**1.1** [0.5, 2.56]
**p-values**	**p=0.03**	**p=0.02**		**p=0.01**	**p<0.01**		p=0.39	p=0.81	
**CD8^{+} NK T cells**	**7.4** [3.5, 17.8]	**5.9** [2.0, 12.7]	**7.3** [3.3, 16.8]	**7.2** [3.2, 21.3]	**5.4** [2.2, 14.8]	**5.2** [1.6, 24.9]	**5.6** [2.2, 9.4]	**2.5** [1.6, 6.9]	**4.3** [1.7, 22.1]
**p-values**	**p<0.01**	**p=0.02**		**p=0.03**	p=0.57		**p<0.01**	p=0.16	
**NK cells**	**71.4** [43.5, 141.5]	**75.1** [34.6, 124.2]	**75.5** [51.3, 132.8]	**114.0** [72.4, 184.6]	**84.1** [49.0, 137.4]	**104.2** [71.9, 183.6]	**120.7** [61.5, 207.5]	**49.6** [31.6, 101.0]	**109.1** [57.5, 175.8]
**p-values**	p=0.52	p=0.66		**p<0.01**	**p<0.01**		**p<0.01**	**p<0.01**	

**CD56^{b} ^{right} NK cells**	**6.6** [3.7, 10.8]	**3.2** [1.8, 5.2]	**10.4** [5.0, 20.2]	**7.7** [5.4, 10.8]	**4.7** [3.1, 6.3]	**9.6** [6.7, 13.3]	**9.0** [6.4, 12.3]	**3.2** [1.8, 6.5]	**8.7** [6.0, 14.4]
**p-values**	**p<0.01**		**p<0.01**	**p<0.01**		**p<0.01**	**p<0.01**		**p<0.01**
**CD56^{di} $_{minished}$ NK cells**	**62.2** [39.2, 134.3]	**66.8** [25.9, 118.3]	**60.3** [45.7, 116.2]	**106.6** [64.3, 174.2]	**80.2** [42.2, 129.6]	**92.9** [58.4, 169.4]	**112.5** [55.0, 186.7]	**46.5** [27.4, 91.9]	**103.1** [45.7, 165.3]
**p-values**	p=0.94		p=0.76	**p<0.01**		**p<0.01**	**p<0.01**		**p<0.01**
**B cells**	**51.6** [40.8, 121.1]	**55.4** [33.9, 117.8]	**76.2** [40.2, 101.1]	**76.9** [43.6, 113.0]	**42.7** [23.9, 75.9]	**53.3** [32.8, 96.4]	**99.3** [68.0, 144.5]	**59.2** [27.8, 136.7]	**46.9** [28.4, 83.7]
**p-values**	p=0.41		p=0.09	**p<0.01**		**p=0.01**	p=0.37		**p<0.01**
**Dendritic Cells (cDC)**	**1.5** [0.8, 3.4]	**0.4** [0.2, 1.6]	**1.9** [1.6, 4.0]	**3.3** [1.8, 5.2]	**2.3** [1.1, 4.9]	**2.9** [2.0, 4.3]	**3.5** [2.1, 5.6]	**1.6** [0.9, 2.7]	**2.8** [1.4, 5.2]
**p-values**	**p<0.01**		**p<0.01**	p=0.38		p=0.23	**p=0.01**		**p<0.01**
**Type 1 cDCs**	**0.7** [0.2, 1.1]	**0.2** [0.1, 0.5]	**0.8** [0.5, 1.4]	**1.1** [0.8, 1.9]	**0.8** [0.4, 1.7]	**0.9** [0.6, 1.7]	**0.9** [0.6, 2.3]	**0.5** [0.3, 0.8]	**1.3** [0.4, 1.6]
**p-values**	**p<0.01**		**p<0.01**	p=0.51		p=0.21	**p<0.01**		**p=0.01**

**Type 2 cDCs**	**1.1** [0.4, 2.4]	**0.2** [0.1, 1.2]	**1.3** [0.6, 2.7]	**2.1** [0.9, 3.0]	**1.5** [06, 3.4]	**1.8** [1.2, 2.6]	**1.9** [1.0, 3.4]	**0.9** [0.5, 2.0]	**1.5** [1.0, 2.6]
**p-values**	**p<0.01**		**p<0.01**	p=0.10		p=0.58	**p=0.01**		**p=0.01**
**Monocytes**	**193.5** [87.0, 275.8]	**218.5** [132.3, 407.1]	**261.8** [156.2, 287.6]	**229.7** [149.6, 325.6]	**274.7** [181.9, 377.4]	**222.1** [164.7, 329.4]	**223.1** [124.6, 322.2]	**279.2** [179.1, 396.9]	**222.0** [103.8, 394.4]
**p-values**	**p=0.02**		**p=0.05**	p=0.08		**p=0.03**	p=0.24		**p=0.02**
**Classical Monocytes**	**177.7** [80.5, 250.5]	**209.0** [126.1, 412.4]	**257.1** [143.5, 278.7]	**213.9** [128.4, 307.9]	**257.8** [161.3, 344.0]	**210.4** [141.8, 316.6]	**202.6** [116.5, 278.7]	**272.6** [169.3, 360.9]	**203.7** [91.3, 296.4]
**p-values**	**p=0.01**		**p=0.04**	p=0.07		p=0.06	p=0.19		**p=0.02**
**Non-Classical Monocytes**	**9.6** [3.1, 14.1]	**4.8** [2.7, 7.3]	**9.9** [4.2, 10.7]	**15.3** [6.9, 24.4]	**14.3** [6.6, 23.2]	**11.9** [8.3, 23.2]	**10.9** [7.8, 24.3]	**5.4** [2.7, 13.0]	**13.9** [7.4, 19.1]
**p-values**	**p<0.01**		p=0.06	p=0.82		p=0.98	**p<0.01**		**p=0.04**
**Granu**	**1154.6**	**3183.9**	**1744.8**	**1908.9**	**1822.9**	**1635.6**	**1697.5**	**2256**	**1410.6**

locytes	[586.0, 2103.7]	[2012.1, 5487.0]	[700.8, 2318.7]	[1065.8, 2577.6]	[1436.7, 3429.1]	[966.9, 2544.9]	[929.8, 2969.1]	**.2** [1537.9, 3765.8]	[799.2, 2213.3]
p-values	**p<0.01**		**p<0.01**	**p<0.01**		**p<0.01**	p=0.30		**p=0.01**
Neutrophils	**954.7** [527.2, 1849.6]	**3126.4** [1934.4, 4336.9]	**1646.1** [588.2, 2221.1]	**1677.1** [883.2, 2421.5]	**1739.2** [1322.1, 3181.4]	**1455.2** [859.0, 2491.6]	**1540.6** [629.2, 2703.5]	**1996.8** [1469.1, 3824.7]	**1117.8** [639.8, 1866.1]
p-values	**p<0.01**		**p<0.01**	**p=0.01**		**p=0.03**	p=0.25		**p=0.02**
Eosinophils	**26.1** [18.2, 82.6]	**0.9** [0.2, 2.8]	**37.5** [24.5, 67.6]	**32.8** [17.9, 64.5]	**29.2** [14.9, 53.2]	**45.9** [18.6, 76.8]	**64.3** [21.8, 93.7]	**13.8** [3.1, 49.4]	**35.9** [16.3, 92.3]
p-values	**p<0.01**		**p<0.01**	**p=0.04**		**p<0.01**	**p<0.01**		**p=0.03**
Data is depicted as absolute cell concentrations (n/µl) per time point and respective p-values regarding the changes between 24 hours pre versus 24 hours post, and 24 hours post versus 8 weeks post treatment are depicted in the row below. P-values below p=0.05 are bold printed. cTACE, conventional transarterial chemoembolization. iBT, interstitial brachytherapy. cDCs, conventional dendritic cells.									

Table S2: Alterations of immune cell populations among the time points.

	**From baseline to one day post-LRT**	**From one day post-LRT to two months post-LRT**

Relative Change (%)	Lower CI	Cohens D	Upper CI	p-value	Lower CI	Cohens D	Upper CI	p-value
**Lymphocytes**	-1.22	**-0.99**	-0.78	**p<0.01**	0.68	**0.91**	1.15	**p<0.01**
**Myeloid Cells**	0.73	**0.95**	1.16	**p<0.01**	-1.10	**-0.87**	-0.63	**p<0.01**
**T cells**	-0.28	-0.08	0.13	p=0.14	-0.16	0.07	0.29	p=0.20
**$CD4^+$ T cells**	-0.38	**-0.18**	0.03	**p<0.01**	-0.04	**0.18**	0.40	**p<0.01**
**$CD8^+$ T cells**	-0.06	**0.15**	0.35	**p=0.01**	-0.42	**-0.19**	0.03	**p<0.01**
**$CD4^+CD8^+$ T cells**	-0.28	-0.02	0.19	p=0.46	-0.26	-0.04	0.19	p=0.31
**$CD4^-CD8^-$ T cells**	-0.11	**0.10**	0.31	**p=0.02**	-0.31	-0.08	0.14	p=0.38
**NK T cells**	-0.13	0.08	0.29	p=0.22	-0.28	-0.05	0.17	p=0.58
**$CD4^+$ NK T cells**	-0.24	-0.03	0.18	p=0.98	-0.17	0.06	0.28	p=0.33
**$CD8^+$ NK T cells**	-0.22	-0.01	0.20	p=0.70	-0.34	**-0.12**	0.11	**p<0.01**
**B cells**	-0.04	**0.16**	0.37	**p<0.01**	-0.68	**-0.45**	-0.22	**p<0.01**
**Dendritic Cells (cDC)**	-0.84	**-0.62**	-0.40	**p<0.01**	0.47	**0.71**	0.94	**p<0.01**
**Type 1 cDCs**	-0.11	**0.11**	0.33	p=0.13	-0.28	-0.05	0.19	p=0.36
**Type 2 cDCs**	-0.41	**-0.19**	0.03	**p=0.03**	-0.10	**0.13**	0.36	p=0.11
**Monocytes**	-0.59	**-0.38**	-0.18	**p<0.01**	0.15	**0.37**	0.60	**p<0.01**
**Classical Monocytes**	0.37	**0.58**	0.80	**p<0.01**	-0.79	**-0.56**	-0.32	**p<0.01**
**Non-Classical Monocytes**	-0.83	**-0.62**	-0.40	**p<0.01**	0.39	**0.62**	0.85	**p<0.01**
**NK cells**	-0.13	0.08	0.29	p=0.09	-0.25	-0.03	0.19	p=0.59
**$CD56^{bright}$ NK cells**	-0.60	**-0.39**	-0.18	**p<0.01**	0.32	**0.55**	0.78	**p<0.01**
**$CD56^{diminished}$ NK cells**	0.19	**0.40**	0.61	**p<0.01**	-0.85	**-0.62**	-0.39	**p<0.01**
**Granulocytes**	0.42	**0.63**	0.84	**p<0.01**	-0.82	**-0.59**	-0.36	**p<0.01**

**Neutrophils**	0.52	**0.74**	0.96	**p<0.01**	-1.01	**-0.77**	-0.54	**p<0.01**
**Eosinophils**	-0.57	**-0.36**	-0.15	**p<0.01**	-0.01	**0.22**	0.44	**p=0.01**

**Absolute Change (n/µl)**	Lower CI	Cohens D	Upper CI	p-value	Lower CI	Cohens D	Upper CI	p-value
**Leucocytes**	0.37	**0.59**	0.80	**p<0.01**	-0.79	**-0.56**	-0.33	**p<0.01**
**Lymphocytes**	-0.69	**-0.48**	-0.26	**p<0.01**	0.19	**0.43**	0.66	**p<0.01**
**Myeloid Cells**	0.51	**0.73**	0.95	**p<0.01**	-0.91	**-0.67**	-0.44	**p<0.01**
**T cells**	-0.74	**-0.53**	-0.31	**p<0.01**	0.29	**0.53**	0.76	**p<0.01**
**$CD4^+$ T cells**	-0.77	**-0.56**	-0.34	**p<0.01**	0.29	**0.53**	0.76	**p<0.01**
**$CD8^+$ T cells**	-0.51	**-0.29**	-0.08	**p<0.01**	-0.01	**0.22**	0.45	**p<0.01**
**$CD4^+CD8^+$ T cells**	-0.51	**-0.29**	-0.08	**p<0.01**	-0.04	**0.19**	0.41	**p=0.01**
**$CD4^-CD8^-$ T cells**	-0.49	**-0.28**	-0.07	**p<0.01**	0.05	**0.28**	0.51	**p<0.01**
**NK T cells**	-0.38	**-0.16**	0.06	**p<0.01**	-0.06	**0.17**	0.40	**p=0.01**
**$CD4^+$ NK T cells**	-0.31	-0.09	0.12	p=0.09	-0.06	**0.17**	0.40	**p=0.04**
**$CD8^+$ NK T cells**	-0.32	-0.09	0.12	**p=0.02**	-0.17	0.06	0.29	p=0.09
**B cells**	-0.39	**-0.18**	0.03	p=0.09	-0.32	-0.09	0.14	p=0.14
**Dendritic Cells (cDC)**	-0.70	**-0.45**	-0.25	**p<0.01**	0.17	**0.41**	0.64	**p<0.01**
**Type 1 cDCs**	-0.64	**-0.42**	-0.19	**p<0.01**	0.14	**0.38**	0.61	**p<0.01**
**Type 2 cDCs**	-0.62	**-0.40**	-0.17	**p<0.01**	0.11	**0.34**	0.58	**p<0.01**
**Monocytes**	0.14	**0.36**	0.57	**p<0.01**	-0.57	**-0.34**	-0.11	**p<0.01**
**Classical Monocytes**	0.17	**0.39**	0.61	**p<0.01**	-0.59	**-0.36**	-0.12	**p<0.01**
**Non-Classical Monocytes**	-0.42	**-0.20**	0.02	**p<0.01**	-0.06	**0.18**	0.41	p=0.07

**NK cells**	-0.52	**-0.30**	-0.08	**p<0.01**	0.09	**0.32**	0.55	**p<0.01**
**CD56^{bright} NK cells**	-0.99	**-0.77**	-0.54	**p<0.01**	0.74	**0.98**	1.22	**p<0.01**
**CD56^{diminshed} NK cells**	-0.47	**-0.26**	-0.04	**p<0.01**	0.04	**0.27**	0.50	**p<0.01**
**Granulocytes**	0.53	**0.75**	0.97	**p<0.01**	-0.98	**-0.74**	-0.49	**p<0.01**
**Neutrophils**	0.54	**0.77**	0.99	**p<0.01**	-0.98	**-0.74**	-0.49	**p<0.01**
**Eosinophils**	-0.45	**-0.23**	-0.01	p=0.11	0.05	**0.28**	0.52	**p=0.05**

Data is depicted as Cohens D and lower/upper confidence interval (CI). A Cohens D above/below ± 0.1 is bold printed. cDCs, conventional dendritic cells.

Table S3: Alterations of activation, exhaustion, and checkpoint molecules on all immune cell populations among the time points.

	**From baseline to one day post-LRT**				**From one day to two months post- LRT**			
**Relative Change (%)**	Lower CI	Cohens D	Upper CI	p-value	Lower CI	Cohens D	Upper CI	p-value
**CD4^{+} T cells$_{PD1}$**	-0.20	0.01	0.22	p=0.69	-0.19	0.04	0.26	p=0.49
**CD8^{+} T cells$_{PD1}$**	-0.21	0.01	0.21	p=0.52	-0.28	-0.06	0.17	p=0.90
**CD4^{+} CD8^{+} T cells$_{PD1}$**	-0.20	0.01	0.22	p=0.90	-0.27	-0.04	0.18	p=0.72
**CD4^{-} CD8^{-} T cells$_{PD1}$**	-0.16	0.05	0.25	p=0.64	-0.26	-0.03	0.19	p=0.95
**CD4^{+} NK T cells$_{PD1}$**	-0.18	0.03	0.24	p=0.50	-0.31	-0.08	0.15	p=0.22
**CD8^{+} NK T cells$_{PD1}$**	-0.20	0.01	0.22	p=0.30	-0.26	-0.03	0.20	p=0.49
**CD4^{+} T cells$_{CD38}$**	-0.13	0.08	0.28	**p=0.05**	-0.32	-0.09	0.13	p=0.06
**CD8^{+} T cells$_{CD38}$**	-0.38	**-0.18**	0.03	**p<0.01**	0.02	**0.24**	0.47	**p=0.03**
**CD4^{+} CD8^{+} T cells$_{CD38}$**	-0.09	**0.12**	0.33	p=0.08	-0.14	0.08	0.31	p=0.29
**CD4^{-} CD8^{-} T cells$_{CD38}$**	-0.36	**-0.15**	0.05	**p<0.01**	-0.01	**0.21**	0.44	p=0.13

**CD4$^+$ NK T cells$_{CD38}$**	-0.31	**-0.10**	0.11	p=0.40	-0.23	-0.01	0.22	p=0.86
**CD8$^+$ NK T cells$_{CD38}$**	-0.36	**-0.15**	0.06	**p=0.05**	0.06	**0.29**	0.51	**p=0.01**
**CD4$^+$ T cells$_{PD-L1}$**	-0.17	0.04	0.25	p=0.87	-0.08	**0.14**	0.37	p=0.27
**CD8$^+$ T cells$_{PD-L1}$**	-0.24	-0.03	0.17	p=0.15	-0.26	-0.04	0.19	p=0.41
**CD4$^+$ CD8$^+$ T cells$_{PD-L1}$**	-0.08	**0.13**	0.34	p=0.09	-0.25	-0.03	0.20	p=0.64
**CD4$^-$ CD8$^-$ T cells$_{PD-L1}$**	-0.30	-0.09	0.12	p=0.59	-0.20	0.03	0.25	p=0.77
**CD4$^+$ NK T cells$_{PD-L1}$**	-0.32	**-0.11**	0.10	p=0.44	-0.13	**0.10**	0.32	p=0.83
**CD8$^+$ NK T cells$_{PD-L1}$**	-0.17	0.04	0.24	p=0.34	-0.20	0.02	0.24	p=0.26
**CD4$^+$ T cells$_{CTLA-4}$**	0.33	**0.54**	0.75	**p<0.01**	-0.65	**-0.42**	-0.20	**p<0.01**
**CD8$^+$ T cells$_{CTLA-4}$**	0.36	**0.57**	0.78	**p<0.01**	-0.35	**-0.13**	0.10	p=0.26
**CD4$^+$ CD8$^+$ T cells$_{CTLA-4}$**	-0.10	**0.10**	0.31	p=0.34	-0.59	**-0.36**	-0.14	**p<0.01**
**CD4$^-$ CD8$^-$ T cells$_{CTLA-4}$**	0.14	**0.35**	0.56	**p<0.01**	-0.56	**-0.33**	-0.11	**p<0.01**
**CD4$^+$ NK T cells$_{CTLA-4}$**	-0.15	0.06	0.26	p=0.55	-0.11	**0.11**	0.34	p=0.53
**CD8$^+$ NK T cells$_{CTLA-4}$**	0.02	**0.23**	0.43	p=0.16	-0.20	0.03	0.25	p=0.08
**CD4$^+$ T cells$_{HLA-DR}$**	-0.27	-0.06	0.14	p=0.73	-0.05	**0.18**	0.40	p=0.08
**CD8$^+$ T cells$_{HLA-DR}$**	-0.38	**-0.17**	0.03	p=0.08	0.25	**0.47**	0.70	**p<0.01**
**CD4$^+$ CD8$^+$ T cells$_{HLA-DR}$**	-0.28	-0.07	0.14	p=0.07	-0.10	**0.13**	0.35	**p<0.01**
**CD4$^-$ CD8$^-$ T cells$_{HLA-DR}$**	-0.39	**-0.18**	0.03	**p=0.02**	0.17	**0.39**	0.62	**p<0.01**
**CD4$^+$ NK T cells$_{HLA-DR}$**	-0.20	0.01	0.22	p=0.86	-0.03	**0.19**	0.42	p=0.08
**CD8$^+$ NK T cells$_{HLA-DR}$**	-0.35	**-0.14**	0.07	p=0.12	0.12	**0.35**	0.57	**p=0.01**
**CD4$^+$ T cells$_{CD161}$**	-0.37	**-0.15**	0.07	p=0.07	0.14	**0.38**	0.61	**p<0.01**
**CD8$^+$ T cells$_{CD161}$**	-0.36	**-0.15**	0.07	**p<0.01**	-0.13	**0.11**	0.34	**p<0.01**
**CD4$^+$ CD8$^+$ T cells$_{CD161}$**	-0.21	0.01	0.23	p=0.76	-0.27	-0.04	0.20	p=0.16
**CD4$^-$ CD8$^-$ T cells$_{CD161}$**	-0.32	**-0.10**	0.13	p=0.31	-0.19	0.05	0.29	p=0.20
**CD4$^+$ NK T cells$_{CD161}$**	-0.40	**-0.18**	0.04	p=0.14	-0.13	**0.11**	0.35	p=0.51
**CD8$^+$ NK T cells$_{CD161}$**	0.07	**0.29**	0.50	**p<0.01**	-0.70	**-0.46**	-0.23	**p=0.02**

**CD4⁺ T cells$_{TIM3}$**	0.07	**0.29**	0.50	**p=0.03**	-0.69	**-0.46**	-0.23	**p<0.01**
**CD8⁺ T cells$_{TIM3}$**	-0.17	0.04	0.26	p=0.38	-0.22	0.01	0.25	p=0.81
**CD4⁺ CD8⁺ T cells$_{TIM3}$**	-0.27	-0.06	0.15	p=0.85	0.05	**0.29**	0.52	**p=0.01**
**CD4⁻ CD8⁻ T cells$_{TIM3}$**	-0.22	-0.01	0.21	p=0.87	-0.40	**-0.17**	0.07	**p<0.01**
**CD4⁺ NK T cells$_{TIM3}$**	-0.28	-0.07	0.15	p=0.85	0.02	**0.26**	0.50	p=0.13
**CD8⁺ NK T cells$_{TIM3}$**	-0.35	**-0.14**	0.08	**p=0.04**	-0.11	**0.13**	0.36	**p=0.04**
**CD4⁺ T cells$_{LAG3}$**	-0.18	0.04	0.26	p=0.26	-0.57	**-0.34**	-0.11	**p=0.01**
**CD8⁺ T cells$_{LAG3}$**	-0.18	0.03	0.25	p=0.19	-0.45	**-0.21**	0.02	p=0.08
**CD4⁺ CD8⁺ T cells$_{LAG3}$**	-0.27	-0.06	0.16	p=0.85	0.04	**0.28**	0.51	**p=0.01**
**CD4⁻ CD8⁻ T cells$_{LAG3}$**	-0.21	0.01	0.21	p=0.83	-0.41	**-0.18**	0.06	**p=0.02**
**CD4⁺ NK T cells$_{LAG3}$**	-0.23	-0.01	0.21	p=0.30	-0.49	**-0.27**	-0.03	**p=0.03**
**CD8⁺ NK T cells$_{LAG3}$**	-0.18	0.05	0.27	p=0.24	-0.57	**-0.34**	-0.10	**p=0.01**
**B cells$_{CD11b}$**	-0.09	**0.12**	0.33	p=0.76	-0.19	0.04	0.26	p=0.58
**B cells$_{CD19high}$**	-0.23	**-0.27**	0.18	p=0.93	-0.09	**0.13**	0.36	p=0.13
**B cells$_{CD38high}$**	-0.21	-0.01	0.20	p=0.33	-0.58	**-0.36**	-0.13	**p<0.01**
**B cells$_{CD38int}$**	-0.23	-0.03	0.18	p=0.90	-0.09	**0.13**	0.36	**p<0.01**
**B cells$_{CD38low}$**	-0.13	0.09	0.30	p=0.58	-0.04	**0.19**	0.42	**p=0.02**
**B cells$_{PD-L1}$**	-0.12	0.09	0.30	p=0.67	-0.29	-0.06	0.17	**p=0.04**
**Classical Monocytes$_{CD1c}$**	-0.11	**0.10**	0.32	p=0.10	-0.14	0.09	0.32	p=0.09
**Non-Classical Monocytes$_{PD-L1}$**	-0.44	**-0.22**	-0.01	p=0.64	0.07	**0.30**	0.53	p=0.13
**Classical Monocytes$_{CTLA-4}$**	-0.20	0.01	0.22	p=0.64	-0.39	**-0.16**	0.07	p=0.62
**Non-Classical Monocytes$_{CTLA-4}$**	-0.28	-0.06	0.15	p=0.08	-0.16	0.07	0.30	p=0.18
**Classical**	-0.41	**-0.20**	0.02	**p=0.05**	0.36	**0.59**	0.82	**p<0.01**

**Monocytes**$_{\text{HLA-DR}}$								
**Non-Classical Monocytes**$_{\text{HLA-DR}}$	-0.19	0.02	0.23	p=0.85	0.11	**0.34**	0.57	**p<0.01**
**Classical Monocytes**$_{\text{TIM3}}$	-0.41	**-0.19**	0.03	**p=0.01**	-0.26	-0.02	0.22	p=0.55
**Non-Classical Monocytes**$_{\text{TIM3}}$	-0.60	**-0.38**	-0.16	**p<0.01**	0.01	**0.24**	0.48	**p<0.01**
**Classical Monocytes**$_{\text{CD56}}$	0.19	**0.40**	0.62	**p<0.01**	-0.62	**-0.39**	-0.16	**p<0.01**
**Non-Classical Monocytes**$_{\text{CD56}}$	-0.25	-0.04	0.18	p=0.38	0.04	**0.27**	0.50	p=0.04
**NK cells**$_{\text{PD-1}}$	-0.20	0.01	0.22	p=0.98	-0.14	0.09	0.31	p=0.58
**NK cells**$_{\text{HLA-DR}}$	-0.25	-0.05	0.16	**p=0.05**	0.04	**0.27**	0.50	p=0.46
**NK cells**$_{\text{CD161}}$	-0.28	-0.06	0.16	**p=0.01**	-0.19	0.04	0.28	p=0.07
**NK cells**$_{\text{CD8}}$	-0.23	-0.02	0.19	p=0.73	-0.39	**-0.17**	0.06	**p=0.01**
**Eosinophils**$_{\text{HLA-DR}}$	-0.25	-0.03	0.18	p=0.54	-0.05	**0.18**	0.41	p=0.28
**Absolute Change (%)**	Lower CI	Cohens D	Upper CI	p-value	Lower CI	Cohens D	Upper CI	p-value
**CD4^{+} T cells**$_{\text{PD1}}$	-0.70	**-0.49**	-0.27	**p<0.01**	0.28	**0.51**	0.74	**p<0.01**
**CD8^{+} T cells**$_{\text{PD1}}$	-0.45	**-0.23**	-0.02	**p<0.01**	-0.02	**0.21**	0.44	**p<0.01**
**CD4^{+} CD8^{+} T cells**$_{\text{PD1}}$	-0.46	**-0.24**	-0.03	**p=0.01**	-0.07	**0.16**	0.39	p=0.09
**CD4^{-} CD8^{-} T cells**$_{\text{PD1}}$	-0.45	**-0.24**	-0.02	**p<0.01**	0.08	**0.31**	0.54	**p<0.01**
**CD4^{+} NK T cells**$_{\text{PD1}}$	-0.28	-0.07	0.15	p=0.29	-0.10	**0.16**	0.36	p=0.44
**CD8^{+} NK T cells**$_{\text{PD1}}$	-0.28	-0.06	0.15	**p<0.01**	-0.19	0.04	0.27	**p=0.01**
**CD4^{+} T cells**$_{\text{CD38}}$	-0.59	**-0.37**	-0.15	**p<0.01**	0.10	**0.33**	0.56	**p<0.01**
**CD8^{+} T cells**$_{\text{CD38}}$	-0.54	**-0.32**	-0.10	**p<0.01**	0.09	**0.32**	0.54	**p<0.01**

**$CD4^+$ $CD8^+$ T cells$_{CD38}$**	-0.37	**-0.15**	0.06	p=0.08	-0.07	**0.16**	0.39	p=0.29
**$CD4^-$ $CD8^-$ T cells$_{CD38}$**	-0.48	**-0.26**	-0.05	**p<0.01**	0.04	**0.27**	0.50	**p<0.01**
**$CD4^+$ NK T cells$_{CD38}$**	-0.29	-0.08	0.14	p=0.26	-0.12	**0.11**	0.34	p=0.49
**$CD8^+$ NK T cells$_{CD38}$**	-0.36	**-0.14**	0.07	**p=0.02**	-0.07	**0.16**	0.40	p=0.15
**$CD4^+$ T cells$_{PD-L1}$**	-0.27	-0.05	0.17	**p=0.03**	-0.08	**0.15**	0.34	p=0.25
**$CD8^+$ T cells$_{PD-L1}$**	-0.40	**-0.18**	0.03	p=0.20	-0.06	**0.17**	0.40	p=0.34
**$CD4^+$ $CD8^+$ T cells$_{PD-L1}$**	-0.17	0.04	0.26	p=0.81	-0.13	**0.10**	0.33	p=0.29
**$CD4^-$ $CD8^-$ T cells$_{PD-L1}$**	-0.54	**-0.32**	-0.10	**p=0.05**	-0.02	**0.21**	0.44	p=0.15
**$CD4^+$ NK T cells$_{PD-L1}$**	-0.38	**-0.16**	0.06	**p=0.02**	-0.20	0.03	0.26	p=0.13
**$CD8^+$ NK T cells$_{PD-L1}$**	-0.29	-0.07	0.14	**p=0.04**	-0.25	-0.02	0.21	p=0.20
**$CD4^+$ T cells$_{CTLA-4}$**	0.01	**0.23**	0.44	**p<0.01**	-0.35	**-0.12**	0.11	**p=0.13**
**$CD8^+$ T cells$_{CTLA-4}$**	0.31	**0.53**	0.75	**p<0.01**	-0.62	**-0.39**	-0.16	**p<0.01**
**$CD4^+$ $CD8^+$ T cells$_{CTLA-4}$**	0.06	**0.28**	0.50	**p<0.01**	-0.71	**-0.48**	-0.24	**p<0.01**
**$CD4^-$ $CD8^-$ T cells$_{CTLA-4}$**	0.12	**0.33**	0.55	**p<0.01**	-0.42	**-0.19**	0.04	**p<0.01**
**$CD4^+$ NK T cells$_{CTLA-4}$**	-0.21	0.01	0.22	p=0.08	-0.10	**0.13**	0.36	p=0.27
**$CD8^+$ NK T cells$_{CTLA-4}$**	-0.11	**0.11**	0.32	p=0.28	-0.16	0.07	0.30	p=0.59
**$CD4^+$ T cells$_{HLA-DR}$**	-0.78	**-0.57**	-0.35	**p<0.01**	0.30	**0.53**	0.77	**p<0.01**
**$CD8^+$ T cells$_{HLA-DR}$**	-0.58	**-0.36**	-0.15	**p<0.01**	0.28	**0.52**	0.75	**p<0.01**
**$CD4^+$ $CD8^+$ T cells$_{HLA-DR}$**	-0.45	**-0.24**	-0.02	**p<0.01**	-0.01	**0.23**	0.45	**p<0.01**
**$CD4^-$ $CD8^-$ T cells$_{HLA-DR}$**	-0.52	**-0.30**	-0.09	**p<0.01**	0.22	**0.45**	0.68	**p<0.01**
**$CD4^+$ NK T cells$_{HLA-DR}$**	-0.39	**-0.17**	0.05	**p=0.02**	-0.02	**0.21**	0.44	p=0.08
**$CD8^+$ NK T cells$_{HLA-DR}$**	-0.35	**-0.14**	0.08	**p=0.01**	-0.03	**0.20**	0.43	p=0.26
**$CD4^+$ T cells$_{CD161}$**	-0.72	**-0.50**	-0.28	**p<0.01**	0.34	**0.58**	0.82	**p<0.01**
**$CD8^+$ T cells$_{CD161}$**	-0.49	**-0.27**	-0.05	**p<0.01**	-0.01	**0.23**	0.47	**p<0.01**
**$CD4^+$ $CD8^+$ T cells$_{CD161}$**	-0.46	**-0.24**	-0.02	**p<0.01**	0.01	**0.25**	0.49	**p<0.01**
**$CD4^-$ $CD8^-$ T cells$_{CD161}$**	-0.35	**-0.13**	0.09	**p<0.01**	-0.03	**0.21**	0.45	**p<0.01**

**CD4$^+$ NK T cells$_{CD161}$**	-0.43	**-0.20**	0.02	**p=0.05**	-0.11	**0.13**	0.37	p=0.16
**CD8$^+$ NK T cells$_{CD161}$**	-0.13	0.09	0.31	**p<0.01**	-0.51	**-0.28**	-0.04	p=0.16
**CD4$^+$ T cells$_{TIM3}$**	-0.13	0.09	0.31	p=0.40	-0.51	**-0.28**	-0.04	**p=0.01**
**CD8$^+$ T cells$_{TIM3}$**	-0.29	-0.08	0.13	p=0.23	-0.24	-0.06	0.23	p=0.89
**CD4$^+$ CD8$^+$ T cells$_{TIM3}$**	-0.39	**-0.18**	0.04	p=0.22	0.09	**0.33**	0.57	**p=0.02**
**CD4$^-$ CD8$^-$ T cells$_{TIM3}$**	-0.54	**-0.33**	-0.11	**p<0.01**	-0.10	**0.13**	0.37	**p=0.04**
**CD4$^+$ NK T cells$_{TIM3}$**	-0.16	0.06	0.28	p=0.42	-0.10	**0.13**	0.37	p=0.60
**CD8$^+$ NK T cells$_{TIM3}$**	-0.40	**-0.19**	0.03	**p=0.01**	-0.03	**0.21**	0.45	p=0.08
**CD4$^+$ T cells$_{LAG3}$**	-0.21	0.01	0.23	p=0.59	-0.48	**-0.26**	-0.02	**p=0.03**
**CD8$^+$ T cells$_{LAG3}$**	-0.18	0.04	0.26	p=0.28	-0.47	**-0.24**	-0.07	**p=0.04**
**CD4$^+$ CD8$^+$ T cells$_{LAG3}$**	-0.39	**-0.18**	0.04	**p=0.03**	0.09	**0.33**	0.57	**p<0.01**
**CD4$^-$ CD8$^-$ T cells$_{LAG3}$**	-0.55	**-0.33**	-0.11	**p<0.01**	-0.09	**0.14**	0.37	p=0.07
**CD4$^+$ NK T cells$_{LAG3}$**	-0.16	0.05	0.27	p=0.27	-0.32	-0.09	0.14	p=0.33
**CD8$^+$ NK T cells$_{LAG3}$**	-0.14	0.08	0.29	p=0.41	-0.42	**-0.19**	0.05	p=0.08
**B cells$_{CD11b}$**	-0.26	-0.04	0.17	p=0.19	-0.18	0.05	0.28	p=0.26
**B cells$_{CD19high}$**	-0.46	**-0.25**	-0.04	p=0.42	-0.17	-0.06	0.29	p=0.40
**B cells$_{CD38high}$**	-0.43	**-0.21**	0.01	**p<0.01**	0.01	**0.24**	0.47	p=0.18
**B cells$_{CD38int}$**	-0.41	**-0.20**	0.11	**p=0.05**	-0.39	-0.08	0.15	**p=0.01**
**B cells$_{CD38low}$**	-0.22	0.01	0.22	p=0.18	-0.013	**0.22**	0.45	p=0.16
**B cells$_{PD-L1}$**	-0.42	**-0.21**	0.01	p=0.11	0.01	**0.23**	0.48	p=0.15
**Classical Monocytes$_{CD1c}$**	0.14	**0.36**	0.58	**p<0.01**	-0.46	**-0.23**	0.01	**p=0.01**
**Non-Classical Monocytes$_{CD1c}$**	-0.22	0.01	0.22	**p=0.01**	-0.13	**0.22**	0.45	**p=0.01**
**Non-Classical Monocytes$_{PD-L1}$**	-0.53	**-0.31**	-0.09	**p<0.01**	0.11	**0.35**	0.58	**p<0.01**

**Classical Monocytes**$_{CTLA-4}$	0.08	**0.30**	0.52	p=0.78	-0.52	**-0.28**	-0.05	p=0.68
**Non-Classical Monocytes**$_{CTLA-4}$	-0.31	-0.08	0.14	**p<0.01**	-0.25	0.01	0.22	p=0.20
**Classical Monocytes**$_{HLA-DR}$	0.03	**0.25**	0.47	p=0.74	-0.23	0.01	0.24	p=0.76
**Non-Classical Monocytes**$_{HLA-DR}$	-0.27	-0.05	0.17	**p<0.01**	-0.07	**0.16**	0.39	**p<0.01**
**Classical Monocytes**$_{TIM3}$	-0.03	**0.19**	0.41	p=0.60	-0.50	**-0.26**	-0.03	p=0.37
**Non-Classical Monocytes**$_{TIM3}$	-0.63	**-0.41**	0.19	**p<0.01**	-0.04	**0.28**	0.52	**p<0.01**
**Classical Monocytes**$_{CD56}$	0.22	**0.44**	0.67	**p<0.01**	-0.71	**-0.48**	-0.24	**p<0.01**
**Non-Classical Monocytes**$_{CD56}$	-0.42	**-0.19**	0.03	p=0.15	0.15	**0.38**	0.62	p=0.79
**NK cells**$_{PD-1}$	-0.33	**-0.11**	0.10	p=0.11	0.02	**0.23**	0.46	**p<0.01**
**NK cells**$_{HLA-DR}$	-0.52	**-0.30**	-0.09	p=0.23	0.33	**0.57**	0.79	**p<0.01**
**NK cells**$_{CD161}$	-0.52	**-0.31**	-0.09	**p<0.01**	0.12	**0.36**	0.60	**p<0.01**
**NK cells**$_{CD8}$	-0.50	**-0.28**	-0.07	**p<0.01**	0.01	**0.24**	0.47	**p<0.01**
**Eosinophils**$_{HLA-DR}$	-0.61	**-0.39**	-0.16	**p=0.02**	0.25	**0.48**	0.72	**p=0.04**

Data is depicted as Cohens D and lower/upper confidence interval (CI). A Cohens D above/below ± 0.1 is bold printed. PD1, programmed cell death protein 1 (CD279), PD-L1, programmed cell death protein 1 ligand 1 (CD274). CTLA-4, cytotoxic T-lymphocyte-associated Protein 4 (CD152). HLA-DR, human leucocyte antigen DR. TIM3, T-cell immunoglobulin and mucin-domain containing-3 (CD366). LAG3, lymphocyte-activation gene 3 (CD223).

Table S4. Alterations of cytokines and chemokines from baseline to one day post-cTACE.

Variable	Lower CI	Cohen's D	Upper CI	p-value
IL-1β	-0.24	0.29	0.81	<0.01
IL-2	-0.40	0.12	0.64	0.29
IL-4	-0.62	-0.10	0.42	0.94
IL-5	-0.62	-0.10	0.42	0.34
IL-6	-0.11	0.41	0.94	0.96
IL-8	-1.05	-0.53	0.00	0.94
IL-10	-0.36	0.16	0.68	0.31
IL-13	-0.55	-0.04	0.48	0.03
IL-17	-0.98	-0.46	0.07	0.01
MIP1α	-0.67	-0.16	0.37	0.01
MCP1	-1.07	-0.54	-0.01	<0.01
VEGF	-0.67	-0.15	0.37	<0.01
INFγ	-1.27	-0.73	-0.19	0.03
TNFα	-0.80	-0.28	0.25	0.11
βFGF	-0.45	0.07	0.59	0.73

Table S5. Tumor response after brachytherapy (iBT), conventional transarterial chemoembolization (cTACE), and cTACE/iBT according to response evaluation criteria in solid tumors (RECIST), modified (m)RECIST, and Liver Imaging and Data Reporting System – Treatment Response Algorithm version 2017 (LI-TRA v2017).

	1st Follow-Up (2 months post-LRT)			2nd Follow-Up (6 months post-LRT)			3rd Follow-Up (12 months post-LRT)		
**RECIST**	**cTACE** n = 20	**iBT** n = 58	**cTACE/iBT** n = 37	**cTACE** n = 14	**iBT** n = 55	**cTACE/iBT** n = 30	**cTACE** n = 4	**iBT** n = 47	**cTACE/iBT** n = 32
**CR**	-	-	-	-	-	-	-	-	-
**PR**	30.0 % (6)	20.7 % (12)	29.7 % (11)	42.9 % (6)	32.7 % (18)	53.3 % (16)	50.0 % (2)	46.8 % (22)	75.0 % (24)
**SD**	70.0 % (14)	74.1 % (43)	64.9 % (24)	50.0 % (7)	61.8 % (34)	40.0 % (12)	50.0 % (2)	51.1 % (24)	25.0 % (8)
**PD**	-	5.2 % (3)	5.4 % (2)	7.1 % (1)	5.5 % (3)	6.7 % (2)	-	2.1 % (1)	-
**mRECIST**	**cTACE** n = 20	**iBT** n = 57	**cTACE/iBT** n = 36	**cTACE** n = 14	**iBT** n = 54	**cTACE/iBT** n = 29	**cTACE** n = 4	**iBT** n = 46	**cTACE/iBT** n = 31
**CR**	55.0 % (11)	66.7 % (38)	63.9 % (23)	50.0 % (7)	68.5 % (37)	65.5 % (19)	50.0 % (2)	71.7 % (33)	74.2 % (23)
**PR**	35.0 %	15.8	25.0 % (9)	21.4 %	11.1	23.1 % (7)	-	6.5 %	12.9 % (4)

	(7)	% (9)		(3)	% (6)			(3)	
**SD**	5.0 % (2)	17.5 % (10)	8.3 % (3)	14.3 % (2)	16.7 % (9)	6.9 % (2)	25.0 % (1)	13.0% (6)	9.7 % (3)
**PD**	-	-	2.8 % (1)	14.3 % (2)	3.7 % (2)	3.5% (1)	25.0 % (1)	8.7 % (4)	3.2 % (1)
**LI-TRA v2017**	**cTACE** n = 22	**iBT** n = 62	**cTACE/iBT** n = 39	**cTACE** n = 13	**iBT** n = 56	**cTACE/iBT** n = 32	**cTACE** n = 8	**iBT** n = 58	**cTACE/iBT** n = 28
**Non-Viable**	31.8 % (7)	33.9 % (21)	28.2 % (11)	38.5 % (5)	39.3 % (22)	28.1 % (9)	16.7 % (1)	55.1 % (27)	32.1 % (9)
**Equivocal**	18.2 % (4)	38.7 % (24)	43.6 % (17)	7.7 % (1)	26.8 % (15)	31.2 % (10)	16.7 % (1)	14.3 % (7)	28.6 % (8)
**Viable**	50.0 % (11)	27.4 % (17)	28.2 % (11)	53.8 % (7)	33.9 % (19)	40.6 % (13)	66.7 % (4)	30.6 % (24)	39.3 % (11)

CR complete response; PR partial response; SD stable disease; PD progressive disease; LRT locoregional therapy.

Postinterventional follow-up times: 1st Follow-Up: 2 months, 2nd Follow-Up: 6 months, 3rd Follow-Up: 12 months.

Table S6. Immune alterations from baseline to one day post-locoregional therapy (LRT) as well as treatment response according to LIRADS Treatment Response Algorithm (LI-TRA) v2017 according to immune clustering including $PD1^+$, $PDL\text{-}1^+$ and $CTLA4^+$ $CD4^+$ and $CD8^+$ T cells as revealed by TSNE- analysis.

**Immune cell population**	**Cluster 1 (n=68)**<br>Cohen's D (95% CI)	**Cluster 2 (n=39)**<br>Cohen's D (95% CI)
B cells	-0.45 [-0.61,-0.30]	0.14 [-0.10,0.47]
T cells	-0.43 [-0.56, -0.33]	0.07 [0.10,0.24]
NK cells	-0.37 [-0.62, -0.15]	-0.12 [-0.30, 0.15]
Monocytes	-0.12 [-0.43, 0.40]	1.05 [0.59, 2.67]
Granulocytes	0.24 [-0,34, 0.90]	2.45 [0.81, 4.73]
cDCs	-0,52 [-0.80, -0.10]	0.18 [-0.34, 0.97]
**LI-TRA v2017**<br>6 months post-LRT		
Non-viable	15 (30.6%)	12 (36.4%)
Equivocal	7 (14.3%)	12 (36.4%)
Viable	27 (55.1%)	9 (27.3%)

Supplementary figures

Fig. S1

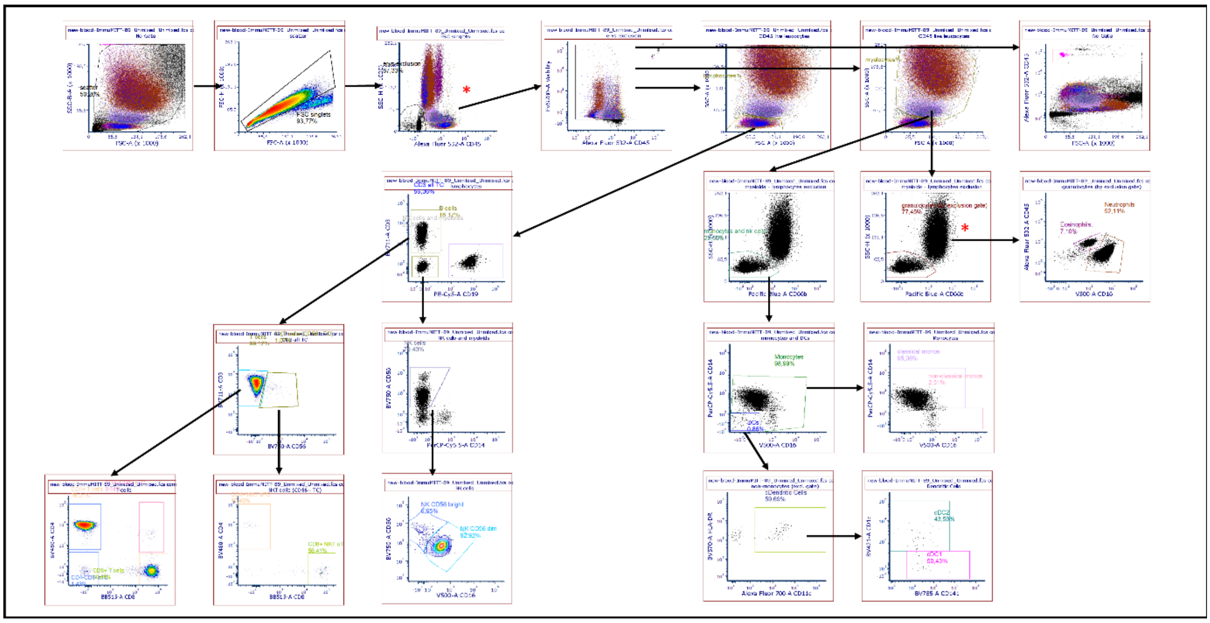

Fig. S2

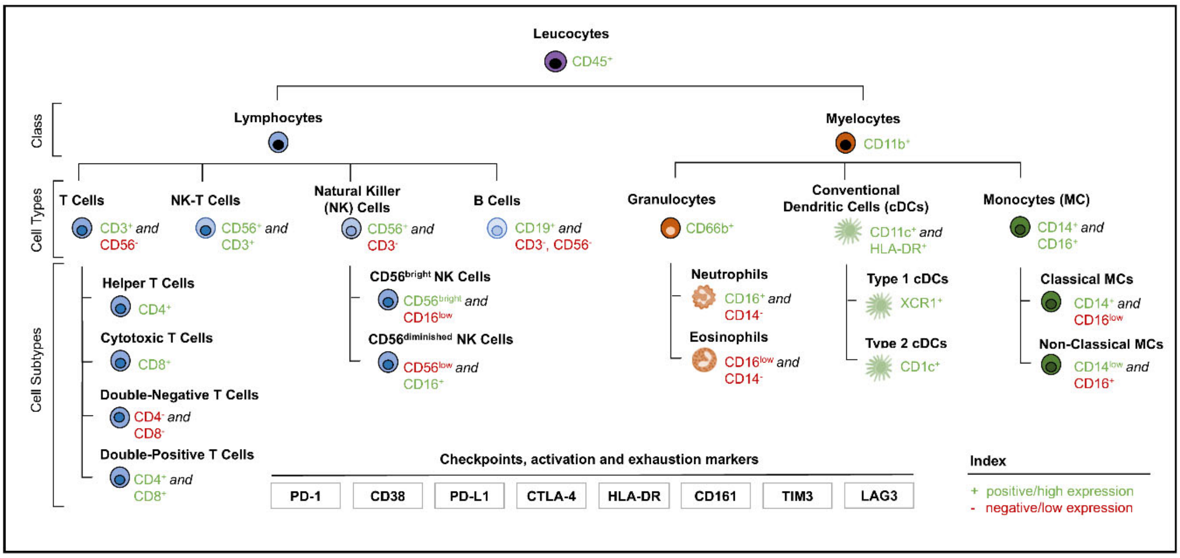

Fig. S3

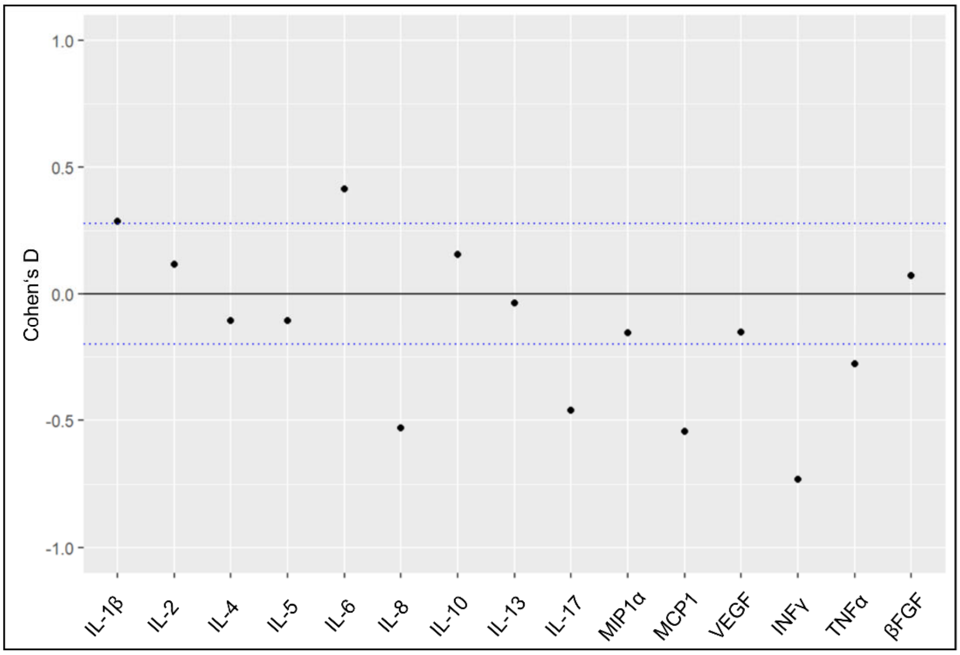

Cohen's D
1.0
0.5
0.0
-0.5
-1.0
IL-1β
IL-2
IL-4
IL-5
IL-6
IL-8
IL-10
IL-13
IL-17
MIP1α
MCP1
VEGF
INFγ
TNFα
βFGF

Fig. S4

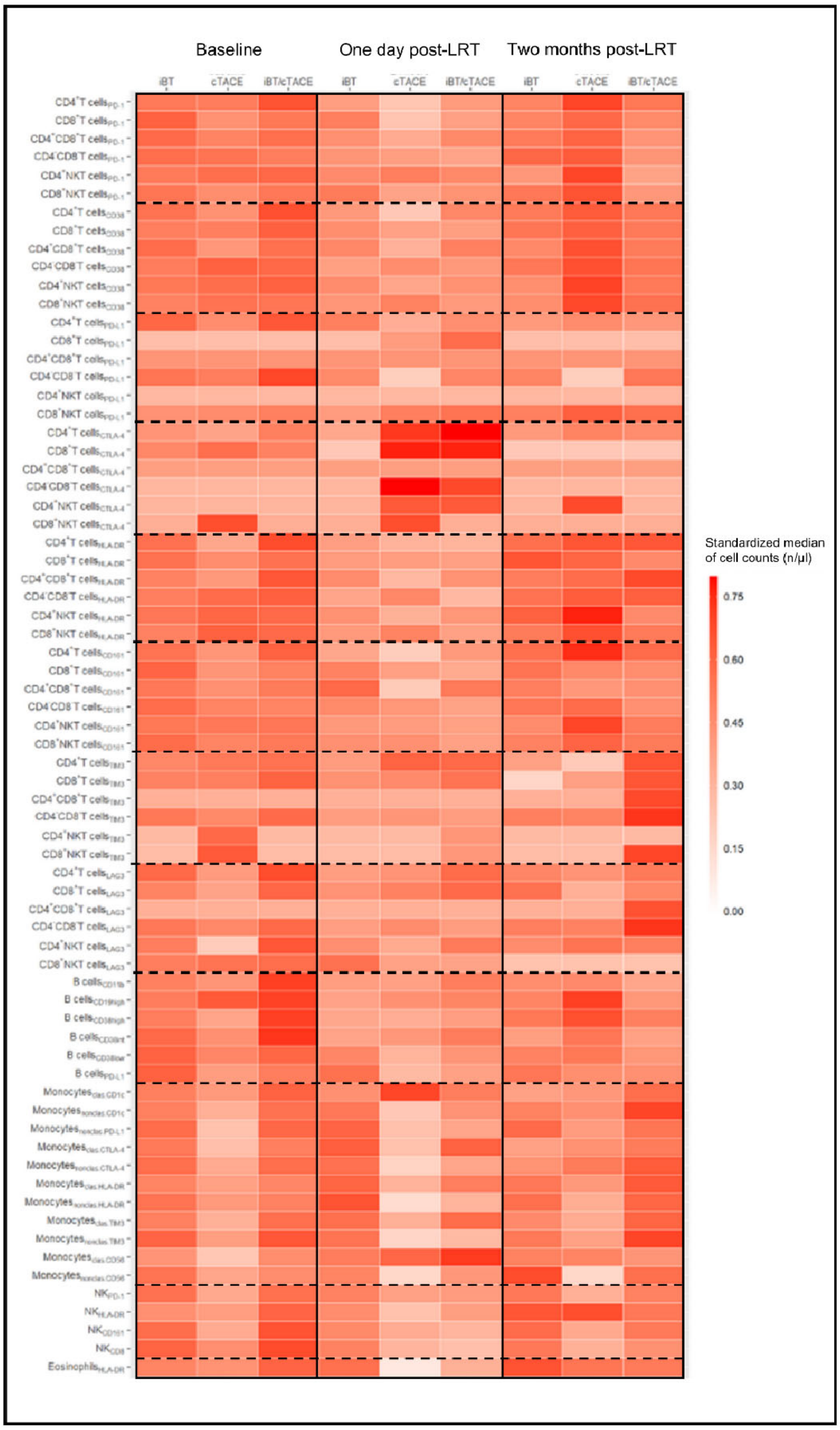
Baseline
One day post-LRT
Two months post-LRT
iBT
cTACE
iBT/cTACE
iBT
cTACE
iBT/cTACE
iBT
cTACE
iBT/cTACE
Standardized median of cell counts (n/µl)
0.75
0.60
0.45
0.30
0.15
0.00

Fig. S5

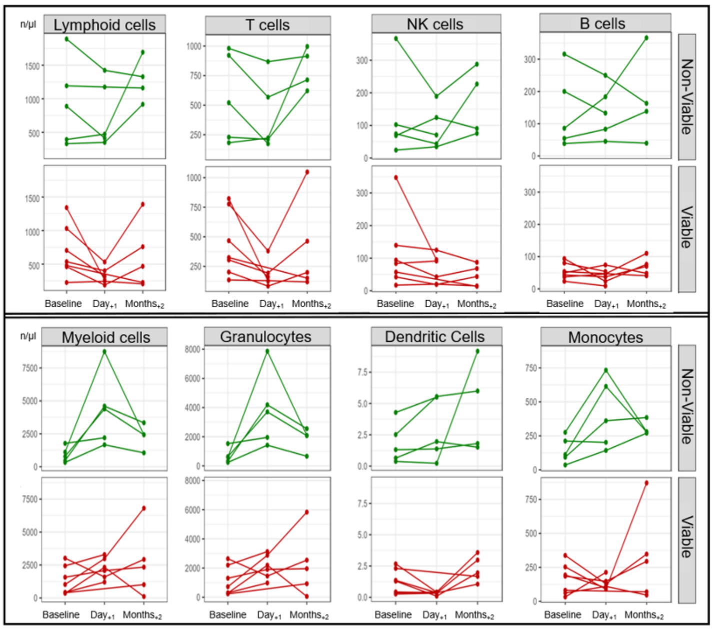

n/µl
Lymphoid cells
T cells
NK cells
B cells
Non-Viable
Viable
Myeloid cells
Granulocytes
Dendritic Cells
Monocytes
Baseline
Day+1
Months+2

Fig. S6

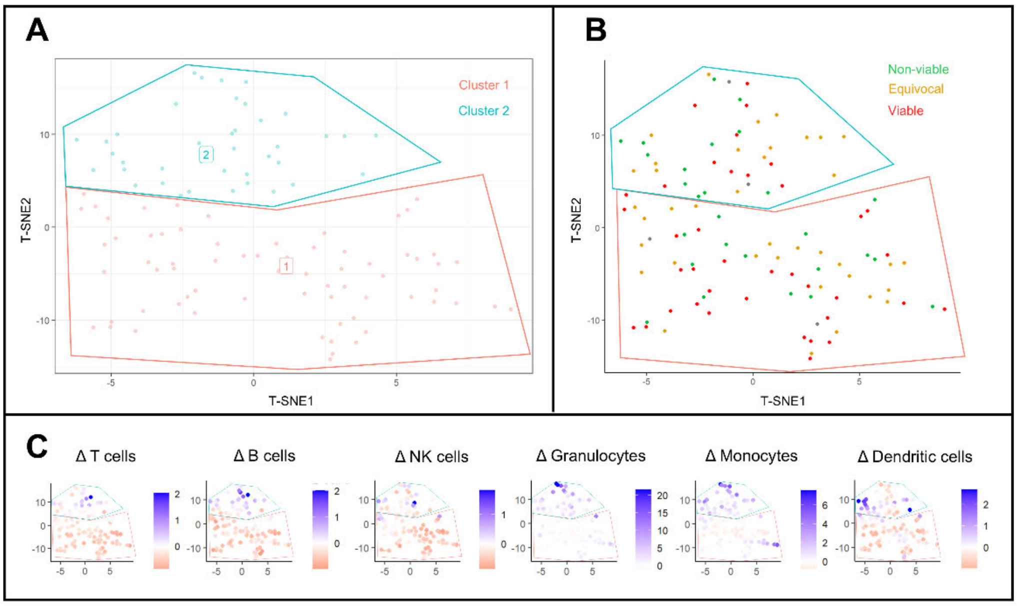

A
Cluster 1
Cluster 2
T-SNE1
T-SNE2
B
Non-viable
Equivocal
Viable
C
Δ T cells
Δ B cells
Δ NK cells
Δ Granulocytes
Δ Monocytes
Δ Dendritic cells

Fig. S7

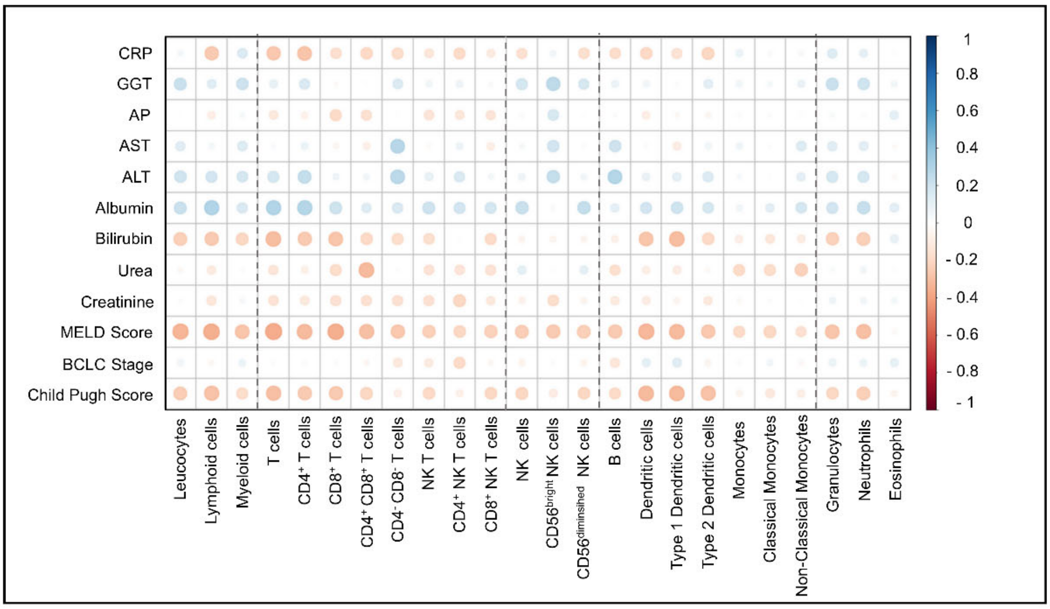
CRP
GGT
AP
AST
ALT
Albumin
Bilirubin
Urea
Creatinine
MELD Score
BCLC Stage
Child Pugh Score
Leucocytes
Lymphoid cells
Myeloid cells
T cells
CD4+ T cells
CD8+ T cells
CD4+ CD8+ T cells
CD4- CD8- T cells
NK T cells
CD4+ NK T cells
CD8+ NK T cells
NK cells
CD56bright NK cells
CD56diminished NK cells
B cells
Dendritic cells
Type 1 Dendritic cells
Type 2 Dendritic cells
Monocytes
Classical Monocytes
Non-Classical Monocytes
Granulocytes
Neutrophils
Eosinophils
1
0.8
0.6
0.4
0.2
0
- 0.2
- 0.4
- 0.6
- 0.8
- 1

Supplementary figure legends

Fig. S1: Gating Strategy. FSC-A and SSC-B-A scatters were used to identify leucocyte cell populations. SSC-A scatter was used to identify single cells vs cell debris, while SSC-H scatter was used to exclude red blood cell products. Next, CD45+ cell populations were gated according to major lymphoid and myeloid cell populations as described in the methods. Red asterisks indicate the use of an exclusion gate instead of an inclusion gate.

Fig. S2 – Immune cell hierarchy. Immune cell populations are mapped with their cell markers (CD - cluster of differentiation proteins) and sorted into hierarchical structure for gating of the flow cytometry results.

Fig. S3 – Alterations of cytokines and chemokines following conventional transarterial chemoembolization (cTACE). Dot plots reveal the absolute changes of pro- and inti-inflammatory cytokines and chemokines one day post-cTACE. The blue dotted line indicates the cohort's median Cohen's D of ± 0.29.

Fig. S4 - Longitudinal changes of activation, exhaustion, and checkpoint molecules on immune cell populations stratified by type of locoregional therapies. Heatmap showing standardized medians (SM, from 0 to 1) of checkpoints, activation, and exhaustion molecules on immune cell populations before and after interstitial brachytherapy (iBT), conventional transarterial chemoembolization (cTACE), or a combined cTACE/iBT. At one day post-LRT, SM of molecules on monocytes and B cells decreased, but SM of CTLA-4 on T cells increased

demonstrating the most profound effect in patients with cTACE. For these patients compared to the other treatment groups, higher expression of PD-1, HLA-DR, and CD161 on T cells and of molecules on B cells were observed at two months post-LRT.

Fig. S5 - Associations of immune cell profiles with tumor response following conventional transarterial chemoembolization. Line graphs show individual immune cell count alterations for completed treatment cycles of conventional transarterial chemoembolization (cTACE, n=27). Responders according to LI-RADS-TRA v2017 (green lines) generally showed higher cell counts of lymphoid and myeloid populations at all time points. Additionally, in responders, lymphoid cells demonstrated a steady increase in the longitudinal course, while in non-responders on follow-up imaging (red lines), lymphoid cells tended to decrease as early as one day but then re-increased at two months after LRT. Regarding myeloid cells, responders showed an increase one day post-LRT, whereas non-responders only showed a slight increase at two months post-LRT.

Fig. S6 - Associations of immune cell profiles with tumor response. Clustering analysis using immune alterations of major immune cell populations as well as CD4+ and CD8+ T cell subsets expressing PD1, PDL-1 and CTLA4 shows a similar clustering (TSNE-pattern) and immune cell subset distribution within two clusters as the results from the clustering analysis without including checkpoint molecules as independent clustering parameters. A) TSNE-clustering analysis based on immune cell alterations of major immune cell populations from baseline to one day post-LRT revealed two clusters of patients with different immunological responses. B) Patients

in the first cluster showed higher rates of remaining tumor viability according to LIRADS TRA v2017, while patients in the second cluster demonstrated more inflamed tumor response with higher rates of equivocal tumor. C) Patients from the first cluster show decreasing populations of T-, B-, NK cells, monocytes, and conventional dendritic cells, while patients from the second cluster showed no trends in lymphoid populations but an increase in conventional dendritic cells, monocytes, and granulocytes.

Fig. S7 - Correlation of baseline immune cell counts with clinical and laboratory patient characteristics. Correlation matrix shows the extent (size and color of the circles) and direction of the correlation of baseline immune cell counts with clinical and laboratory parameters (blue- parallel; red - antiparallel). Spearman correlation did not reveal any strong correlations, however trends in parallel correlations of immune cell counts with albumin, gamma-glutamyl transferase levels (γGT), Child-Pugh, and Model of End-Stage Liver Disease (MELD) score were found, respectively, while anti-parallel correlative trends were mainly observed between immune cell counts and C-reactive protein, bilirubin, urea, creatinine, and the Barcelona Clinic Liver Cancer (BCLC) score, respectively.

Supplementary references

1. Cossarizza A, Chang HD, Radbruch A, Abrignani S, Addo R, Akdis M, et al. Guidelines for the use of flow cytometry and cell sorting in immunological studies (third edition). Eur J Immunol. 2021; 51: 2708-3145.

2. Kapellos TS, Bonaguro L, Gemünd I, Reusch N, Saglam A, Hinkley ER, et al. Human Monocyte Subsets and Phenotypes in Major Chronic Inflammatory Diseases. Front Immunol. 2019; 10: 2035.

3. Poli A, Michel T, Thérésine M, Andrès E, Hentges F, Zimmer J. CD56bright natural killer (NK) cells: an important NK cell subset. Immunology. 2009; 126: 458-65.

4. Mousset CM, Hobo W, Woestenenk R, Preijers F, Dolstra H, van der Waart AB. Comprehensive Phenotyping of T Cells Using Flow Cytometry. Cytometry A. 2019; 95: 647-54.

5. Lencioni R, Petruzzi P, Crocetti L. Chemoembolization of hepatocellular carcinoma. Semin Intervent Radiol. 2013; 30: 3-11.

6. Ricke J, Wust P, Stohlmann A, Beck A, Cho CH, Pech M, et al. CT-guided interstitial brachytherapy of liver malignancies alone or in combination with thermal ablation: phase I-II results of a novel technique. Int J Radiat Oncol Biol Phys. 2004; 58: 1496-505.

7. Bretschneider T, Ricke J, Gebauer B, Streitparth F. Image-guided high-dose-rate brachytherapy of malignancies in various inner organs - technique, indications, and perspectives. J Contemp Brachytherapy. 2016; 8: 251-61.

8. Collettini F, Schnapauff D, Poellinger A, Denecke T, Schott E, Berg T, et al. Hepatocellular carcinoma: computed-tomography-guided high-dose-rate brachytherapy (CT-HDRBT) ablation of large (5-7 cm) and very large (>7 cm) tumours. Eur Radiol. 2012; 22: 1101-9.
